# Supplementary material for: Oil-in-water emulsion adjuvants for pediatric influenza vaccines: a systematic review and meta-analysis
Source: Nat Commun. 2020 Jan 16;11:315. doi: 10.1038/s41467-019-14230-x (PMC6965081; doi:10.1038/s41467-019-14230-x)
Supplement: Supplementary file 1 — Supplementary Information [file 41467_2019_14230_MOESM1_ESM.pdf]

Supplementary Material

Supplement to:

Lin et al. Oil-in-water emulsion adjuvants for pediatric influenza vaccines: a systematic review and meta-analysis

Supplementary Notes

Supplementary Note 1. MEDLINE (PubMed) search strategy

Supplementary Note 2. CENTRAL (Search Manger) search strategy

Supplementary Note 3. Embase (Elsevier) strategy

Supplementary Note 4. CINAHL/Web of Science/Scopus search strategy

Supplementary Note 5. Google Scholar strategy

Supplementary Note 6. WHO ICTRP (Search for clinical trials in children), Grey Literature Report, Open Grey, RePORT, HSRProj, AHRQ Grants On-Line Database, HSRR search strategy

Supplementary Note 7. Clinical Trials.gov search strategy

Supplementary Note 8. List of excluded studies and the reasons for exclusion

Supplementary Tables

Supplementary Table 1. Randomized controlled trials that reported efficacy data

Supplementary Table 2. Randomized controlled trials that reported seroprotection data

Supplementary Table 3. Randomized controlled trials that reported serious adverse events (SAEs)

Supplementary Table 4. Randomized controlled trials that reported neurological events

Supplementary Table 5. Randomized controlled trials that reported reactogenicity data

Supplementary Table 6. Summary of findings: certainty of evidence for effect outcomes

Supplementary Table 7. Summary of findings: certainty of evidence for harm outcomes

Supplementary Figures

Supplementary Figure 1. Forest plot showing the ratios of the seroprotection rate (SPR) against H1N1, defined as a ≥1:40 haemagglutinin-inhibition (HI) titer, 21–28 days after the second dose among children who received adjuvanted versus non-adjuvanted trivalent vaccines (TIV) or quadrivalent vaccines (QIV).

Supplementary Figure 2. Meta-regression showing a linear relationship (slope = 0.98, P <0.001) between the ratios of seroprotection rates against H1N1 after the second dose and the inverse of seroprotection rate in non-adjuvanted arm, with an R^2 of 95.47%

Supplementary Figure 3. Forest plot showing the ratios of the seroprotection rate (SPR) against H3N2, defined as a ≥1:40 haemagglutinin-inhibition (HI) titer, 21–28 days after the second dose among children who received adjuvanted versus non-adjuvanted trivalent vaccines (TIV) or quadrivalent vaccines (QIV).

Supplementary Figure 4. Meta-regression showing a linear relationship (slope = 0.91, P <0.001) between the ratios of seroprotection rates against H3N2 after the second dose and the inverse of seroprotection rate in non-adjuvanted arm, with an R^2 of 88.92%

Supplementary Figure 5. Forest plot showing the ratios of the seroprotection rate (SPR) against influenza B, defined as a ≥1:40 haemagglutinin-inhibition (HI) titer, 21–28 days after the first dose among children who received adjuvanted versus non-adjuvanted trivalent vaccines (TIV) or quadrivalent vaccines (QIV).

Supplementary Figure 6. Forest plot showing the risk ratios of pain/tenderness at the injection site during the 7 days after the first dose among children who received adjuvanted versus non-adjuvanted trivalent vaccines (TIV) or quadrivalent vaccines (QIV).

Supplementary Figure 7. Forest plot showing the risk ratios of fever during the 7 days

after the first dose among children who received adjuvanted versus non-adjuvanted trivalent vaccines (TIV) or quadrivalent vaccines (QIV).

Supplementary Figure 8. Forest plot showing the risk ratios of pain/tenderness at the injection site during the 7 days after the second dose among children who received adjuvanted versus non-adjuvanted trivalent vaccines (TIV) or quadrivalent vaccines (QIV).

Supplementary Figure 9. Forest plot showing the risk ratios of fever during the 7 days after the second dose among children who received adjuvanted versus non-adjuvanted trivalent vaccines (TIV) or quadrivalent vaccines (QIV).

Supplementary Figure 10. Funnel plot with pseudo 95% confidence limits.

Supplementary Figure 11. Risk of bias summary.

Supplementary Figure 12. Risk of bias graph.

Supplementary Figure 13. Sensitivity analysis by excluding Langley (2012): forest plot showing the ratios of the seroprotection rate (SPR) against H1N1, defined as a ≥1:40 haemagglutinin-inhibition (HI) titer, 21–28 days after the first dose among children who received adjuvanted versus non-adjuvanted trivalent vaccines (TIV) or quadrivalent vaccines (QIV).

Supplementary Figure 14. Sensitivity analysis by excluding Langley (2012): meta-regression showing a linear relationship (slope = 0.79, P <0.001) between the ratios of seroprotection rates against H1N1 after the first dose and the inverse of seroprotection rate in non-adjuvanted arm, with an R^2 of 99.81%.

Supplementary Figure 15. Sensitivity analysis by excluding Zedda (2015): forest plot showing the ratios of the seroprotection rate (SPR) against H3N2, defined as a ≥1:40 haemagglutinin-inhibition (HI) titer, 21–28 days after the second dose among children who received

adjuvanted versus non-adjuvanted trivalent vaccines (TIV) or quadrivalent vaccines (QIV).

Supplementary Figure 16. Sensitivity analysis by excluding Zedda (2015): meta-regression showing a linear relationship (slope = 0.91, P <0.001) between the ratios of seroprotection rates against H3N2 after the second dose and the inverse of seroprotection rate in non-adjuvanted arm, with an R^2 of 88.54%.

Supplementary Figure 17. Sensitivity analysis by excluding Zedda (2015): forest plot showing the ratios of the seroprotection rate (SPR) against influenza B, defined as a ≥1:40 haemagglutinin-inhibition (HI) titer, 21–28 days after the second dose among children who received adjuvanted versus non-adjuvanted trivalent vaccines (TIV) or quadrivalent vaccines (QIV).

Supplementary Figure 18. Sensitivity analysis by excluding Zedda (2015): meta-regression showing a linear relationship (slope = 0.88, P <0.001) between the ratios of seroprotection rates against influenza B after the second dose and the inverse of seroprotection rate in non-adjuvanted arm, with an R^2 of 99.63%.

Supplementary Figure 19. Sensitivity analysis by excluding the 12 trials which did not compare the same vaccine with or without adjuvants (using adjuvanted and non-adjuvanted vaccines from different manufacturers, or using antigen-sparing design with reduced antigen dose in adjuvanted vaccines): forest plot showing the risk ratios of RT-PCR-confirmed influenza among children who received adjuvanted versus non-adjuvanted trivalent vaccines (TIV) or quadrivalent vaccines (QIV).

Supplementary Figure 20. Sensitivity analysis by excluding the 12 trials which did not compare the same vaccine with or without adjuvants (using adjuvanted and non-adjuvanted vaccines from different manufacturers, or using antigen-sparing design with reduced antigen dose in adjuvanted vaccines): forest plot showing the

ratios of the seroprotection rate (SPR) against H1N1, defined as a ≥1:40 haemagglutinin-inhibition (HI) titer, 21–28 days after the first dose among children who received adjuvanted versus non-adjuvanted trivalent vaccines (TIV) or quadrivalent vaccines (QIV).

Supplementary Figure 21. Sensitivity analysis by excluding the 12 trials which did not compare the same vaccine with or without adjuvants (using adjuvanted and non-adjuvanted vaccines from different manufacturers, or using antigen-sparing design with reduced antigen dose in adjuvanted vaccines): meta-regression showing a linear relationship (slope = 0.90, $P < 0.001$) between the ratios of the seroprotection rate against H1N1 after the first dose and the inverse of seroprotection rate in non-adjuvanted arm, with an R^2 of 99.56%.

Supplementary Figure 22. Sensitivity analysis by excluding the 12 trials which did not compare the same vaccine with or without adjuvants (using adjuvanted and non-adjuvanted vaccines from different manufacturers, or using antigen-sparing design with reduced antigen dose in adjuvanted vaccines): forest plot showing the ratios of the seroprotection rate (SPR) against H3N2, defined as a ≥1:40 haemagglutinin-inhibition (HI) titer, 21–28 days after the first dose among children who received adjuvanted versus non-adjuvanted trivalent vaccines (TIV) or quadrivalent vaccines (QIV).

Supplementary Figure 23. Sensitivity analysis by excluding the 12 trials which did not compare the same vaccine with or without adjuvants (using adjuvanted and non-adjuvanted vaccines from different manufacturers, or using antigen-sparing design with reduced antigen dose in adjuvanted vaccines): meta-regression showing a linear relationship (slope = 0.95, $P < 0.001$) between the ratios of the seroprotection rate against H3N2 after the first dose and the inverse of seroprotection rate in non-adjuvanted arm, with

an R^2 of 100%.

Supplementary Figure 24. Sensitivity analysis by excluding the 12 trials which did not compare the same vaccine with or without adjuvants (using adjuvanted and non-adjuvanted vaccines from different manufacturers, or using antigen-sparing design with reduced antigen dose in adjuvanted vaccines): forest plot showing the ratios of the seroprotection rate (SPR) against influenza B, defined as a ≥1:40 haemagglutinin-inhibition (HI) titer, 21–28 days after the second dose among children who received adjuvanted versus non-adjuvanted trivalent vaccines (TIV) or quadrivalent vaccines (QIV).

Supplementary Figure 25. Sensitivity analysis by excluding the 12 trials which did not compare the same vaccine with or without adjuvants (using adjuvanted and non-adjuvanted vaccines from different manufacturers, or using antigen-sparing design with reduced antigen dose in adjuvanted vaccines): meta-regression showing a linear relationship (slope=0.83, $p<0.001$) between the ratios of the seroprotection rate against influenza B after the second dose and the inverse of seroprotection rate in non-adjuvanted arm, with an R^2 of 99.84%.

Supplementary Figure 26. Sensitivity analysis by excluding the 12 trials which did not compare the same vaccine with or without adjuvants (using adjuvanted and non-adjuvanted vaccines from different manufacturers, or using antigen-sparing design with reduced antigen dose in adjuvanted vaccines): forest plot showing the risk ratios of serious adverse events during the follow-up time after the vaccinations among children who received adjuvanted versus non-adjuvanted trivalent vaccines (TIV) or quadrivalent vaccines (QIV).

Supplementary Figure 27. Sensitivity analysis by excluding the 12 trials which did not compare the same vaccine with or without adjuvants (using

adjuvanted and non-adjuvanted vaccines from different manufacturers, or using antigen-sparing design with reduced antigen dose in adjuvanted vaccines): forest plot showing the risk ratios of neurological events during the follow-up time after the vaccinations among children who received adjuvanted versus non-adjuvanted trivalent vaccines (TIV) or quadrivalent vaccines (QIV).

Supplementary Figure 28. Sensitivity analysis by excluding 2 data points (both reported in Diallo 2018) with an inverse of seroprotection rate in non-adjuvanted arm larger than 5: Meta-regression still showed a linear relationship (slope = 0.90, P <0.001) between the ratios of seroprotection rates against H1N1 after the first dose and the inverse of seroprotection rate in non-adjuvanted arm, with an R^2 of 98.67%.

Supplementary Figure 29. Sensitivity analysis by excluding 2 data points (Vesikari 2011 and Della Cioppa 2011) with an inverse of seroprotection rate in non-adjuvanted arm larger than 5: Meta-regression results became imprecise (slope = 0.47, P=0.075) between the ratios of seroprotection rates against H3N2 after the first dose and the inverse of seroprotection rate in non-adjuvanted arm, with an R^2 of 42.98%, after the sample size decreased to only seven.

Supplementary Figure 30. Sensitivity analysis by excluding 2 data points (Vesikari 2011 and Della Cioppa 2011), with an inverse of seroprotection rate in non-adjuvanted arm larger than 5: Meta-regression still showed a linear relationship (slope = 0.86, P <0.001) between the ratios of seroprotection rates against influenza B after the first dose and the inverse of seroprotection rate in non-adjuvanted arm, with an R^2 of 96.21%.

Supplementary Note 1. MEDLINE (PubMed) search strategy

No.	Query
#1	((influenza vaccin* [Text Word]) OR ((influenza [Text Word] OR flu [Text Word]) and (vaccin* [Text Word] OR immuni* [Text Word] or inocula* [Text Word] OR efficacy [Text Word] OR effectiveness [Text Word])))
#2	"influenza vaccines" [MeSH] OR ("influenza, human/immunology" [MeSH] OR "influenza, human/prevention and control" [MeSH])
#3	#2 AND #3
#4	(randomized controlled trial [pt] OR controlled clinical trial [pt] OR randomized [tiab] OR placebo [tiab] OR drug therapy [sh] OR randomly [tiab] OR trial [tiab] OR groups [tiab]
#5	("cross over" OR "crossover" OR "follow Up" OR ("cross-over studies [MeSH] OR "follow-up studies" [MeSH] OR ""prospective studies" [MeSH]) OR ("time series" OR "interrupted time series" OR (placebo* OR random* OR "double blind" OR "single blind" OR clinical trial* OR trial design) OR ("case-control studies" [MeSH] OR (cases [Title/Abstract] AND controls [Title/Abstract])) OR ("cohort studies" [MeSH] OR cohort*) OR ("comparative study" [Publication Type]) OR ("before after" [Title/Abstract] OR "before-after" [Title/Abstract] OR "before/after" [Title/Abstract] OR "before and after" [Title/Abstract]) OR (volunteer* [Title/Abstract]) OR (control* [Text Word] AND evaluation [Text Word]) OR (longitudinal [Text Word]) OR (retrospective* [Text Word])
#6	#5 OR #6
#7	#4 AND #7
#8	child* OR preschool* OR school* OR young OR adolescent* OR infant* OR toddler* OR pediatric* OR paediatric* OR infant*
#9	#8 AND #9
#10	(adjuvant [All Fields] OR adjuvanted [All Fields] OR adjuvants [All Fields]) OR ("MF59 oil emulsion" [All Fields] OR MF59 [Text Word]) OR AS03 [All Fields]
#11	#10 AND #11

Supplementary Note 2. CENTRAL (Search Manger) search strategy

No.	Query
#1	adjuvant* OR MF59 OR AS03
#2	(influenza vaccin*) OR ((influenza or flu*) AND (vaccin* OR immun* OR inocul*))
#3	child* OR infant* OR toddler*
#4	#1 AND #3 AND #4

Supplementary Note 3. Embase (Elsevier) strategy

No.	Query
#1	influenza AND vaccin* AND (influenza OR flu*) AND (vaccin* OR immun* OR inocul*)
#2	randomized controlled trial/exp OR single blind procedure/exp OR double blind procedure/exp OR crossover procedure/exp
#3	random*:ab,ti OR placebo*:ab,ti OR factorial*:ab,ti OR crossover*:ab,ti OR cross -over:ab,ti OR cross over:ab,ti OR allocat*:ab,ti OR volunteer*:ab,ti OR ((singl*:ab,ti OR doubl*:ab,ti) AND (blind*:ab,ti OR mask*:ab,ti))
#4	#2 OR #3
#5	#1 AND #4
#6	child*:de,ab,ti OR preschool*:de,ab,ti OR school*:de,ab,ti OR young:de,ab,ti OR adolescent*:de,ab,ti OR toddler*:de,ab,ti OR pediatric*:de,ab,ti OR paediatric*:de,ab,ti OR infant*:de,ab,ti
#7	#5 AND #6
#8	adjuvant* OR MF59 OR AS03
#9	#7 AND #8

Supplementary Note 4. CINAHL/ Web of Science/ Scopus search strategy

(adjuvant* OR MF59 OR AS03) AND (influenza vaccin*) AND (influenza or flu*) AND (vaccin* OR immun* OR inocul*) AND (child* OR infant* OR toddler* OR pediatric* OR pacdiatric*)

Supplementary Note 5. Google Scholar strategy

(adjuvant* OR MF59 OR AS03) AND (influenza vaccin*) AND (influenza or flu*) AND (vaccin* OR immun* OR inocul*) AND (child* OR infant* OR toddler* OR pediatric* OR paediatric*) –avian –adult –elderly –animal –cell

Supplementary Note 6. WHO ICTRP (Search for clinical trials in children), Grey Literature Report, Open Grey, RePORT, HSRProj, AHRQ Grants On-Line Database, HSRR search strategy

(Vaccine* AND influenza) AND (adjuvant* OR MF59 OR AS03)

(immuni* AND influenza) AND (adjuvant* OR MF59 OR AS03)

(inocul* AND influenza) AND (adjuvant* OR MF59 OR AS03)

(vaccine* AND flu) AND (adjuvant* OR MF59 OR AS03)

(immuni* AND flu) AND (adjuvant* OR MF59 OR AS03)

(inocul* AND flu) AND (adjuvant* OR MF59 OR AS03)

Supplementary Note 7. Clinical Trials.gov search strategy

(adjuvant OR MF59 OR AS03) AND (influenza vaccine) AND (vaccination OR immunization OR inoculation) AND (child OR infant OR toddler OR pediatric OR paediatric)

Supplementary Note 8. List of excluded studies and the reasons for exclusion

Trial	Reason for exclusion
Höschler 2018	Not under 9 years of age
Canelle 2016	Not under 9 years of age
Knuf 2015	Duplicated data
Örtqvist 2015	Not under 9 years of age
Sicilia 2015	Not trials that compared adjuvanted vs. non-adjuvanted vaccines
Thebault 2015	Not trials that compared adjuvanted vs. non-adjuvanted vaccines
Vesikari 2015	Not trials that compared adjuvanted vs. non-adjuvanted vaccines
Hoschler 2014	Not trials that compared adjuvanted vs. non-adjuvanted vaccines
Knuf 2014	Duplicated data
Martinez 2014	Not trials that compared adjuvanted vs. non-adjuvanted vaccines
Poder 2014	Not under 9 years of age
Winstone 2014	Not under 9 years of age
Buynder 2013	Not trials that compared adjuvanted vs. non-adjuvanted vaccines
Launay 2013	Not trials that compared adjuvanted vs. non-adjuvanted vaccines
Baselli 2012	Not trials that compared adjuvanted vs. non-adjuvanted vaccines
Fukase 2012	Not trials that compared adjuvanted vs. non-adjuvanted vaccines
Kulkarni 2012	Not under 9 years of age
Langley 2012	Not trials that compared adjuvanted vs. non-adjuvanted vaccines

Trial	Reason for exclusion
Nassim 2012	Not trials that compared adjuvanted vs. non-adjuvanted vaccines
Nohynek 2012	Not trials that compared adjuvanted vs. non-adjuvanted vaccines
Örtqvist 2012	Not trials that compared adjuvanted vs. non-adjuvanted vaccines
Saitoh 2012	Not trials that compared adjuvanted vs. non-adjuvanted vaccines
Walker 2012	Not trials that compared adjuvanted vs. non-adjuvanted vaccines
Andrews 2011	Not trials that compared adjuvanted vs. non-adjuvanted vaccines
Banzhoff 2011	Not trials that compared adjuvanted vs. non-adjuvanted vaccines
Bardage 2011	Not trials that compared adjuvanted vs. non-adjuvanted vaccines
Black 2011	Duplicated data
Cristiani 2011	Not trials that compared adjuvanted vs. non-adjuvanted vaccines
Esposito 2011	Not under 9 years of age
Esposito 2011	Not trials that compared adjuvanted vs. non-adjuvanted vaccines
Gilca 2011	Not trials that compared adjuvanted vs. non-adjuvanted vaccines
Griffin 2011	Not trials that compared adjuvanted vs. non-adjuvanted vaccines
Kries 2011	Not trials that compared adjuvanted vs. non-adjuvanted vaccines
Ortqvist 2011	Not trials that compared adjuvanted vs. non-adjuvanted vaccines
Parretta 2011	Not trials that compared adjuvanted vs. non-adjuvanted vaccines
Scheifele 2011	Not trials that compared adjuvanted vs. non-adjuvanted vaccines
Sicilia 2011	Not trials that compared adjuvanted vs. non-adjuvanted vaccines

Trial	Reason for exclusion
Skowronski 2011	Not trials that compared adjuvanted vs. non-adjuvanted vaccines
Uphoff 2011	Not trials that compared adjuvanted vs. non-adjuvanted vaccines
Whalley2011	Not trials that compared adjuvanted vs. non-adjuvanted vaccines
Buynder 2010	Not under 9 years of age
Carmona 2010	Not trials that compared adjuvanted vs. non-adjuvanted vaccines
Gagnon 2010	Not trials that compared adjuvanted vs. non-adjuvanted vaccines
Waddington 2010	Duplicated data
Yasuda 2010	Not trials that compared adjuvanted vs. non-adjuvanted vaccines
Liang 2009	Not under 9 years of age
Zhu 2009	Not under 9 years of age
Vesikari 2008	Not trials that compared adjuvanted vs. non-adjuvanted vaccines
Marchetti 2007	Not trials that compared adjuvanted vs. non-adjuvanted vaccines
Woodhour 1969	Not trials that compared adjuvanted vs. non-adjuvanted vaccines
Stokes 1969	Not under 9 years of age
Davenport 1968	Not trials that compared adjuvanted vs. non-adjuvanted vaccines
Quilligan 1966	Not under 9 years of age

Supplementary Table 1. Randomized controlled trials that reported efficacy data against RT-PCR confirmed influenza

**Study**	**Study design**	**Setting**	**Vaccine strain**	**Age of participants**	**Intervention /manufacturer/ HA content per ml (n)**	**Comparator /manufacturer/ HA content per ml (n)**	**Follow-up time (day)**	**Funding source**
Vesikari 2011	Phase 3, Observer-blind, Randomized	Study centers in Germany and Finland	H1N1, H3N2, B	6-35 months	MF59-adjuvanted /Novartis/30 µg (n=1,103)	Subunit and split trivalent inactivated /Novartis/30 µg (n=995)	6-12 months after each vaccination	Novartis
				36-71 months	MF59-adjuvanted /Novartis/30 µg (n=834)	Subunit and split trivalent inactivated /Novartis/30 µg (n=777)		
Nolan 2014*	Phase 3, Observer-blind, Randomized	17 centers in Australia, Brazil, Colombia, Costa Rica, Mexico, Philippines, Singapore, Thailand	H1N1	6-35 months	AS03-adjuvanted /GSK/7.5 µg (n=569)	Non-adjuvanted /GSK/30 µg (n=561)	385 days after randomization	GlaxoSmithKline

Study	Study design	Setting	Vaccine strain	Age of participants	Intervention /manufacturer/ HA content per ml (n)	Comparator /manufacturer/ HA content per ml (n)	Follow-up time (day)	Funding source
Vesikari 2018†	Phase 3 Observer-blind, Randomized	146 sites including hospitals, clinics and clinician offices in Finland, USA, Canada, Italy, Poland, Spain, Philippines, Thailand, Taiwan	H1N1, H3N2, B	6-23 months	MF59-adjuvanted, subunit quadrivalent, inactivated /Seqirus/30 µg (n=1,299)	Non-adjuvanted inactivated /Sanofi Pasteur/30 µg (n=1,339)	180 days after last vaccination	Seqirus UK Ltd.
				2-5 years	MF59-adjuvanted, subunit quadrivalent, inactivated /Seqirus/30 µg (n=3,979)	Non-adjuvanted inactivated /Sanofi Pasteur/30 µg (n=3,854)		

*This trial compared adjuvanted and non-adjuvanted vaccine formulations with unequal hemagglutinin antigen dose. The hemagglutinin antigen dose was reduced in the adjuvanted arm (antigen-sparing design).

†This trial compared adjuvanted influenza vaccines and non-adjuvanted influenza vaccines from different manufacturers.

Supplementary Table 2. Randomized controlled trials that reported seroprotection data

Study	Study design	Setting	Vaccine strain	Age of participants	Intervention /manufacturer/HA content per ml (n)	Comparator /manufacturer/HA content per ml (n)	Follow-up time (day)	Funding source
Vesikari 2009 † (PIDJ)	Observer-blind Randomized	Finland	H1N1, H3N2, B	6-35 months	MF59-adjuvanted subunit /Novartis/7.5 µg (n=104)	Non-adjuvanted split /Sanofi Pasteur/7.5 µg (n=118)	21 d after 2nd dose	Novartis
Vesikari 2009† (Vaccine)	Observer-blind Randomized	Finland	H1N1, H3N2, B	16-47 months	MF59-adjuvanted subunit /Novartis/30 µg (n=41)	Non-adjuvanted split /Sanofi Pasteur/30 µg (n=40)	21 d after 1st dose	Novartis
Arguedas 2011*†	Phase 3 Open-label Randomized	Costa Rica	H1N1	3-8 years	MF59-adjuvanted /Novartis/15 µg (n=46)	Non-adjuvanted /Novartis/30 µg (n=75)	21 d after 1st and 2nd doses	Novartis
Della Cioppa 2011†	Observer-blind Randomized	15 study centers in Finland and Belgium	H1N1, H3N2, B	6-35 months	MF59-adjuvanted /Novartis/30 µg (n=37)	Non-adjuvanted /Sanofi Pasteur/30 µg (n=43)	21 d after 1st and 2nd doses	Novartis
Vesikari 2011	Phase 3 Observer-blind Randomized	Germany, Finland	H1N1, H3N2, B	6-35 months	MF59-adjuvanted /Novartis/30 µg (n=1103)	Non-adjuvanted subunit /Novartis/30 µg (n=995)	28 d after 1st and 2nd doses	Novartis
				36-71 months	MF59-adjuvanted /Novartis/30 µg (n=834)	Non-adjuvanted subunit /Novartis/30 µg (n=777)	28 d after 1st and 2nd doses	

Study	Study design	Setting	Vaccine strain	Age of participants	Intervention /manufacturer/HA content per ml (n)	Comparator /manufacturer/HA content per ml (n)	Follow-up time (day)	Funding source
Block 2012	Phase 2<br>Phase 3<br>Double-blind<br>Randomized	29 sites in USA and Mexico	H1N1	6-35 months	MF59-adjuvanted monovalent subunit /Novartis/30 µg (n=127)	Non-adjuvanted monovalent subunit /Novartis/30 µg (n=124)	21 d after 1st and 2nd doses	Novartis, Federal funds
Langley 2012*	Double-blind<br>Randomized	12 centers in Canada	H1N1	6-11 months	AS03-adjuvanted monovalent /GSK/15 µg (n=5)	Non-adjuvanted monovalent /GSK/30 µg (n=4)	21 d after 1st and 2nd doses	GlaxoSmithKline, US Department of Health and Human Service
				12-35 months	AS03-adjuvanted monovalent /GSK /15 µg (n=15)	Non-adjuvanted monovalent /GSK/30 µg (n=16)	21 d after 1st and 2nd doses	
				3-8 years	AS03-adjuvanted monovalent /GSK/15 µg (n=39)	Non-adjuvanted monovalent /GSK/30 µg (n=37)	21 d after 1st and 2nd doses	
Nassim 2012	Phase 2<br>phase 3<br>Single-blind<br>Randomized	34 centers in USA	H1N1	3-8 years (antigen 7.5µg)	MF59-adjuvanted monovalent /Novartis/30 µg (n=156)	Non-adjuvanted monovalent /Novartis/30 µg (n=156)	21 d after 1st and 2nd doses	US CDC

Study	Study design	Setting	Vaccine strain	Age of participants	Intervention /manufacturer/HA content per ml (n)	Comparator /manufacturer/HA content per ml (n)	Follow-up time (day)	Funding source
				3-8 years (antigen 15 μg)	MF59-adjuvanted monovalent /Novartis/30 μg (n=156)	Non-adjuvanted monovalent /Novartis/30 μg (n=155)	21 d after 1st and 2nd doses	
Nolan 2014†	Phase 3 Observer-blind Randomized	32 sites in Argentina, Australia, Chile, Philippines, South Africa	H1N1, H3N2, B	6-71 months	MF59-adjuvanted /Novartis/30 μg (n=266)	Non-adjuvanted /Sanofi Pasteur/30 μg (n=389)	21 d after 2nd dose	Novartis
Nolan 2014*	Phase 3 Observer-blind Randomized	17 centers in Australia, Brazil, Colombia, Costa Rica, Mexico, Philippines, Singapore, Thailand	H1N1	6-35 months	AS03-adjuvanted monovalent /GSK/7.5 μg (n=233)	Non-adjuvanted monovalent /GSK/30 μg (n=223)	21 d after 2nd dose	GlaxoSmithKline
Solares 2014†	phase 2 Observer-blind Randomized	5 sites in Guatemala	H1N1, H3N2, B	6-35 months	MF59-adjuvanted TIV /Novartis/30 μg (n=97)	Non-adjuvanted TIV /Sanofi Pasteur/30 μg (n=102)	21 d after 1st and 2nd doses	Novartis

Study	Study design	Setting	Vaccine strain	Age of participants	Intervention /manufacturer/HA content per ml (n)	Comparator /manufacturer/HA content per ml (n)	Follow-up time (day)	Funding source
				36-59 months	MF59-adjuvanted TIV /Novartis/30 µg (n=23)	Non-adjuvanted TIV /Sanofi Pasteur/30 µg (n=20)	21 d after 1st and 2nd doses	
Knuf 2015*	Single-blind Randomized	13 centers in Germany, Belgium, Dominican Republic, Netherlands	H1N1	12-35 months	MF59-adjuvanted monovalent /Novartis/7.5 µg (n=53)	Non-adjuvanted monovalent /Novartis/15 µg (n=25)	21 d after 1st and 2nd doses	Novartis
				3-8 years	MF59-adjuvanted monovalent /Novartis/7.5 µg (n=60)	Non-adjuvanted monovalent /Novartis/15 µg (n=31)	21 d after 1st and 2nd doses	
Vesikari 2015	Phase 3b Observer-blind Randomized	15 sites across Finland Finland	H1N1, H3N2, B	6-71 months (ATIV primed)	MF59-adjuvanted TIV /Novartis/30 µg (n=17)	Non-adjuvanted TIV /Novartis/30 µg (n=12)	22 d after 1st dose	Novartis
				6-71 months (TIV primed)	MF59-adjuvanted TIV /Novartis/30 µg (n=23)	Non-adjuvanted TIV /Novartis/30 µg (n=24)	22 d after 1st dose	

Study	Study design	Setting	Vaccine strain	Age of participants	Intervention /manufacturer/HA content per ml (n)	Comparator /manufacturer/HA content per ml (n)	Follow-up time (day)	Funding source
Zedda 2015	Randomized	Belgium	H1N1, H3N2, B	6-35 months	MF59-adjuvanted /Novartis/30 µg (n=25)	Non-adjuvanted /Novartis/30 µg (n=30)	21 d after 2nd dose	Novartis
Vesikari 2018†	Phase 3 Observer-blind, Randomized	146 sites including hospitals, clinics and clinician offices in Finland, USA, Canada, Italy, Poland, Spain, Philippines, Thailand, Taiwan	H1N1, H3N2, B	6-60 months	MF59-adjuvanted, subunit quadrivalent, inactivated /Seqirus/30 µg (n=922)	Non-adjuvanted inactivated /Sanofi Pasteur/30 µg (n=866)	28 d after 1st and 2nd doses	Seqirus UK Ltd.
Cruz-Valdez 2018	Phase 3 Observer-blind randomized	Mexico	H1N1, H3N2, B	6-71 months	MF59-adjuvanted TIV /Novartis/30 µg (n=114)	Non-adjuvanted TIV /Novartis/30 µg (n=112)	21 d after 2nd dose	Novartis
Diallo 2018†	Observer-blind randomized	Senegal	H1N1, H3N2, B	6-11 months	MF59-adjuvanted TIV /Novartis/30 µg (n=30)	Non-adjuvanted TIV /Sanofi Pasteur/30 µg (n=26)	28 d after 1st and 2nd doses	US CDC
				12-35 months	MF59-adjuvanted TIV /Novartis/30 µg (n=31)	Non-adjuvanted TIV /Sanofi Pasteur/30 µg (n=34)	28 d after 1st and 2nd doses	

Study	Study design	Setting	Vaccine strain	Age of participants	Intervention /manufacturer/HA content per ml (n)	Comparator /manufacturer/HA content per ml (n)	Follow-up time (day)	Funding source
				36-71 months	MF59-adjuvanted TIV /Novartis/30 μg (n=38)	Non-adjuvanted TIV /Sanofi Pasteur/30 μg (n=33)	28 d after 1st and 2nd doses	

*This trial compared adjuvanted and non-adjuvanted vaccine formulations with unequal hemagglutinin antigen dose. The hemagglutinin antigen dose was reduced in the adjuvanted arm (antigen-sparing design).

†This trial compared adjuvanted influenza vaccines and non-adjuvanted influenza vaccines from different manufacturers.

Supplementary Table 3. Randomized controlled trials that reported serious adverse events (SAEs)

Study	Age of participants	Intervention /manufacturer/HA content per ml (n)	Comparator /manufacturer/HA content per ml (n)	Follow-up time	Results
Vesikari 2009 † (PIDJ)	6-35 months	MF59-adjuvanted subunit /Novartis/30 µg (n=130)	Non-adjuvanted split /Sanofi Pasteur/30 µg (n=139)	Up to 3 weeks	2 subjects in adjuvanted arm, 6 subjects in non-adjuvanted arm.
Vesikari 2009† (Vaccine)	16-47 months	MF59-adjuvanted subunit /Novartis/30 µg (n=43)	Non-adjuvanted split /Sanofi Pasteur/30 µg (n=46)	Up to 6 months	0 subjects in adjuvanted arm, 0 subjects in non-adjuvanted arm.
Waddington 2010*†	Waddington 2010	AS03-adjuvanted split virion /GSK/1.875 µg (n=270)	Non-adjuvanted whole virion /Baxter/3.75 µg (n=279)	Did not report follow-up time for SAE	Did not report SAE data
Arguedas 2011*	3-8 years	MF59-adjuvanted /Novartis/15 µg (n=56)	Non-adjuvanted /Novartis/30 µg (n=54)	Up to 43 days	0 subjects in adjuvanted arm,1 subjects in non-adjuvanted arm.
Della Cioppa 2011†	6-35 months	MF59-adjuvanted /Novartis/30 µg (n=37)	Non-adjuvanted /Sanofi Pasteur/30 µg (n=43)	Up to 50 days	4 subjects in adjuvanted arm, 3subjects in non-adjuvanted arm.
Vesikari 2011	6-71 months	MF59-adjuvanted /Novartis/30 µg (n=2,012)	Non-adjuvanted subunit /Novartis/30 µg (n=1,846)	Up to 12 months	2 subjects in adjuvanted arm, 2 subjects in non-adjuvanted arm.

Study	Age of participants	Intervention /manufacturer/HA content per ml (n)	Comparator /manufacturer/HA content per ml (n)	Follow-up time	Results
Block 2012	6-35 months	MF59-adjuvanted monovalent subunit /Novartis/30 µg (n=161)	Non-adjuvanted monovalent subunit /Novartis/30 µg (n=161)	Up to 12 months	6 subjects in adjuvanted arm, 16 subjects in non-adjuvanted arm.
Langley 2012*	6 months-8 years	AS03-adjuvanted monovalent /GSK/15 µg (n=59)	Non-adjuvanted monovalent /GSK/30 µg (n=57)	Up to 385 days	4 subjects in adjuvanted arm, 0 subjects in non-adjuvanted arm.
Nassim 2012	3-8 years	MF59-adjuvanted monovalent /Novartis/30 µg (n=334)	Non-adjuvanted monovalent /Novartis/30 µg (n=334)	Up to 387 days	6 subjects in adjuvanted arm, 6 subjects in non-adjuvanted arm.
Nolan 2014†	6-71 months	MF59-adjuvanted /Novartis/30 µg (n=3,123)	Non-adjuvanted /Sanofi Pasteur/30 µg (n=1,474)	Up to 394 days	123 subjects in adjuvanted arm, 58 subjects in non-adjuvanted arm.
Nolan 2014*	6-35 months	AS03-adjuvanted monovalent /GSK/7.5 µg (n=2,048)	Non-adjuvanted monovalent /GSK/30 µg (n=2,049)	Up to 385 days	76 subjects in adjuvanted arm, 68 subjects in non-adjuvanted arm.

Study	Age of participants	Intervention /manufacturer/HA content per ml (n)	Comparator /manufacturer/HA content per ml (n)	Follow-up time	Results
Solares 2014†	6-59 months	MF59-adjuvanted TIV /Novartis/30 µg (n=180)	Non-adjuvanted TIV /Sanofi Pasteur/30 µg (n=180)	Up to 211 days	0 subjects in adjuvanted arm, 2 subjects in non-adjuvanted arm.
Knuf 2015*	12 months-8 years	MF59-adjuvanted monovalent /Novartis/7.5 µg (n=145)	Non-adjuvanted monovalent /Novartis /15 µg (n=72)	Up to 18 months	14 subjects were reported, similarly distributed between adjuvanted and non-adjuvanted arms (the exact number of events in each arm was not specified).
Vesikari 2015	6-71 months (ATIV primed)	MF59-adjuvanted TIV /Novartis/30 µg (n=33)	Non-adjuvanted TIV /Novartis/30 µg (n=24)	Up to 6 months	1 subjects in adjuvanted arm, 0 subjects in non-adjuvanted arm.
Zedda 2015	6-35 months	MF59-adjuvanted /Novartis/30 µg (n=43)	Non-adjuvanted /Novartis/30 µg (n=39)	Did not report follow-up time for SAE	Did not report SAE data
Vesikari 2018†	6-60 months	MF59-adjuvanted, subunit quadrivalent, inactivated /Seqirus/30 µg (n=5,243)	Non-adjuvanted inactivated /Sanofi Pasteur/30 µg (n=5,161)	Up to 366 to 390 days	229 subjects in adjuvanted arm, 225 subjects in non-adjuvanted arm.

Study	Age of participants	Intervention /manufacturer/HA content per ml (n)	Comparator /manufacturer/HA content per ml (n)	Follow-up time	Results
Cruz-Valdez 2018	6-71 months	MF59-adjuvanted TIV /Novartis/30 µg (n=140)	Non-adjuvanted TIV /Novartis/30 µg (n=137)	Up to 50 days	0 subjects in adjuvanted arm, 0 subjects in non-adjuvanted arm.
Diallo 2018†	6-71 months	MF59-adjuvanted TIV /Novartis/30µg (n=118)	Non-adjuvanted TIV /Sanofi Pasteur/30 µg (n=119)	Up to 112 days	0 subjects in adjuvanted arm, 0 subjects in non-adjuvanted arm.

*This trial compared adjuvanted and non-adjuvanted vaccine formulations with unequal hemagglutinin antigen dose. The hemagglutinin antigen dose was reduced in the adjuvanted arm (antigen-sparing design).

†This trial compared adjuvanted influenza vaccines and non-adjuvanted influenza vaccines from different manufacturers.

Supplementary Table 4. Randomized controlled trials that reported neurological events

Study	Age of participants	Intervention /manufacturer/HA content per ml (n)	Comparator /manufacturer/HA content per ml (n)	Follow-up time	Results
Vesikari 2009 † (PIDJ)	6-35 months	MF59-adjuvanted subunit /Novartis/30 µg (n=130)	Non-adjuvanted split /Sanofi Pasteur/30 µg (n=139)	Up to 3 weeks	0 subjects in adjuvanted arm, 0 subjects in non-adjuvanted arm.
Vesikari 2009† (Vaccine)	16-47 months	MF59-adjuvanted subunit /Novartis/30 µg (n=43)	Non-adjuvanted split /Sanofi Pasteur/30 µg (n=46)	Up to 6 months	0 subjects in adjuvanted arm, 0 subjects in non-adjuvanted arm.
Waddington 2010*†	Waddington 2010	AS03-adjuvanted split virion /GSK/1.875 µg (n=270)	Non-adjuvanted whole virion /Baxter/3.75 µg (n=279)	Did not report follow-up time for SAE	0 subjects in adjuvanted arm, 0 subjects in non-adjuvanted arm.
Arguedas 2011*	3-8 years	MF59-adjuvanted /Novartis/15 µg (n=56)	Non-adjuvanted /Novartis/30 µg (n=54)	Up to 43 days	0 subjects in adjuvanted arm, 0 subjects in non-adjuvanted arm.
Della Cioppa 2011†	6-35 months	MF59-adjuvanted /Novartis/30 µg (n=37)	Non-adjuvanted /Sanofi Pasteur/30 µg (n=43)	Up to 50 days	0 subjects in adjuvanted arm, 0 subjects in non-adjuvanted arm.
Vesikari 2011	6-71 months	MF59-adjuvanted /Novartis/30 µg (n=2,012)	Non-adjuvanted subunit /Novartis/30 µg (n=1,846)	Up to 12 months	1 subjects (convulsion) in adjuvanted arm, 0 subjects in non-adjuvanted arm.

Study	Age of participants	Intervention /manufacturer/HA content per ml (n)	Comparator /manufacturer/HA content per ml (n)	Follow-up time	Results
Block 2012	6-35 months	MF59-adjuvanted monovalent subunit /Novartis/30 µg (n=161)	Non-adjuvanted monovalent subunit /Novartis/30 µg (n=161)	Up to 12 months	Did not report neurological event data.
Langley 2012*	6 months-8 years	AS03-adjuvanted monovalent /GSK/15 µg (n=59)	Non-adjuvanted monovalent /GSK/30 µg (n=57)	Up to 385 days	2 subjects (convulsion) in adjuvanted arm, 0 subjects in non-adjuvanted arm.
Nassim 2012	3-8 years	MF59-adjuvanted monovalent /Novartis/30 µg (n=334)	Non-adjuvanted monovalent /Novartis/30 µg (n=334)	Up to 387 days	Did not report neurological event data.
Nolan 2014†	6-71 months	MF59-adjuvanted /Novartis/30 µg (n=3,123)	Non-adjuvanted /Sanofi Pasteur/30 µg (n=1,474)	Up to 394 days	27 subjects were reported (the exact number of events in each arm was not specified).
Nolan 2014*	6-35 months	AS03-adjuvanted monovalent /GSK/7.5 µg (n=2,048)	Non-adjuvanted monovalent /GSK/30 µg (n=2,049)	Up to 385 days	1 subjects (febrile convulsion) in adjuvanted arm, 2 subjects (febrile convulsion) in non-adjuvanted arm.

Study	Age of participants	Intervention /manufacturer/HA content per ml (n)	Comparator /manufacturer/HA content per ml (n)	Follow-up time	Results
Solares 2014†	6-59 months	MF59-adjuvanted TIV /Novartis/30 µg (n=180)	Non-adjuvanted TIV /Sanofi Pasteur/30 µg (n=180)	Up to 211 days	0 subjects in adjuvanted arm, 0 subjects in non-adjuvanted arm.
Knuf 2015*	12 months-8 years	MF59-adjuvanted monovalent /Novartis/7.5 µg (n=145)	Non-adjuvanted monovalent /Novartis /15 µg (n=72)	Up to 18 months	5 subjects (3 multiple seizure, 2 febrile convulsion) in adjuvanted arm, 2 subjects (febrile convulsion) in non-adjuvanted group.
Vesikari 2015	6-71 months (ATIV primed)	MF59-adjuvanted TIV /Novartis/30 µg (n=33)	Non-adjuvanted TIV /Novartis/30 µg (n=24)	Up to 6 months	0 subjects in adjuvanted arm, 0 subjects in non-adjuvanted arm.
Zedda 2015	6-35 months	MF59-adjuvanted /Novartis/30 µg (n=43)	Non-adjuvanted /Novartis/30 µg (n=39)	Did not report follow-up time for SAE	Did not report neurological event data.
Vesikari 2018†	6-60 months	MF59-adjuvanted, subunit quadrivalent, inactivated /Seqirus/30 µg (n=5,243)	Non-adjuvanted inactivated /Sanofi Pasteur/30 µg (n=5,161)	Up to 366 to 390 days	0 subjects in adjuvanted arm, 0 subjects in non-adjuvanted arm.

Study	Age of participants	Intervention /manufacturer/HA content per ml (n)	Comparator /manufacturer/HA content per ml (n)	Follow-up time	Results
Cruz-Valdez 2018	6-71 months	MF59-adjuvanted TIV /Novartis/30 µg (n=140)	Non-adjuvanted TIV /Novartis/30 µg (n=137)	Up to 50 days	0 subjects in adjuvanted arm, 0 subjects in non-adjuvanted arm.
Diallo 2018†	6-71 months	MF59-adjuvanted TIV /Novartis/30µg (n=118)	Non-adjuvanted TIV /Sanofi Pasteur/30 µg (n=119)	Up to 112 days	0 subjects in adjuvanted arm, 0 subjects in non-adjuvanted arm.

*This trial compared adjuvanted and non-adjuvanted vaccine formulations with unequal hemagglutinin antigen dose. The hemagglutinin antigen dose was reduced in the adjuvanted arm (antigen-sparing design).

†This trial compared adjuvanted influenza vaccines and non-adjuvanted influenza vaccines from different manufacturers.

Supplementary Table 5. Randomized controlled trials that reported reactogenicity data

Study	Study design	Setting	Vaccine strain	Age of participants	Intervention /manufacturer/HA content per ml (n)	Comparator /manufacturer/ HA content per ml (n)	Time point	Symptoms
Vesikari 2009† (PIDJ)	Observer-blind Randomized	Finland	H1N1, H3N2, B	6-35 months	MF59-adjuvanted subunit /Novartis/30 µg (n=130)	Non-adjuvanted split /Sanofi Pasteur/30 µg (n=139)	after 1st and 2nd doses	Pain Fever
Vesikari 2009† (Vaccine)	Observer-blind Randomized	Finland	H1N1, H3N2, B	16-47 months	MF59-adjuvanted subunit /Novartis/30 µg (n=43)	Non-adjuvanted split /Sanofi Pasteur/30 µg (n=46)	after 1st dose	Fever
Waddington 2010*† (Funded by UK NIHR)	Phase 2 Open-label Randomized	5 sites in UK	H1N1	6-59 months	AS03-adjuvanted split virion /GSK/1.875 µg (n=270)	Non-adjuvanted whole virion /Baxter/3.75 µg (n=279)	after 1st and 2nd doses	Pain Fever
Arguedas 2011*	Phase 3 Open-label Randomized	Costa Rica	H1N1	3-8 years	MF59-adjuvanted /Novartis/15 µg (n=56)	Non-adjuvanted /Novartis/30 µg (n=54)	after 1st and 2nd doses	Pain

Study	Study design	Setting	Vaccine strain	Age of participants	Intervention /manufacturer/HA content per ml (n)	Comparator /manufacturer/HA content per ml (n)	Time point	Symptoms
Della Cioppa 2011†	Observer-blind Randomized	15 study centers in Finland and Belgium	H1N1, H3N2, B	6-35 months	MF59-adjuvanted /Novartis/30 µg (n=37)	Non-adjuvanted /Sanofi Pasteur/30 µg (n=43)	after 1st and 2nd doses	Reported pool solicited data by local and systemic reactions
Vesikari 2011	Phase 3 Observer-blind Randomized	Germany, Finland	H1N1, H3N2, B	6-35 months	MF59-adjuvanted /Novartis/30 µg (n=1100)	Non-adjuvanted subunit /Novartis/30 µg (n=992)	after 1st and 2nd doses	Fever
				36-71 months	MF59-adjuvanted /Novartis/30 µg (n=833)	Non-adjuvanted subunit /Novartis/30 µg (n=777)	after 1st and 2nd doses	Pain Fever
Block 2012	Phase 2 Phase 3 Double-blind Randomized	29 sites in USA and Mexico	H1N1	6-35 months	MF59-adjuvanted monovalent subunit /Novartis/30 µg (n=161)	Non-adjuvanted monovalent subunit /Novartis/30 µg (n=161)	after 1st and 2nd doses	Pain Fever

Study	Study design	Setting	Vaccine strain	Age of participants	Intervention /manufacturer/HA content per ml (n)	Comparator /manufacturer/ HA content per ml (n)	Time point	Symptoms
Langley 2012*	Double-blind Randomized	12 centers in Canada	H1N1	6 months-8 years	AS03-adjuvanted monovalent /GSK /15 µg (n=59)	Non-adjuvanted monovalent /GSK/30 µg (n=57)	7 d after 1st and 2nd doses	Pain Fever (did not report case number or percentage for adjuvanted and non-adjuvanted groups respectively)
Nassim 2012	Phase 2 phase 3 Single-blind Randomized	34 centers in USA	H1N1	3-8 years (antigen 7.5µg)	MF59-adjuvanted monovalent /Novartis/30 µg (n=168)	Non-adjuvanted monovalent /Novartis/30 µg (n=167)	after 1st and 2nd doses	Pain Fever
				3-8 years (antigen 15 µg)	MF59-adjuvanted monovalent /Novartis/30 µg (n=166)	Non-adjuvanted monovalent /Novartis/30 µg (n=167)	after 1st and 2nd doses	Pain Fever
Nolan 2014†	Phase 3 Observer-blind Randomized	32 sites in Argentina, Australia, Chile, Philippines, South Africa	H1N1, H3N2, B	6-71 months	MF59-adjuvanted /Novartis/30 µg (n=3,074)	Non-adjuvanted /Sanofi Pasteur/30 µg (n=1,443)	after 1st and 2nd doses	Pain Fever

Study	Study design	Setting	Vaccine strain	Age of participants	Intervention /manufacturer/HA content per ml (n)	Comparator /manufacturer/HA content per ml (n)	Time point	Symptoms
Nolan 2014*	Phase 3<br>Observer-blind<br>Randomized	17 centers in Australia, Brazil, Colombia, Costa Rica, Mexico, Philippines, Singapore, Thailand	H1N1	6-35 months	AS03-adjuvanted monovalent /GSK/7.5 µg (n=2,048)	Non-adjuvanted monovalent /GSK/30 µg (n=2,049)	after 1st and 2nd doses	Pain<br>Fever
Solares 2014†	phase 2<br>Observer-blind<br>Randomized	5 sites in Guatemala	H1N1, H3N2, B	6-35 months	MF59-adjuvanted TIV /Novartis/30 µg (n=130)	Non-adjuvanted TIV /Sanofi Pasteur/30 µg (n=130)	after 1st and 2nd doses	Pain<br>Fever
				36-59 months	MF59-adjuvanted TIV /Novartis/30 µg (n=50)	Non-adjuvanted TIV /Sanofi Pasteur/30 µg (n=50)	after 1st and 2nd doses	Pain<br>Fever
Knuf 2015*	Single-blind<br>Randomized	13 centers in Germany, Belgium, Dominican Republic, Netherlands	H1N1	12-35 months	MF59-adjuvanted monovalent /Novartis/7.5 µg (n=73)	Non-adjuvanted monovalent /Novartis/15 µg (n=33)	after 1st and 2nd doses	Pain<br>Fever

Study	Study design	Setting	Vaccine strain	Age of participants	Intervention /manufacturer/HA content per ml (n)	Comparator /manufacturer/ HA content per ml (n)	Time point	Symptoms
				3-8 years	MF59-adjuvanted monovalent /Novartis/7.5 μg (n=72)	Non-adjuvanted monovalent /Novartis/15 μg (n=39)	after 1st and 2nd doses	Pain Fever
Vesikari 2015	Phase 3b Observer-blind Randomized	15 sites in Finland	H1N1, H3N2, B	6-71 months (ATIV primed)	MF59-adjuvanted TIV /Novartis/30 μg (n=12)	Non-adjuvanted TIV /Novartis/30 μg (n=10)	after 1st dose	Pain Fever
				6-71 months (TIV primed)	MF59-adjuvanted TIV /Novartis/30 μg (n=21)	Non-adjuvanted TIV /Novartis/30 μg (n=14)	after 1st dose	Pain Fever
Zedda 2015	Randomized	Belgium	H1N1, H3N2, B	6-35 months	MF59-adjuvanted /Novartis/30 μg (n=43)	Non-adjuvanted /Novartis/30 μg (n=39)	after 1st and 2nd doses	Pain Fever
Vesikari 2018†	Phase 3 Observer-blind, Randomized	146 sites including hospitals, clinics and clinician offices in Finland, USA, Canada, Italy, Poland, Spain, Philippines, Thailand, Taiwan	H1N1, H3N2, B	6-60 months	MF59-adjuvanted, subunit quadrivalent, inactivated /Seqirus/30 μg (n=5,138)	Non-adjuvanted, split virion, inactivated /Sanofi Pasteur/30 μg (n=5,056)	Pooled data of after 1st and 2nd doses	Pain Fever

Study	Study design	Setting	Vaccine strain	Age of participants	Intervention /manufacturer/HA content per ml (n)	Comparator /manufacturer/ HA content per ml (n)	Time point	Symptoms
Diallo 2018†	Observer-blind randomized	Senegal	H1N1, H3N2, B	6-35 months	MF59-adjuvanted TIV /Novartis/30 µg (n=78)	Non-adjuvanted /Sanofi Pasteur/30 µg TIV (n=79)	after 1st and 2nd doses	Pain Fever
				36-71 months	MF59-adjuvanted TIV /Novartis/30 µg (n=40)	Non-adjuvanted TIV /Sanofi Pasteur/30 µg (n=40)	after 1st and 2nd doses	Pain Fever
Cruz-Valdez 2018	Phase 3 Observer-blind randomized	Mexico	H1N1, H3N2, B	6-71 months	MF59-adjuvanted TIV /Novartis/30 µg (n=140)	Non-adjuvanted TIV /Novartis/30 µg (n=137)	after 1st and 2nd doses	Reported pool solicited data by local and systemic reactions

*This trial compared adjuvanted and non-adjuvanted vaccine formulations with unequal hemagglutinin antigen dose. The hemagglutinin antigen dose was reduced in the adjuvanted arm (antigen-sparing design).

†This trial compared adjuvanted influenza vaccines and non-adjuvanted influenza vaccines from different manufacturers.

Supplementary Table 6. Summary of findings: certainty of evidence for effect outcomes

Summary of findings:

Oil-in-water emulsion adjuvanted influenza vaccine compared to non adjuvanted influenza vaccine in children

Patient or population: children
Setting: Poor response (a <25% seroprotection after a single dose of nonadjuvanted influenza vaccine)
Intervention: oil-in-water emulsion adjuvanted influenza vaccine
Comparison: non adjuvanted influenza vaccine

Outcomes	**Anticipated absolute effects*** (95% CI)		Relative effect (95% CI)	№ of participants (studies)	Certainty of the evidence (GRADE)	Comments
	**Risk with non adjuvanted influenza vaccine**	**Risk with oil-in-water emulsion adjuvanted influenza vaccine**				
vaccine efficacy assessed with RT-PCR test	22 per 1,000	**6 per 1,000** (3 to 10)	**RR 0.26** (0.14 to 0.47)	4839 (3 RCTs)	⨁⨁⨁⨁ HIGH [a,b]	
seroprotection assessed with: HI titer for H1N1 influenza virus after the first dose follow up: range 21 days to 28 days	195 per 1,000	**897 per 1,000** (800 to 1,015)	**Rate ratio 4.6** (4.1 to 5.2)	2308 (4 RCTs)	⨁⨁⨁◯ MODERATE [c,d,e,f]	
seroprotection assessed with: HI titer for H3N2 influenza virus after the first dose follow up: range 21 days to 28 days	119 per 1,000	**944 per 1,000** (800 to 1,123)	**Rate ratio 7.9** (6.7 to 9.4)	2178 (2 RCTs)	⨁⨁⨁◯ MODERATE [d,e,f,g]	
seroprotection assessed with: HI titer for influenza B after the first dose follow up: range 21 days to 28 days	120 per 1,000	**168 per 1,000** (96 to 276)	**Rate ratio 1.4** (0.8 to 2.3)	2377 (3 RCTs)	⨁◯◯◯ VERY LOW [e,g,h,i,j]	
seroprotection assessed with: Hi titer for influenza B after the second dose follow up: range 21 days to 28 days	187 per 1,000	**1010 per 1,000** (711 to 1,403)	**Rate ratio 5.4** (3.8 to 7.5)	2176 (2 RCTs)	⨁⨁⨁◯ MODERATE [d,e,f,k]	

***The risk in the intervention group** (and its 95% confidence interval) is based on the assumed risk in the comparison group and the **relative effect** of the intervention (and its 95% CI).
CI: Confidence interval; **RR:** Risk ratio

GRADE Working Group grades of evidence
High certainty: We are very confident that the true effect lies close to that of the estimate of the effect
Moderate certainty: We are moderately confident in the effect estimate: The true effect is likely to be close to the estimate of the effect, but there is a possibility that it is substantially different
Low certainty: Our confidence in the effect estimate is limited: The true effect may be substantially different from the estimate of the effect
Very low certainty: We have very little confidence in the effect estimate: The true effect is likely to be substantially different from the estimate of effect

Explanations

a. Only 3 of the 18 included trials reported efficacy data.
b. RR<0.5
c. Only 21 of the 26 included comparisons reported seroprotection rate data after the first-dose vaccination.
d. The rate ratio was for poor response group. Meta-regression shows that between-trials heterogeneity in rate ratio was nearly all explained by serological response rates in the non-adjuvanted arm (see text for details).
e. Measurement of immunogenicity was used as a surrogate for real-life protection against infection, disease and death
f. RR >2.0
g. Only 12 of the 26 included comparisons reported seroprotection rate data after the first-dose vaccination.
h. Test for heterogeneity p=0.05, I-square 67%
i. The rate ratio was for poor response group
j. Wide confidence interval
k. Only 13 of the 26 included comparisons reported seroprotection rate data after the second-dose vaccination.

Supplementary Table 7. Summary of findings: certainty of evidence for harm outcomes

Summary of findings:

Oil-in-water emulsion adjuvanted influenza vaccine compared to non adjuvanted influenza vaccine in children

Patient or population: children
Setting:
Intervention: oil-in-water emulsion adjuvanted influenza vaccine
Comparison: non adjuvanted influenza vaccine

Outcomes	**Anticipated absolute effects*** (95% CI)		Relative effect (95% CI)	№ of participants (studies)	Certainty of the evidence (GRADE)	Comments
	**Risk with non adjuvanted influenza vaccine**	**Risk with oil-in-water emulsion adjuvanted influenza vaccine**				
Severe adverse events	47 per 1,000	**42 per 1,000** (33 to 52)	**RR 0.9** (0.7 to 1.1)	25541 (15 RCTs)	⨁◯◯◯ VERY LOW [a,b,c]	
Neurological events	0 per 1,000	**1 per 1,000** (0 to 2)	**RR 1.4** (0.4 to 4.3)	20802 (15 RCTs)	⨁⨁◯◯ LOW [d,e]	
pain at the injection site after the first dose assessed with: yes or no	326 per 1,000	**424 per 1,000** (391 to 489)	**RR 1.3** (1.2 to 1.5)	23184 (14 RCTs)	⨁⨁◯◯ LOW [f,g]	
pain at the injection site after the second dose assessed with: yes or no	199 per 1,000	**299 per 1,000** (239 to 359)	**RR 1.5** (1.2 to 1.8)	12791 (13 RCTs)	⨁⨁◯◯ LOW [h,i]	
fever after the first dose assessed with: body temperature higher than 38 Celsius degree	93 per 1,000	**149 per 1,000** (130 to 167)	**RR 1.6** (1.4 to 1.8)	25359 (14 RCTs)	⨁⨁◯◯ LOW [j,k]	
fever after the second dose assessed with: body temperature higher than 38 celsius degree	89 per 1,000	**134 per 1,000** (107 to 169)	**RR 1.5** (1.2 to 1.9)	14719 (12 RCTs)	⨁⨁◯◯ LOW [l,m]	

***The risk in the intervention group** (and its 95% confidence interval) is based on the assumed risk in the comparison group and the **relative effect** of the intervention (and its 95% CI).
CI: Confidence interval; **RR:** Risk ratio

GRADE Working Group grades of evidence
High certainty: We are very confident that the true effect lies close to that of the estimate of the effect
Moderate certainty: We are moderately confident in the effect estimate: The true effect is likely to be close to the estimate of the effect, but there is a possibility that it is substantially different
Low certainty: Our confidence in the effect estimate is limited: The true effect may be substantially different from the estimate of the effect
Very low certainty: We have very little confidence in the effect estimate: The true effect is likely to be substantially different from the estimate of effect

Explanations

a. Only 15 of the 18 included trials reported rate data of SAEs during the follow-up period
b. Test for heterogeneity P=0.02, I-square 50%
c. Confidence interval 0.69-1.12
d. Only 15 of the 18 included trials reported rate data of neurological events during the follow-up period
e. Wide confidence interval
f. Only 19 of the 21 included comparisons reported rate data of pain after the first-dose vaccination
g. Test for heterogeneity P<0.01, I-square 76%
h. Only 17 of the 21 included comparisons reported rate data of pain after the second-dose vaccination
i. Test for heterogeneity P<0.01, I-square 81%
j. Only 20 of the 21 included comparisons reported rate data of fever after the first-dose vaccination
k. Test for heterogeneity P=0.01, I-square 46%
l. Only 17 of the 21 included comparisons reported rate data of fever after the second-dose vaccination
m. Test for heterogeneity P<0.01, I-square 65%

Supplementary Figure 1. Forest plot showing the ratios of the seroprotection rate (SPR) against H1N1, defined as a ≥1:40 haemagglutinin-inhibition (HI) titer, 21–28 days after the second dose among children who received adjuvanted versus non-adjuvanted trivalent vaccines (TIV) or quadrivalent vaccines (QIV).

Study or Subgroup	adjuvanted Events	adjuvanted Total	Nonadjuvanted Events	Nonadjuvanted Total	Weight	Seroprotection Rate ratio M-H, Fixed, 95% CI	Year
**4.2.1 Moderate response to non-adjuvanted TIV/QIV (SPR 25% to 75%)**							
Vesikari 2011 (6-35 months)	1103	1103	373	995	34.3%	2.67 [2.46, 2.89]	2011
Block 2012 (6-35 months)	127	127	87	124	7.7%	1.42 [1.27, 1.60]	2012
Langley 2012 (6-11 months)	3	3	2	3	0.2%	1.40 [0.60, 3.26]	2012
Vesikari 2018 (6-60 months)	705	922	622	866	56.0%	1.06 [1.01, 1.12]	2018
Diallo 2018 (6-11 months)	30	30	19	26	1.8%	1.36 [1.07, 1.73]	2018
**Subtotal (95% CI)**		**2185**		**2014**	**100.0%**	**1.65 [1.58, 1.72]**	
Total events	1968		1103				
Heterogeneity: Chi² = 389.22, df = 4 (P < 0.00001); I² = 99%							
Test for overall effect: Z = 22.31 (P < 0.00001)							
**4.2.2 Good response to non-adjuvanted TIV/QIV (SPR >75%)**							
Vesikari 2009 (6-35 months, PIDJ)	104	104	101	118	4.8%	1.17 [1.08, 1.26]	2009
Della Cioppa 2011 (6-11 months)	36	36	33	42	1.5%	1.27 [1.08, 1.49]	2011
Arguedas 2011 (3-8 y)	45	46	68	75	2.6%	1.08 [0.99, 1.17]	2011
Vesikari 2011 (36-71 months)	834	834	641	777	33.2%	1.21 [1.17, 1.25]	2011
Langley 2012 (3-8 years)	28	28	29	30	1.4%	1.03 [0.94, 1.13]	2012
Nassim 2012 (3-8 years, 7.5 μg)	151	156	120	156	6.0%	1.26 [1.15, 1.38]	2012
Langley 2012 (12-35 mo)	13	13	10	11	0.6%	1.10 [0.87, 1.40]	2012
Nassim 2012 (3-8 years, 15 μg)	154	156	130	155	6.5%	1.18 [1.10, 1.26]	2012
Solares 2014 (6-35 months)	97	97	100	102	4.9%	1.02 [0.99, 1.05]	2014
Nolan 2014 (6-35 months, AS03 adjuvanted)	233	233	199	223	10.2%	1.12 [1.07, 1.17]	2014
Solares 2014 (36-59 months)	22	23	20	20	1.1%	0.96 [0.85, 1.09]	2014
Nolan 2014 (6-71 months, MF59 adjuvanted)	266	266	338	389	13.8%	1.15 [1.11, 1.20]	2014
Knuf 2015 (3-8 years)	60	60	30	31	2.0%	1.04 [0.96, 1.13]	2015
Knuf 2015 (12-35 months)	53	53	22	25	1.5%	1.14 [0.98, 1.34]	2015
Zedda 2015 (6-35 months)	25	25	28	30	1.3%	1.07 [0.95, 1.20]	2015
Diallo 2018 (12-35 months)	31	31	32	34	1.6%	1.06 [0.96, 1.17]	2018
Diallo 2018 (36-71 months)	38	38	32	33	1.7%	1.03 [0.95, 1.12]	2018
Cruz-Valdez 2018 (6-71 months)	113	114	105	112	5.3%	1.06 [1.00, 1.11]	2018
**Subtotal (95% CI)**		**2313**		**2363**	**100.0%**	**1.15 [1.14, 1.17]**	
Total events	2303		2038				
Heterogeneity: Chi² = 114.38, df = 17 (P < 0.00001); I² = 85%							
Test for overall effect: Z = 16.86 (P < 0.00001)							

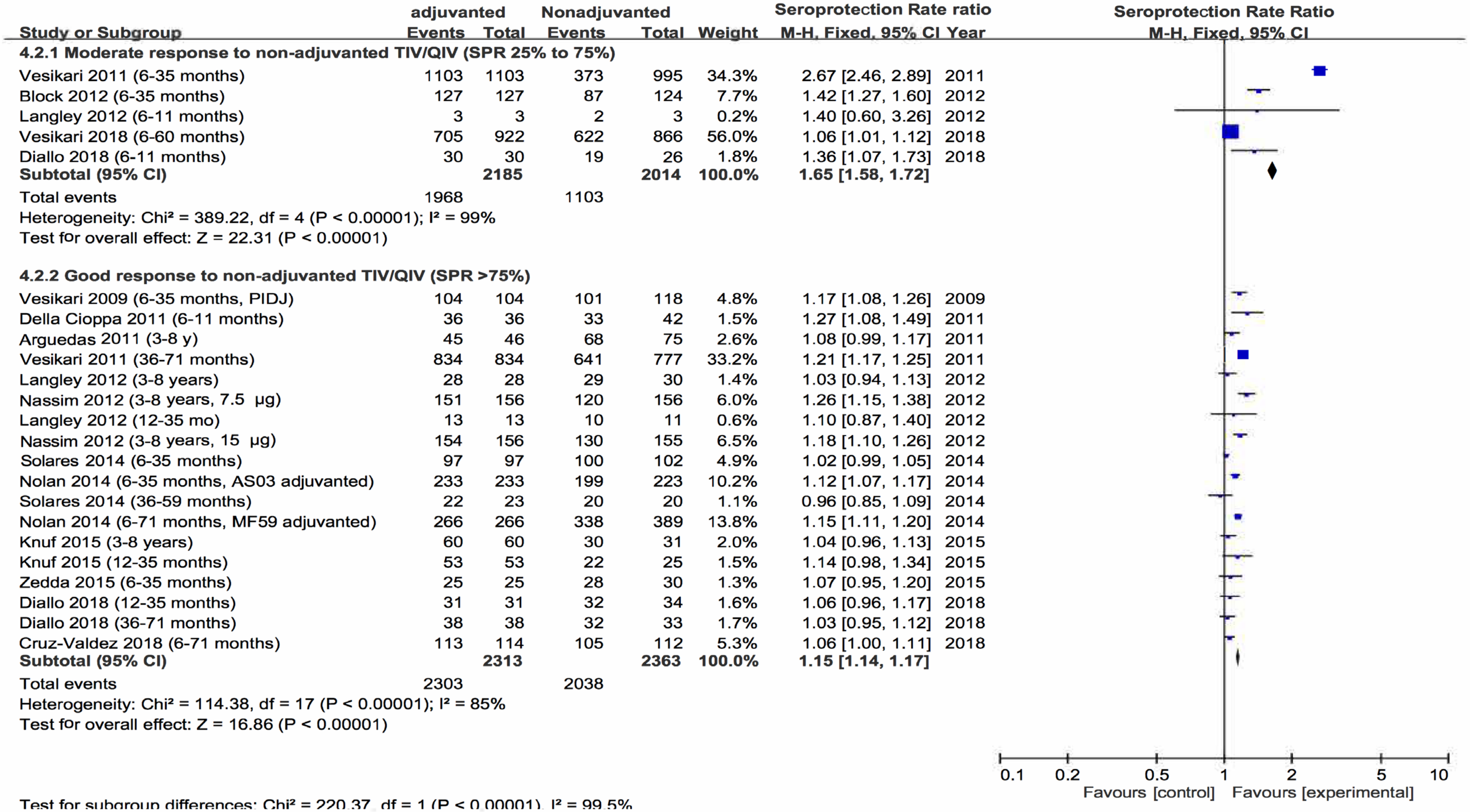

Test for subgroup differences: Chi² = 220.37, df = 1 (P < 0.00001), I² = 99.5%

Supplementary Figure 2. Meta-regression showing a linear relationship (slope = 0.98, P <0.001) between the ratios of the seroprotection rate against H1N1 after the second dose and the inverse of seroprotection rate in non-adjuvanted arm, with an R^2 of 95.47%

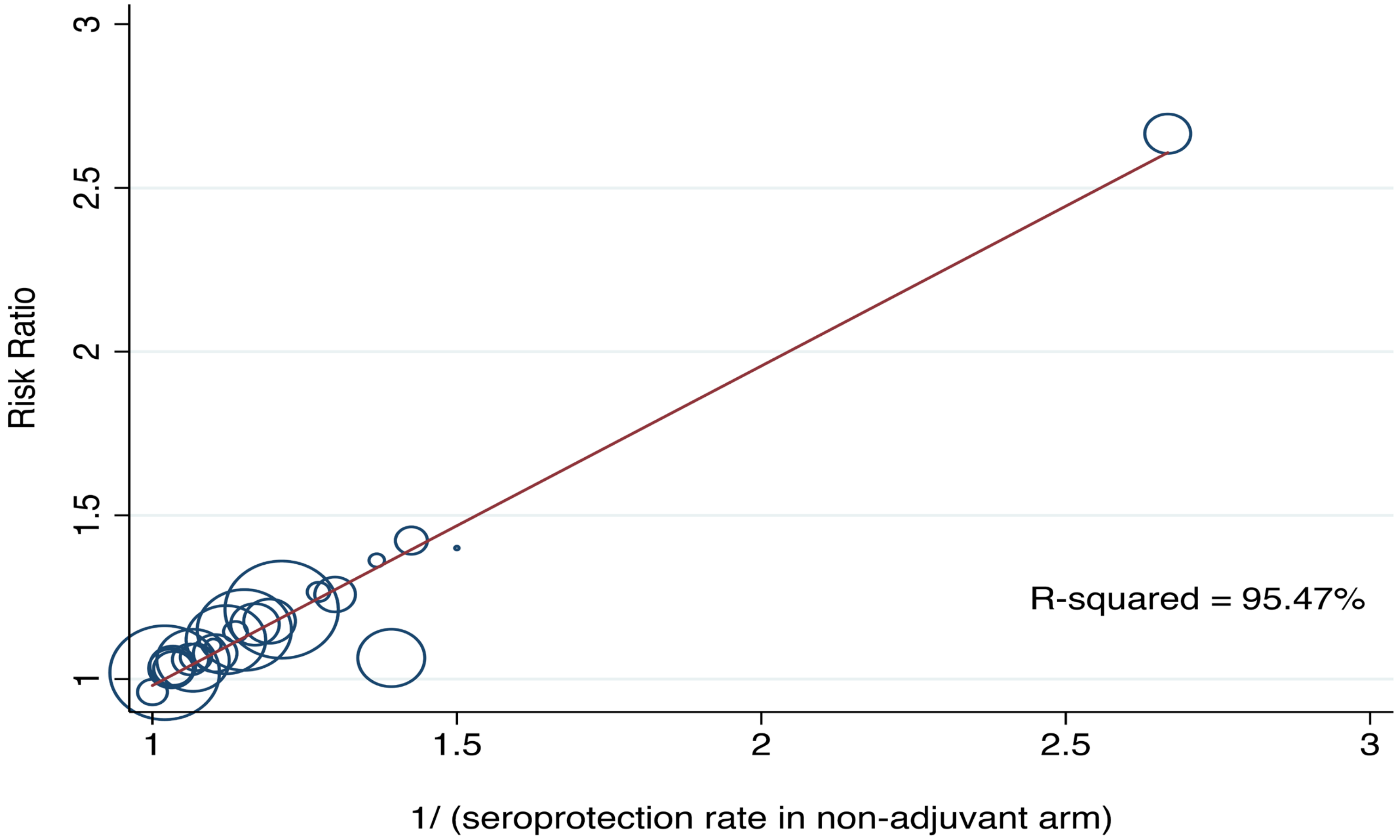

Supplementary Figure 3. Forest plot showing the ratios of the seroprotection rate (SPR) against H3N2, defined as a ≥1:40 haemagglutinin-inhibition (HI) titer, 21–28 days after the second dose among children who received adjuvanted versus non-adjuvanted trivalent vaccines (TIV) or quadrivalent vaccines (QIV).

Study or Subgroup	adjuvanted Events	adjuvanted Total	Nonadjuvanted Events	Nonadjuvanted Total	Weight	Seroprotection Rate Ratio M-H, Random, 95% CI	Year
**4.4.1 Moderate response to non-adjuvanted TIV/QIV (SPR 25% to 75%)**							
Della Cioppa 2011 (6-11 months)	36	36	30	42	32.6%	1.39 [1.14, 1.69]	2011
Vesikari 2011 (6-35 months)	1075	1103	448	995	33.7%	2.16 [2.02, 2.32]	2011
Vesikari 2018 (6-60 months)	669	922	619	866	33.7%	1.02 [0.96, 1.08]	2018
**Subtotal (95% CI)**		**2061**		**1903**	**100.0%**	**1.45 [0.81, 2.59]**	
Total events	1780		1097				
Heterogeneity: Tau² = 0.26; Chi² = 283.26, df = 2 (P < 0.00001); I² = 99%							
Test for overall effect: Z = 1.26 (P = 0.21)							
**4.4.2 Good response to non-adjuvanted TIV/QIV (SPR >75%)**							
Vesikari 2009 (6-35 months, PIDJ)	104	104	117	118	11.6%	1.01 [0.98, 1.03]	2009
Vesikari 2011 (36-71 months)	817	834	680	777	11.5%	1.12 [1.09, 1.15]	2011
Solares 2014 (6-35 months)	97	97	93	102	9.8%	1.10 [1.03, 1.17]	2014
Solares 2014 (36-59 months)	23	23	20	20	8.3%	1.00 [0.92, 1.09]	2014
Nolan 2014 (6-71 months, MF59 adjuvanted)	266	266	385	389	11.9%	1.01 [1.00, 1.02]	2014
Zedda 2015 (6-35 months)	25	25	29	30	7.9%	1.03 [0.94, 1.13]	2015
Diallo 2018 (12-35 months)	31	31	34	34	10.0%	1.00 [0.94, 1.06]	2018
Cruz-Valdez 2018 (6-71 months)	114	114	108	112	11.0%	1.04 [1.00, 1.08]	2018
Diallo 2018 (6-11 months)	30	30	25	26	7.6%	1.04 [0.94, 1.15]	2018
Diallo 2018 (36-71 months)	38	38	33	33	10.3%	1.00 [0.95, 1.06]	2018
**Subtotal (95% CI)**		**1562**		**1641**	**100.0%**	**1.03 [0.99, 1.08]**	
Total events	1545		1524				
Heterogeneity: Tau² = 0.00; Chi² = 139.97, df = 9 (P < 0.00001); I² = 94%							
Test for overall effect: Z = 1.41 (P = 0.16)							

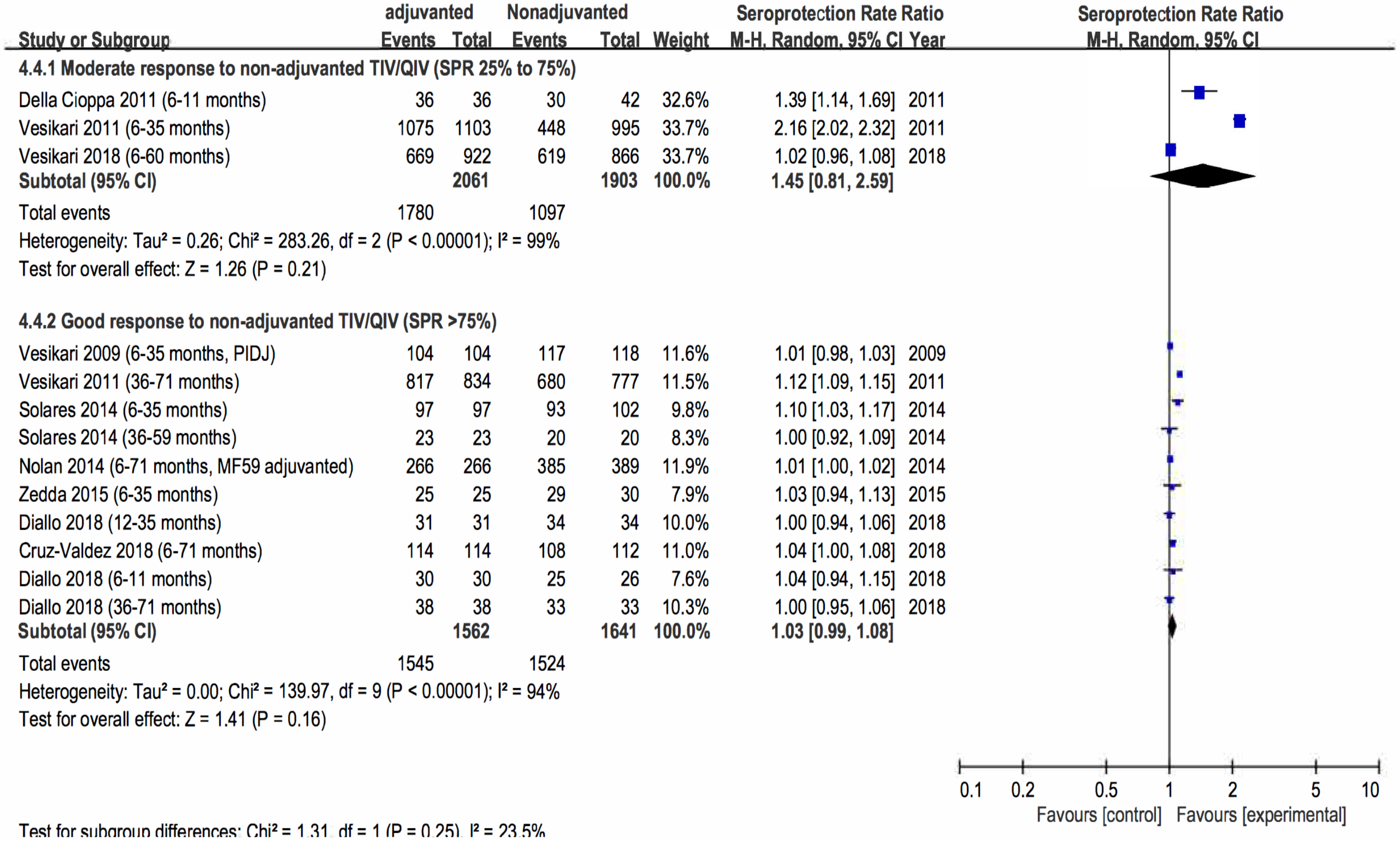

Test for subgroup differences: Chi² = 1.31, df = 1 (P = 0.25), I² = 23.5%

Supplementary Figure 4. Meta-regression showing a linear relationship (slope = 0.91, P <0.001) between the ratios of seroprotection rates against H3N2 after the second dose and the inverse of seroprotection rate in non-adjuvanted arm, with an R^2 of 88.92%

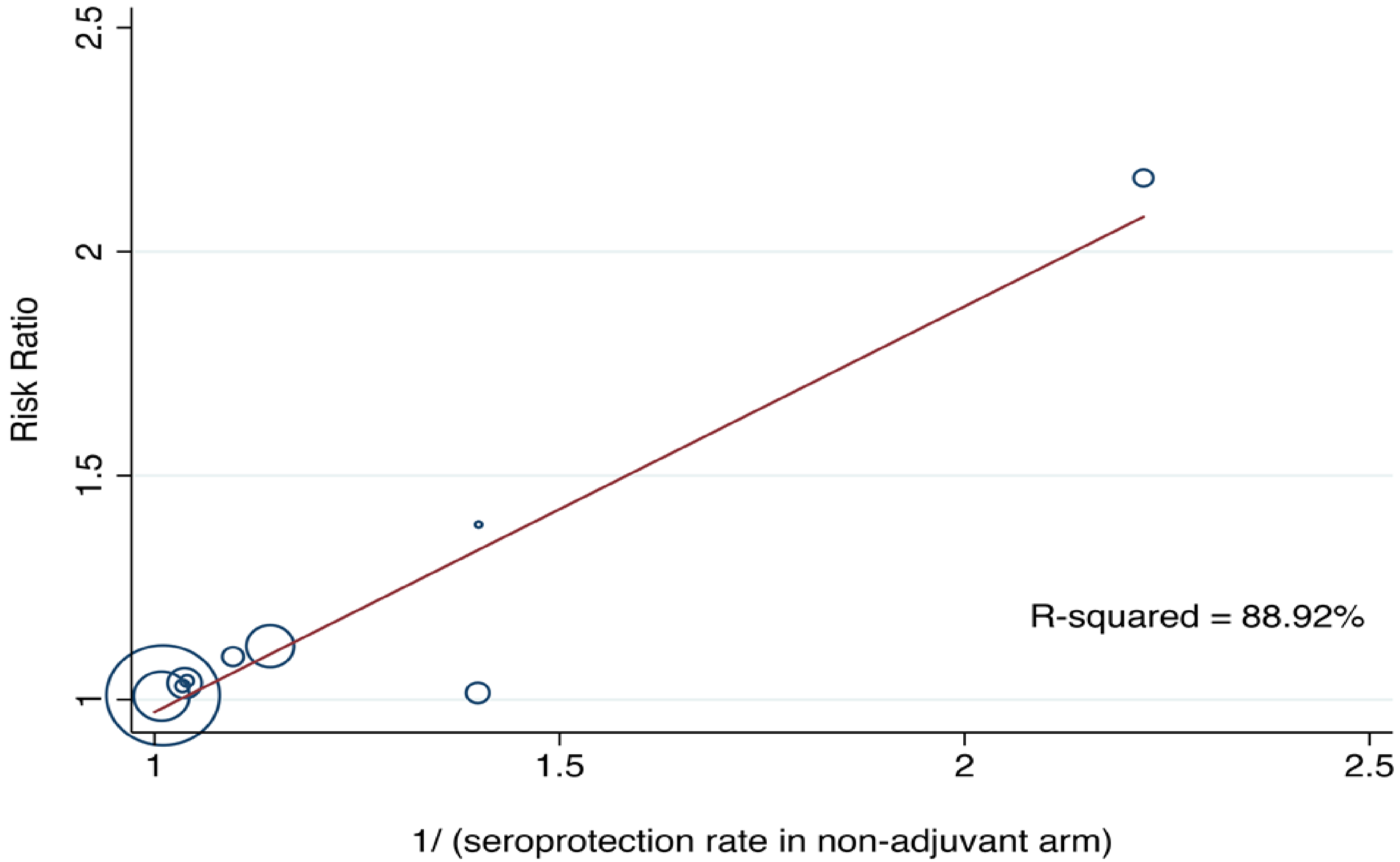

Supplementary Figure 5. Forest plot showing the ratios of the seroprotection rate (SPR) against influenza B, defined as a ≥1:40 haemagglutinin-inhibition HI) titer, 21–28 days after the first dose among children who received adjuvanted versus non-adjuvanted trivalent vaccines (TIV) or quadrivalent vaccines (QIV).

Study or Subgroup	adjuvanted Events	adjuvanted Total	Nonadjuvanted Events	Nonadjuvanted Total	Weight	Seroprotection Rate Ratio M-H, Random, 95% CI	Year
**4.5.1 Poor response to non-adjuvanted TIV/QIV (SPR <25%)**							
Vesikari 2011 (6-35 months)	138	1103	124	995	49.5%	1.00 [0.80, 1.26]	2011
Della Cioppa 2011 (6-11 months)	8	37	3	43	12.9%	3.10 [0.89, 10.84]	2011
Solares 2014 (6-35 months)	33	97	21	102	37.6%	1.65 [1.03, 2.65]	2014
**Subtotal (95% CI)**		**1237**		**1140**	**100.0%**	**1.40 [0.84, 2.34]**	
Total events	179		148				
Heterogeneity: Tau² = 0.13; Chi² = 6.04, df = 2 (P = 0.05); I² = 67%							
Test for overall effect: Z = 1.28 (P = 0.20)							
**4.5.2 Moderate response to non-adjuvanted TIV/QIV (SPR 25% to 75%)**							
Vesikari 2009 (16-47 months, Vaccine)	41	41	27	40	16.0%	1.47 [1.19, 1.83]	2009
Vesikari 2011 (36-71 months)	375	834	311	777	22.3%	1.12 [1.00, 1.26]	2011
Solares 2014 (36-59 months)	12	23	9	20	4.2%	1.16 [0.62, 2.16]	2014
Vesikari 2015 (6-71 months, ATIV primed)	15	17	8	12	7.4%	1.32 [0.86, 2.05]	2015
Vesikari 2015 (6-71 months, TIV primed)	13	23	4	14	2.2%	1.98 [0.80, 4.88]	2015
Diallo 2018 (36-71 months)	29	38	22	33	11.9%	1.14 [0.85, 1.54]	2018
Diallo 2018 (6-11 months)	19	30	11	26	5.6%	1.50 [0.89, 2.53]	2018
Vesikari 2018 (6-60 months)	455	922	275	866	22.1%	1.55 [1.38, 1.75]	2018
Diallo 2018 (12-35 months)	20	31	19	34	8.4%	1.15 [0.78, 1.72]	2018
**Subtotal (95% CI)**		**1959**		**1822**	**100.0%**	**1.32 [1.15, 1.52]**	
Total events	979		686				
Heterogeneity: Tau² = 0.02; Chi² = 18.54, df = 8 (P = 0.02); I² = 57%							
Test for overall effect: Z = 3.90 (P < 0.0001)							

Seroprotection Rate Ratio
M-H, Random, 95% CI

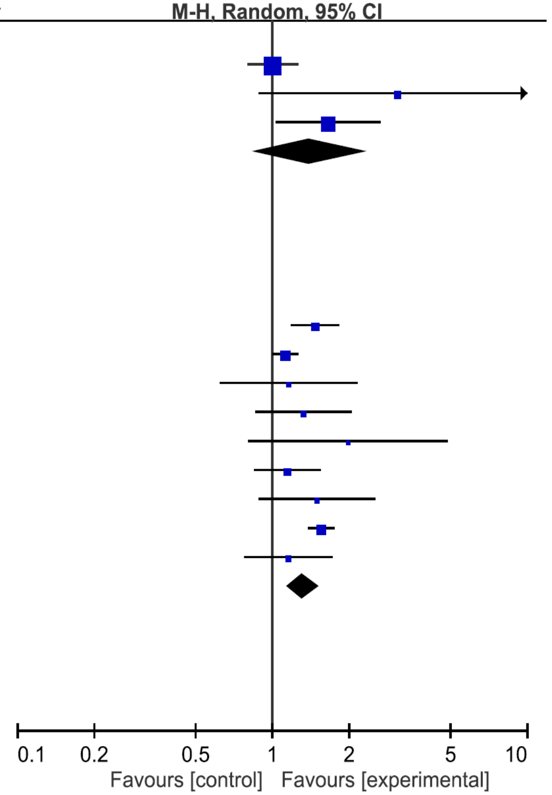

Test for subgroup differences: Chi² = 0.05, df = 1 (P = 0.83), I² = 0%

Supplementary Figure 6. Forest plot showing the risk ratios of pain/tenderness at the injection site during the 7 days after the first dose among children who received adjuvanted versus non-adjuvanted trivalent vaccines (TIV) or quadrivalent vaccines (QIV).

	adjuvanted		non-adjuvanted			Risk Ratio
Study or Subgroup	Events	Total	Events	Total	Weight	M-H, Random, 95% CI
Arguedas 2011 (3-8 y)	21	55	17	54	3.0%	1.21 [0.72, 2.04]
Block 2012 (6-35 months)	53	161	42	161	5.1%	1.26 [0.90, 1.77]
Diallo 2018 (36-71 months)	13	40	9	40	1.7%	1.44 [0.70, 2.99]
Diallo 2018 (6-35 months)	32	78	28	79	4.2%	1.16 [0.78, 1.73]
Knuf 2015 (12-35 months)	30	73	10	33	2.5%	1.36 [0.75, 2.44]
Knuf 2015 (3-8 years)	33	72	14	39	3.2%	1.28 [0.78, 2.08]
Nassim 2012 (3-8 years, 15 µg)	72	166	47	167	5.8%	1.54 [1.14, 2.08]
Nassim 2012 (3-8 years, 7.5 µg)	86	168	43	167	5.9%	1.99 [1.48, 2.68]
Nolan 2014 (6-35 months, AS03 adjuvanted)	881	2048	861	2049	10.6%	1.02 [0.95, 1.10]
Nolan 2014 (6-71 months, MF59 adjuvanted)	987	2991	284	1422	9.8%	1.65 [1.47, 1.86]
Solares 2014 (36-59 months)	22	50	14	50	2.8%	1.57 [0.91, 2.71]
Solares 2014 (6-35 months)	23	130	20	130	2.7%	1.15 [0.67, 1.99]
Vesikari 2009 (6-35 months, PIDJ)	43	130	36	139	4.6%	1.28 [0.88, 1.85]
Vesikari 2011 (36-71 months)	377	833	274	777	9.7%	1.28 [1.14, 1.45]
Vesikari 2015 (6-71 months, ATIV primed)	11	12	9	10	6.4%	1.02 [0.78, 1.33]
Vesikari 2015 (6-71 months, TIV primed)	15	21	10	14	3.9%	1.00 [0.65, 1.53]
Vesikari 2018 (6-60 months)	2218	5138	1711	5056	10.9%	1.28 [1.21, 1.34]
Waddington 2010 (6-59 months)	85	270	51	279	5.7%	1.72 [1.27, 2.33]
Zedda 2015 (6-35 months)	10	43	9	39	1.5%	1.01 [0.46, 2.22]
**Total (95% CI)**		**12479**		**10705**	**100.0%**	**1.31 [1.18, 1.46]**
Total events	5012		3489			

Heterogeneity: Tau² = 0.03; Chi² = 74.73, df = 18 (P < 0.00001); I² = 76%

Test for overall effect: Z = 5.15 (P < 0.00001)

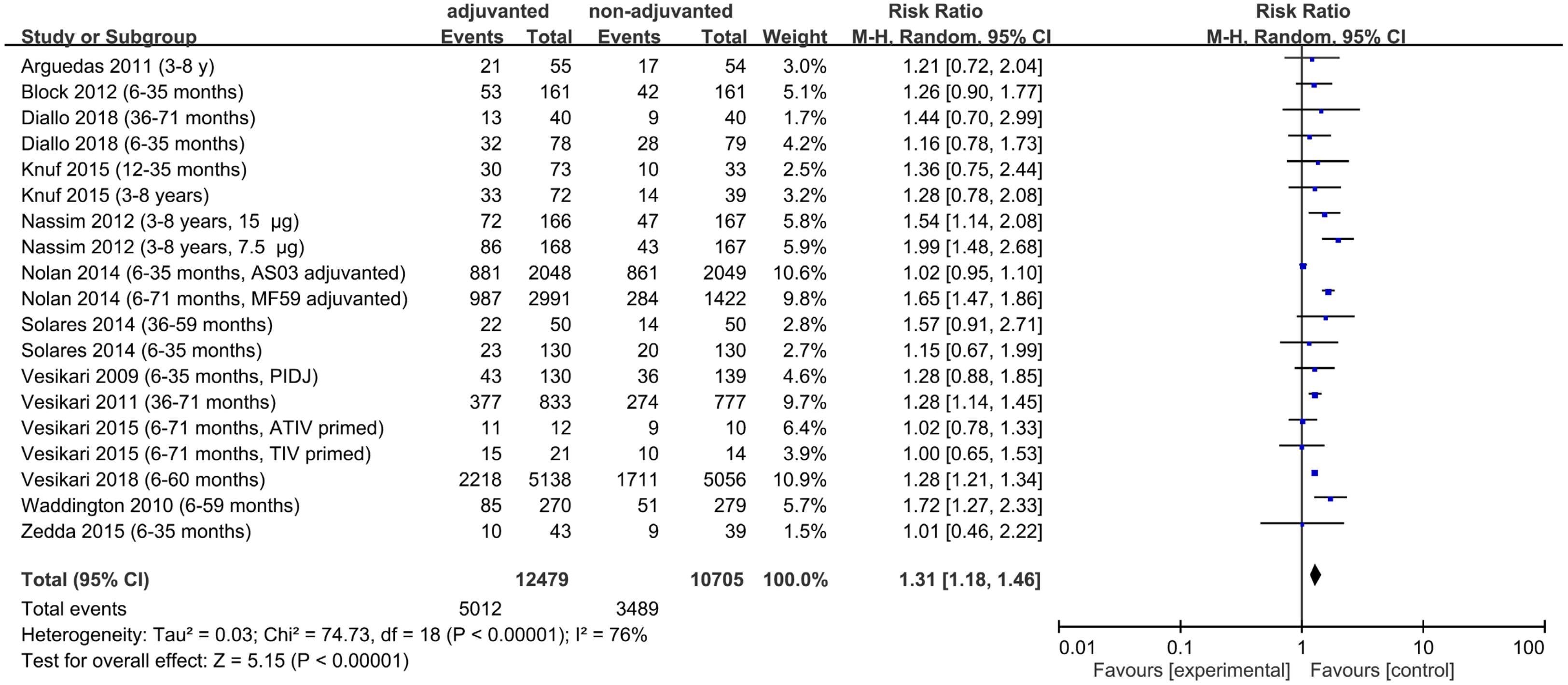

Supplementary Figure 7. Forest plot showing the risk ratios of fever during the 7 days after the first dose among children who received adjuvanted versus non-adjuvanted trivalent vaccines (TIV) or quadrivalent vaccines (QIV).

	adjuvanted		non-adjuvanted			Risk Ratio
Study or Subgroup	Events	Total	Events	Total	Weight	M-H, Random, 95% CI
Block 2012 (6-35 months)	5	161	3	161	1.0%	1.67 [0.41, 6.86]
Diallo 2018 (36-71 months)	13	40	6	40	2.4%	2.17 [0.91, 5.13]
Diallo 2018 (6-35 months)	20	78	9	79	3.3%	2.25 [1.09, 4.63]
Knuf 2015 (12-35 months)	8	73	4	33	1.5%	0.90 [0.29, 2.79]
Knuf 2015 (3-8 years)	4	72	0	39	0.2%	4.93 [0.27, 89.29]
Nassim 2012 (3-8 years, 15 μg)	16	166	9	167	2.8%	1.79 [0.81, 3.93]
Nassim 2012 (3-8 years, 7.5 μg)	14	168	10	167	2.9%	1.39 [0.64, 3.04]
Nolan 2014 (6-35 months, AS03 adjuvanted)	205	2048	143	2049	13.7%	1.43 [1.17, 1.76]
Nolan 2014 (6-71 months, MF59 adjuvanted)	461	3074	130	1443	14.5%	1.66 [1.38, 2.00]
Solares 2014 (36-59 months)	3	50	2	50	0.7%	1.50 [0.26, 8.60]
Solares 2014 (6-35 months)	23	130	16	130	4.5%	1.44 [0.80, 2.59]
Vesikari 2009 (16-47 months, Vaccine)	1	43	0	46	0.2%	3.20 [0.13, 76.60]
Vesikari 2009 (6-35 months, PIDJ)	9	130	6	139	1.9%	1.60 [0.59, 4.38]
Vesikari 2011 (36-71 months)	146	832	52	777	10.4%	2.62 [1.94, 3.54]
Vesikari 2011 (6-35 months)	168	1100	132	992	13.5%	1.15 [0.93, 1.42]
Vesikari 2015 (6-71 months, ATIV primed)	1	12	0	10	0.2%	2.54 [0.11, 56.25]
Vesikari 2015 (6-71 months, TIV primed)	3	21	1	14	0.4%	2.00 [0.23, 17.34]
Vesikari 2018 (6-60 months)	983	5138	529	5056	17.5%	1.83 [1.66, 2.02]
Waddington 2010 (6-59 months)	24	270	26	279	5.3%	0.95 [0.56, 1.62]
Zedda 2015 (6-35 months)	11	43	9	39	3.0%	1.11 [0.51, 2.39]
**Total (95% CI)**		**13649**		**11710**	**100.0%**	**1.59 [1.37, 1.83]**
Total events	2118		1087			

Heterogeneity: Tau² = 0.03; Chi² = 35.01, df = 19 (P = 0.01); I² = 46%

Test for overall effect: Z = 6.29 (P < 0.00001)

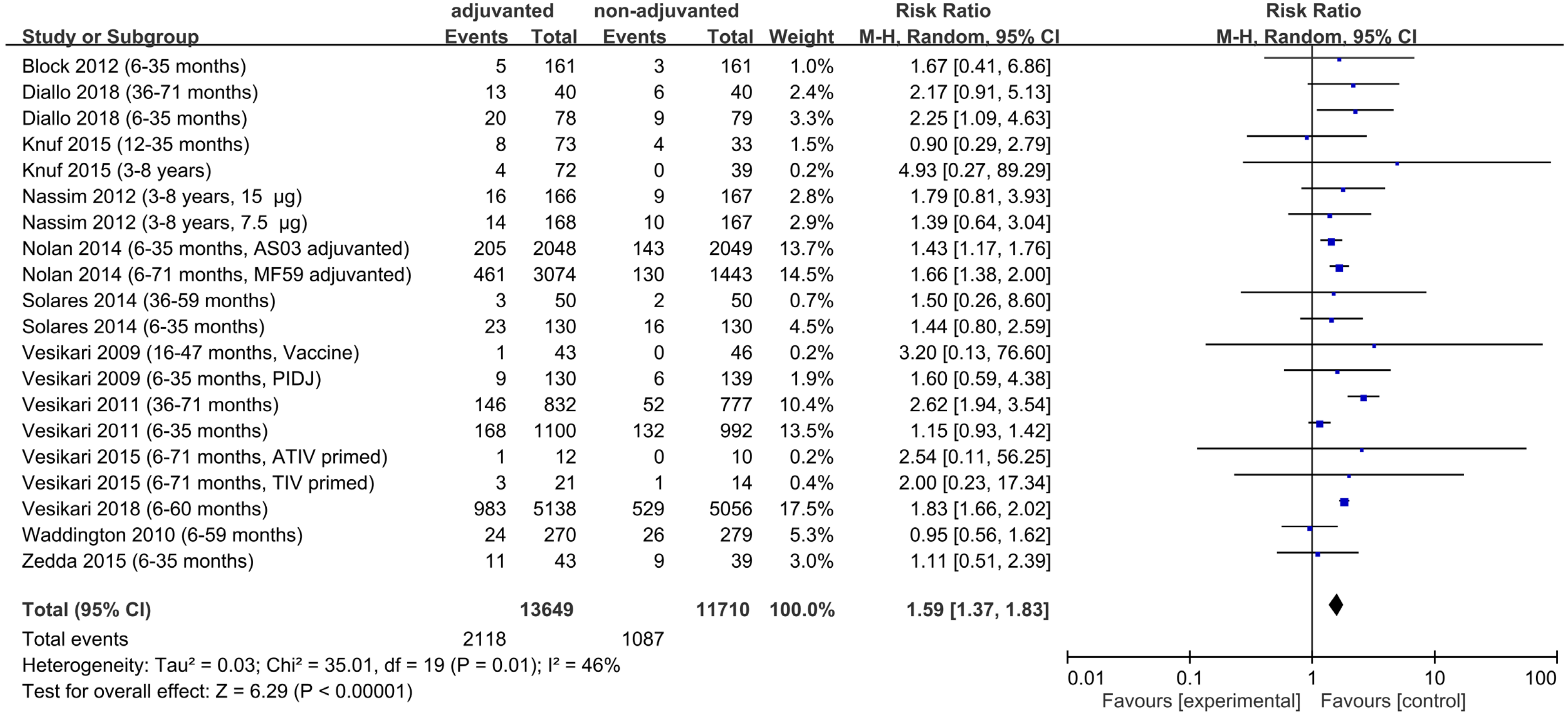

Supplementary Figure 8. Forest plot showing the risk ratios of pain/tenderness at the injection site during the 7 days after the second dose among children who received adjuvanted versus non-adjuvanted trivalent vaccines (TIV) or quadrivalent vaccines (QIV).

	adjuvanted		non-adjuvanted			Risk Ratio
Study or Subgroup	Events	Total	Events	Total	Weight	M-H, Random, 95% CI
Arguedas 2011 (3-8 y)	18	55	19	54	5.7%	0.93 [0.55, 1.57]
Block 2012 (6-35 months)	38	157	27	148	6.7%	1.33 [0.86, 2.06]
Diallo 2018 (36-71 months)	3	39	2	37	1.0%	1.42 [0.25, 8.04]
Diallo 2018 (6-35 months)	12	73	13	68	4.1%	0.86 [0.42, 1.75]
Knuf 2015 (12-35 months)	21	71	9	29	4.6%	0.95 [0.50, 1.83]
Knuf 2015 (3-8 years)	30	70	10	36	5.1%	1.54 [0.85, 2.79]
Nassim 2012 (3-8 years, 15 μg)	48	162	42	162	7.7%	1.14 [0.80, 1.63]
Nassim 2012 (3-8 years, 7.5 μg)	59	163	35	163	7.7%	1.69 [1.18, 2.41]
Nolan 2014 (6-35 months, AS03 adjuvanted)	881	2048	369	2049	10.4%	2.39 [2.15, 2.65]
Nolan 2014 (6-71 months, MF59 adjuvanted)	815	3018	225	1408	10.2%	1.69 [1.48, 1.93]
Solares 2014 (36-59 months)	22	48	14	47	5.6%	1.54 [0.90, 2.63]
Solares 2014 (6-35 months)	14	124	11	121	3.9%	1.24 [0.59, 2.63]
Vesikari 2009 (6-35 months, PIDJ)	34	130	28	139	6.7%	1.30 [0.84, 2.01]
Vesikari 2011 (36-71 months)	352	813	246	757	10.2%	1.33 [1.17, 1.52]
Waddington 2010 (6-59 months)	100	254	47	271	8.4%	2.27 [1.68, 3.07]
Zedda 2015 (6-35 months)	5	39	5	38	2.0%	0.97 [0.31, 3.10]
**Total (95% CI)**		**7264**		**5527**	**100.0%**	**1.45 [1.21, 1.75]**
Total events	2452		1102			

Heterogeneity: Tau² = 0.08; Chi² = 78.20, df = 15 (P < 0.00001); I² = 81%

Test for overall effect: Z = 3.99 (P < 0.0001)

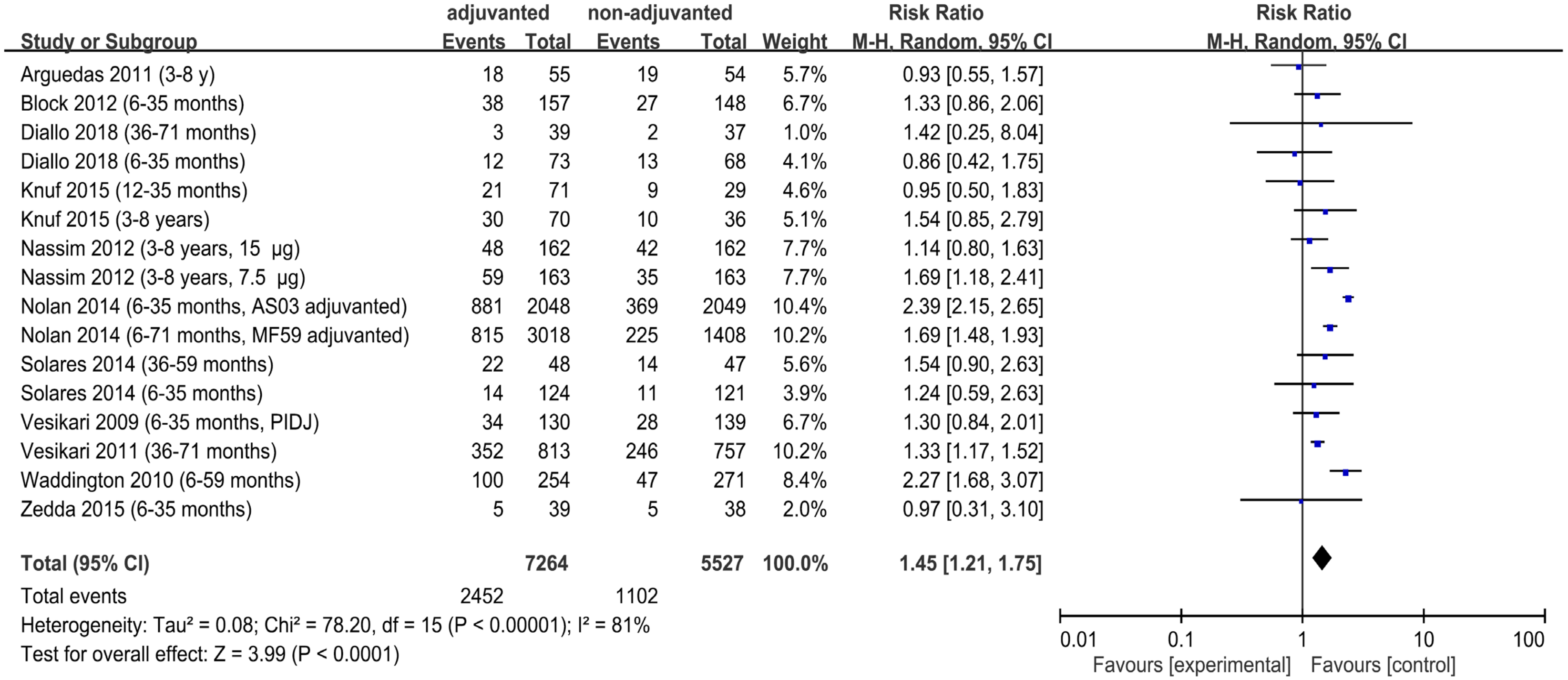

Supplementary Figure 9. Forest plot showing the risk ratios of fever during the 7 days after the second dose among children who received adjuvanted versus non-adjuvanted trivalent vaccines (TIV) or quadrivalent vaccines (QIV).

Study or Subgroup	adjuvanted Events	adjuvanted Total	non-adjuvanted Events	non-adjuvanted Total	Weight	Risk Ratio M-H, Random, 95% CI
Block 2012 (6-35 months)	3	157	2	148	1.3%	1.41 [0.24, 8.34]
Diallo 2018 (36-71 months)	9	39	6	37	3.9%	1.42 [0.56, 3.61]
Diallo 2018 (6-35 months)	22	73	9	68	5.7%	2.28 [1.13, 4.59]
Knuf 2015 (12-35 months)	7	71	1	29	1.0%	2.86 [0.37, 22.22]
Knuf 2015 (3-8 years)	3	70	3	36	1.7%	0.51 [0.11, 2.42]
Nassim 2012 (3-8 years, 15 µg)	7	162	10	162	3.8%	0.70 [0.27, 1.79]
Nassim 2012 (3-8 years, 7.5 µg)	5	163	6	163	2.7%	0.83 [0.26, 2.68]
Nolan 2014 (6-35 months, AS03 adjuvanted)	369	2048	164	2049	13.4%	2.25 [1.89, 2.68]
Nolan 2014 (6-71 months, MF59 adjuvanted)	422	3016	113	1407	13.0%	1.74 [1.43, 2.12]
Solares 2014 (36-59 months)	7	48	10	47	4.2%	0.69 [0.28, 1.65]
Solares 2014 (6-35 months)	24	124	21	121	7.7%	1.12 [0.66, 1.89]
Vesikari 2009 (6-35 months, PIDJ)	7	130	8	139	3.6%	0.94 [0.35, 2.51]
Vesikari 2011 (36-71 months)	137	813	52	757	11.3%	2.45 [1.81, 3.32]
Vesikari 2011 (6-35 months)	171	1075	129	965	12.8%	1.19 [0.96, 1.47]
Waddington 2010 (6-59 months)	57	254	34	271	9.9%	1.79 [1.21, 2.64]
Zedda 2015 (6-35 months)	11	39	5	38	3.7%	2.14 [0.82, 5.59]
**Total (95% CI)**		**8282**		**6437**	**100.0%**	**1.53 [1.24, 1.90]**
Total events	1261		573			

Heterogeneity: Tau² = 0.08; Chi² = 43.12, df = 15 (P = 0.0002); I² = 65%

Test for overall effect: Z = 3.91 (P < 0.0001)

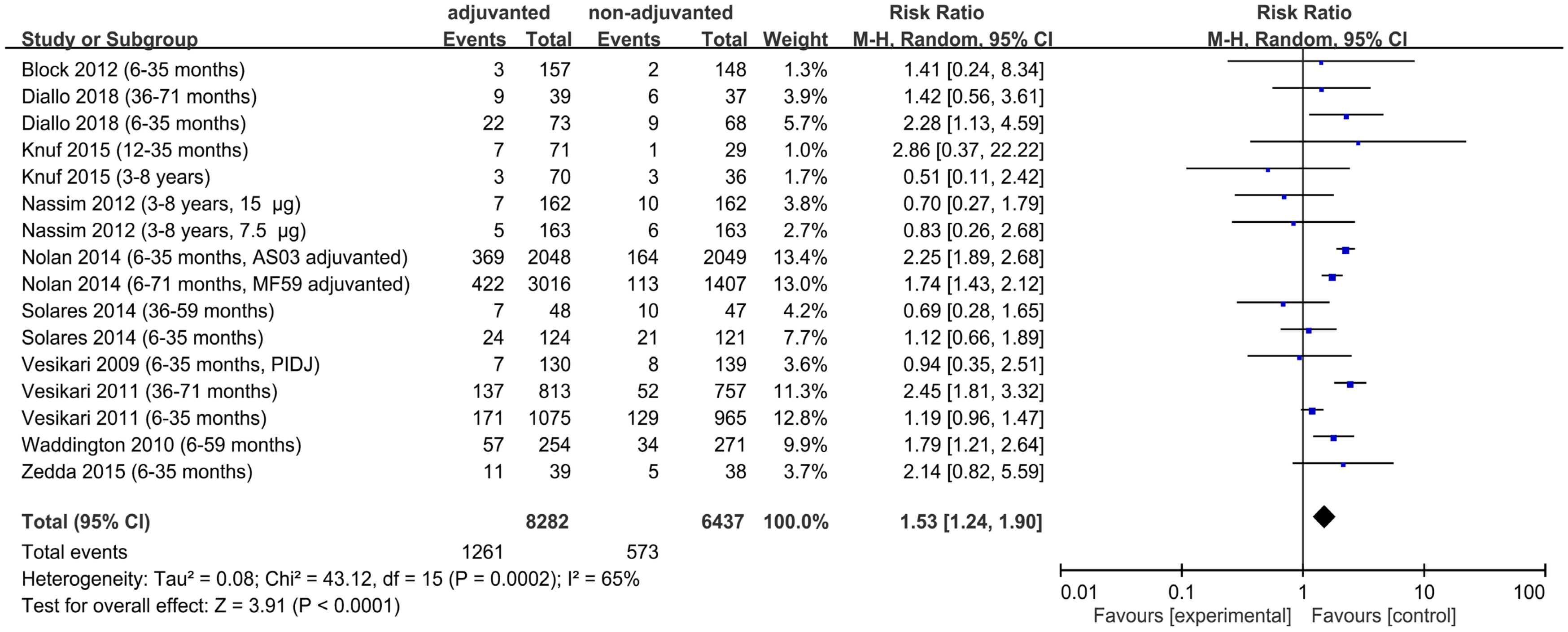

Supplementary Figure 10. Funnel plot with pseudo 95% confidence limits.

(A) Risk ratios of pain/tenderness at the injection site, during the 7 days after the first dose of influenza vaccines.

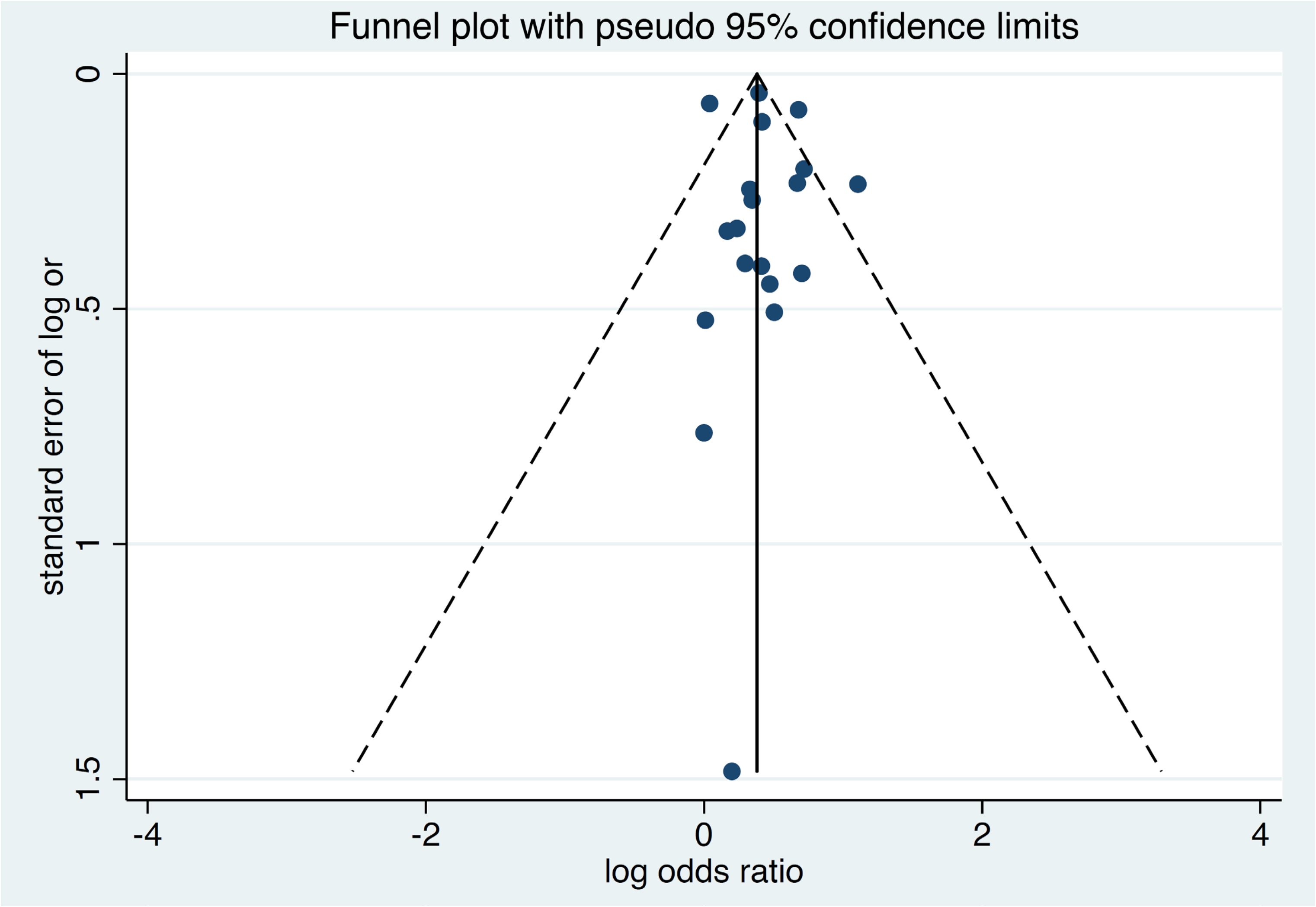

(B) Risk ratios of fever, during the 7 days after the first dose of influenza vaccines.

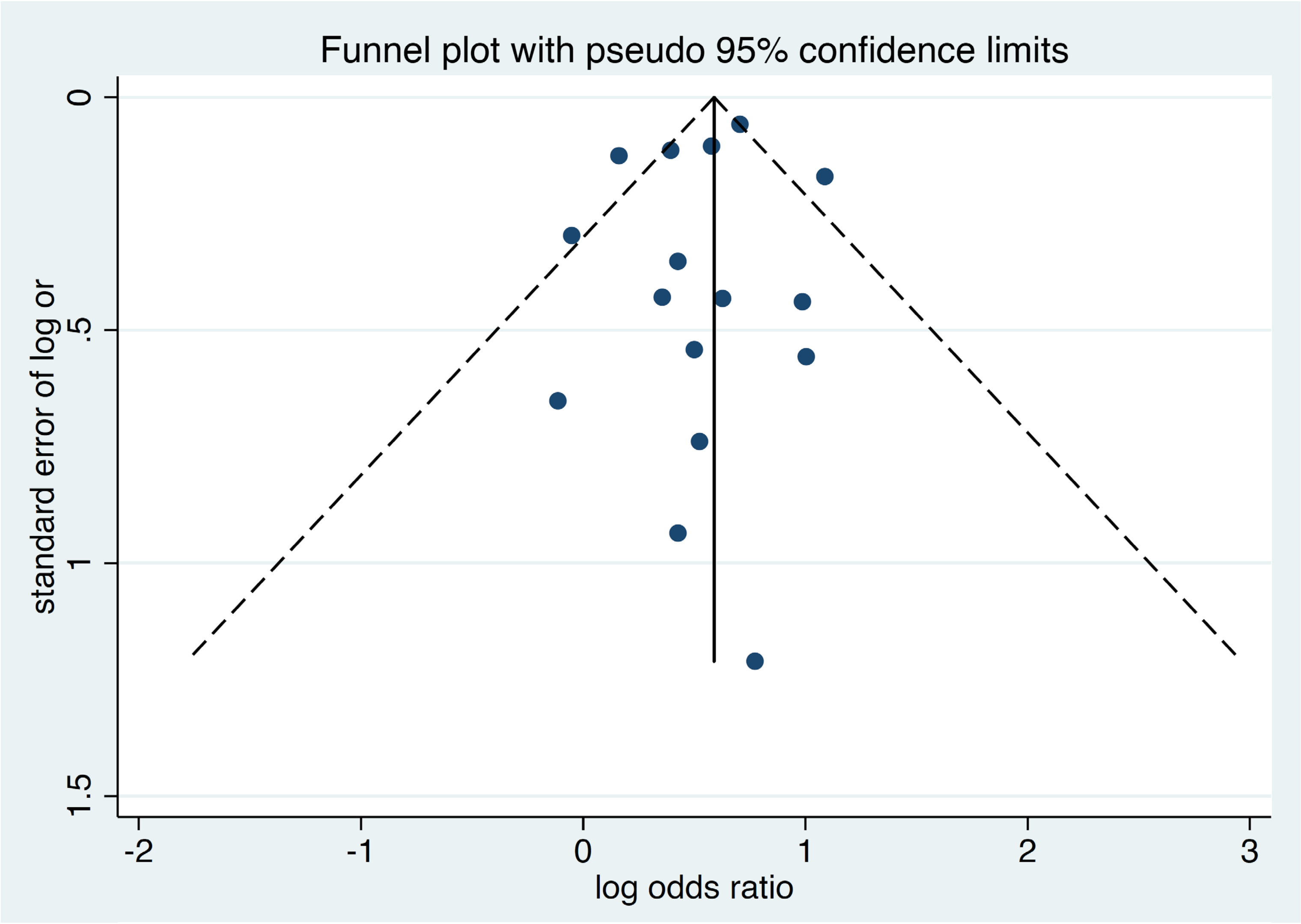

Supplementary Figure 11. Risk of bias summary.

	Random sequence generation (selection bias)	Allocation concealment (selection bias)	Blinding of participants and personnel (performance bias)	Blinding of outcome assessment (detection bias)	Incomplete outcome data (attrition bias)	Selective reporting (reporting bias)	Other bias
Arguedas 2011 (3-8 y)	+	+	?	?	+	+	+
Block 2012 (6-35 months)	+	+	?	?	+	+	+
Cruz-Valdez 2018 (6-71 months)	+	+	+	+	?	?	+
Della Cioppa 2011 (6-11 months)	+	+	?	?	+	+	+
Diallo 2018 (12-35 months)	+	+	+	+	+	+	+
Diallo 2018 (36-71 months)	+	+	+	+	+	+	+
Diallo 2018 (6-11 months)	+	+	+	+	+	+	+
Diallo 2018 (6-35 months)	+	+	+	+	+	+	+
Knuf 2015 (12-35 months)	+	+	?	?	+	+	+
Knuf 2015 (3-8 years)	+	+	?	?	+	+	+
Langley 2012 (12-35 mo)	+	+	?	?	+	+	?
Langley 2012 (3-8 years)	+	+	?	?	+	+	+
Langley 2012 (6-11 months)	+	+	?	?	−	+	+
Nassim 2012 (3-8 years, 15 µg)	+	+	?	?	+	+	+
Nassim 2012 (3-8 years, 7.5 µg)	+	+	?	?	+	+	+
NCT00972816, 2010 (3-8 years,15 µg)	+	+	+	+	+	+	+
NCT00972816, 2010 (3-8 years, 7.5 µg)	+	+	+	?	+	+	+
NCT00996307 2010 (6-35 months)	+	+	+	+	+	+	+
Nolan 2014 (6-35 months, AS03 adjuvanted)	+	+	?	?	?	?	+
Nolan 2014 (6-71 months, MF59 adjuvanted)	+	+	?	?	?	?	+
Solares 2014 (36-59 months)	+	+	?	?	+	+	+
Solares 2014 (6-35 months)	+	+	?	?	+	+	+
Vesikari 2009 (16-47 months, Vaccine)	+	+	?	?	?	?	+
Vesikari 2009 (6-35 months, PIDJ)	+	+	?	?	?	?	+
Vesikari 2011 (36-71 months)	+	+	?	?	+	+	+
Vesikari 2011 (6-35 months)	+	+	?	?	+	+	+
Vesikari 2015 (6-71 months, ATIV primed)	+	+	?	?	?	?	+
Vesikari 2015 (6-71 months, TIV primed)	+	+	?	?	?	?	+
Vesikari 2018 (6-60 months)	+	+	?	?	+	+	+
Waddington 2010 (6-59 months)	+	+	?	?	+	+	+
Zedda 2015 (6-35 months)	+	+	−	−	?	?	?

Supplementary Figure 12. Risk of bias graph.

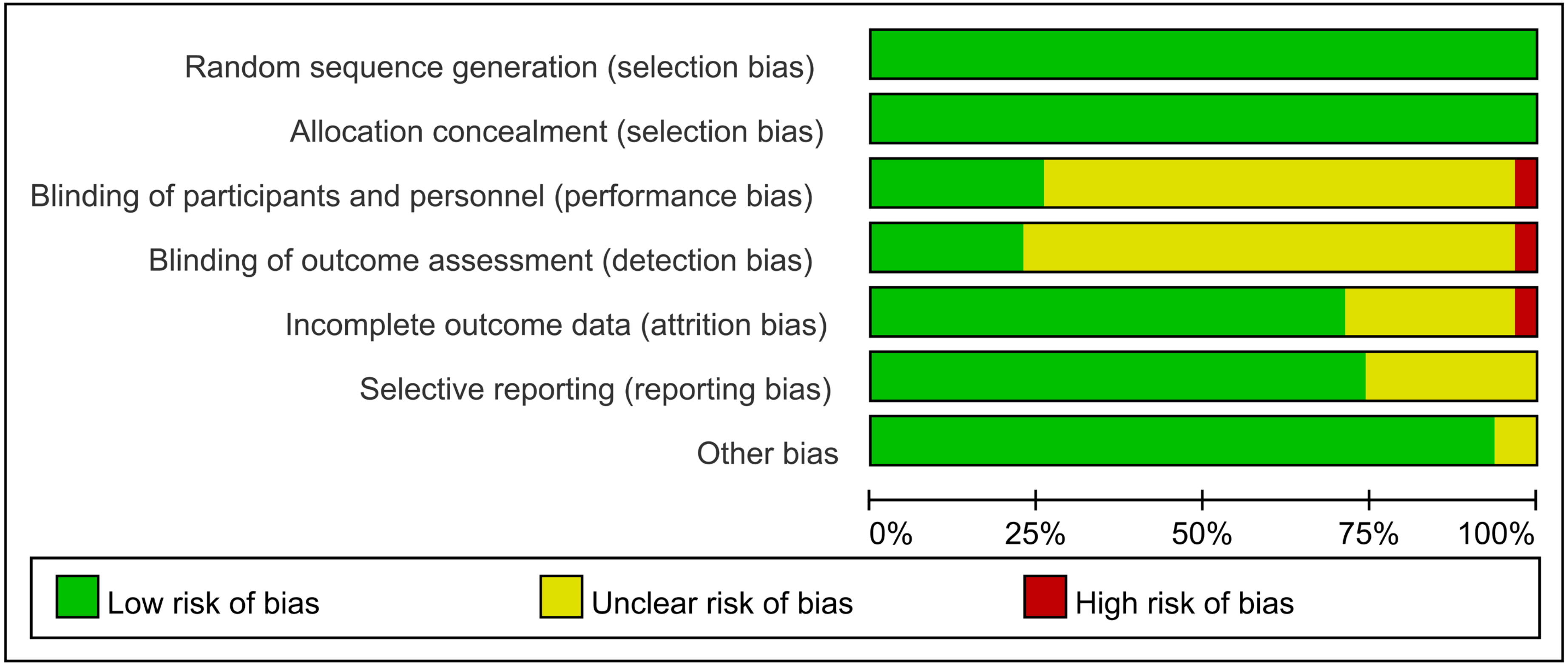

Supplementary Figure 13. Sensitivity analysis by excluding Langley (2012): forest plot showing the ratios of the seroprotection rate (SPR) against H1N1, defined as a ≥1:40 haemagglutinin-inhibition (HI) titer, 21–28 days after the first dose among children who received adjuvanted versus non-adjuvanted trivalent vaccines (TIV) or quadrivalent vaccines (QIV).

Study or Subgroup	adjuvanted Events	adjuvanted Total	Nonadjuvanted Events	Nonadjuvanted Total	Weight	Seroprotection Rate Ratio M-H, Random, 95% CI	Year
**4.1.1 Poor response to non-adjuvanted TIV/QIV (SPR<25%)**							
Vesikari 2011 (6-35 months)	1015	1103	199	995	93.2%	4.60 [4.06, 5.22]	2011
Della Cioppa 2011 (6-11 months)	35	37	9	43	4.3%	4.52 [2.52, 8.12]	2011
Diallo 2018 (6-11 months)	21	30	1	26	0.4%	18.20 [2.63, 126.16]	2018
Diallo 2018 (12-35 months)	27	31	5	34	2.2%	5.92 [2.61, 13.46]	2018
**Subtotal (95% CI)**		**1201**		**1098**	**100.0%**	**4.65 [4.12, 5.25]**	
Total events	1098		214				
Heterogeneity: Tau² = 0.00; Chi² = 2.30, df = 3 (P = 0.51); I² = 0%							
Test for overall effect: Z = 24.86 (P < 0.00001)							
**4.1.2 Moderate response to non-adjuvanted TIV/QIV (SPR 25% to 75%)**							
Arguedas 2011 (3-8 y)	43	46	53	75	12.5%	1.32 [1.12, 1.56]	2011
Vesikari 2011 (36-71 months)	834	834	490	777	17.5%	1.59 [1.50, 1.67]	2011
Block 2012 (6-35 months)	109	127	46	124	9.3%	2.31 [1.82, 2.94]	2012
Nassim 2012 (3-8 years, 7.5 μg)	137	156	72	156	11.8%	1.90 [1.59, 2.28]	2012
Nassim 2012 (3-8 years, 15 μg)	144	156	90	155	13.7%	1.59 [1.38, 1.83]	2012
Knuf 2015 (3-8 years)	50	60	20	31	7.7%	1.29 [0.97, 1.72]	2015
Knuf 2015 (12-35 months)	41	53	8	25	2.7%	2.42 [1.34, 4.36]	2015
Vesikari 2018 (6-60 months)	678	922	467	866	16.8%	1.36 [1.27, 1.47]	2018
Diallo 2018 (36-71 months)	35	38	21	33	8.0%	1.45 [1.10, 1.90]	2018
**Subtotal (95% CI)**		**2392**		**2242**	**100.0%**	**1.58 [1.42, 1.75]**	
Total events	2071		1267				
Heterogeneity: Tau² = 0.02; Chi² = 35.59, df = 8 (P < 0.0001); I² = 78%							
Test for overall effect: Z = 8.64 (P < 0.00001)							
**4.1.3 Good response to non-adjuvanted TIV/QIV (SPR>75%)**							
Vesikari 2009 (16-47 months, Vaccine)	41	41	40	40	57.3%	1.00 [0.95, 1.05]	2009
Solares 2014 (36-59 months)	22	23	20	20	8.6%	0.96 [0.85, 1.09]	2014
Solares 2014 (6-35 months)	90	97	94	102	20.6%	1.01 [0.93, 1.09]	2014
Vesikari 2015 (6-71 months, ATIV primed)	16	17	11	12	3.0%	1.03 [0.83, 1.26]	2015
Vesikari 2015 (6-71 months, TIV primed)	23	23	14	14	10.6%	1.00 [0.90, 1.12]	2015
**Subtotal (95% CI)**		**201**		**188**	**100.0%**	**1.00 [0.96, 1.04]**	
Total events	192		179				
Heterogeneity: Tau² = 0.00; Chi² = 0.51, df = 4 (P = 0.97); I² = 0%							
Test for overall effect: Z = 0.07 (P = 0.94)							

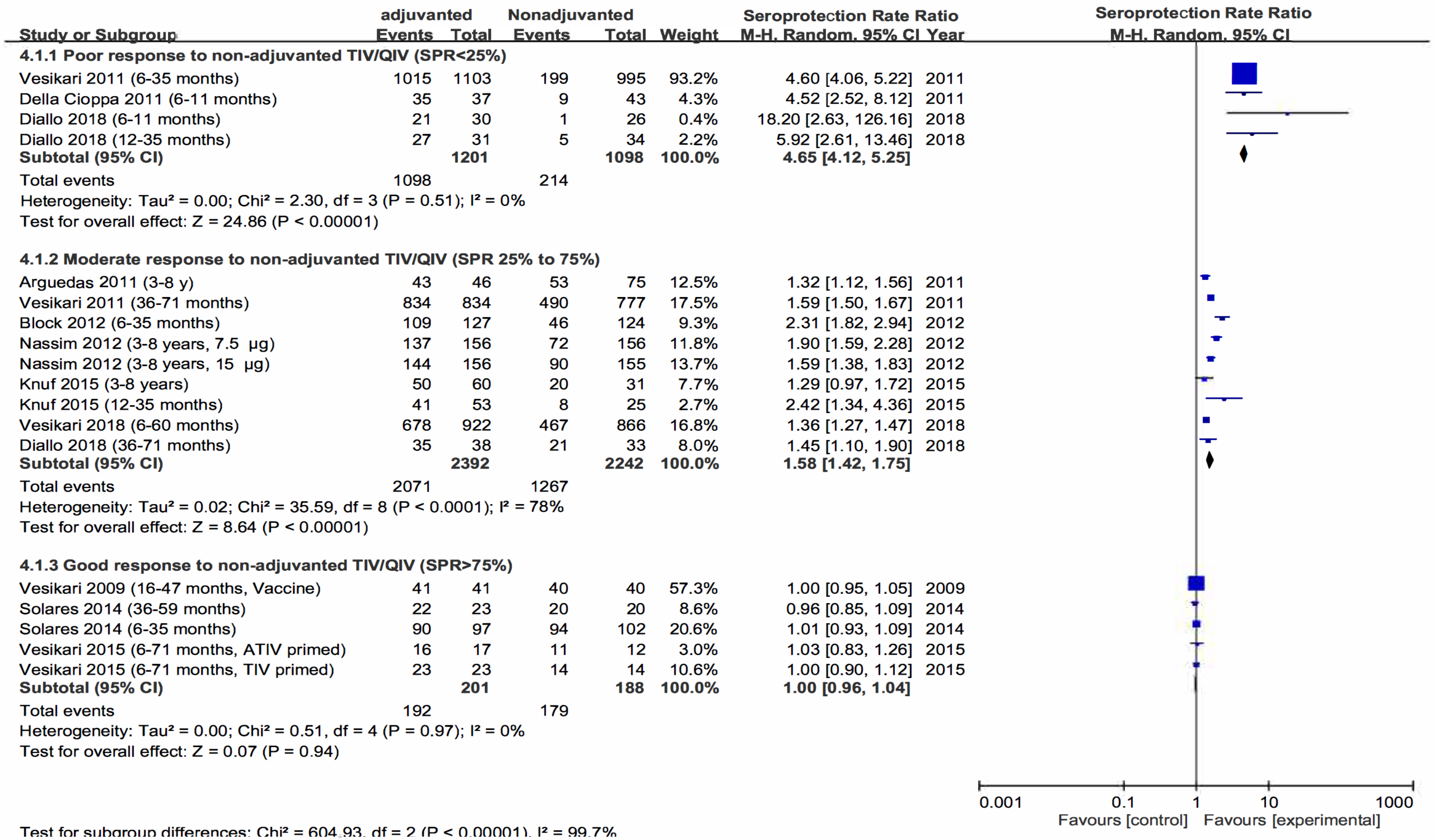

Test for subgroup differences: Chi² = 604.93, df = 2 (P < 0.00001), I² = 99.7%

Supplementary Figure 14. Sensitivity analysis by excluding Langley (2012): meta-regression showing a linear relationship (slope = 0.79, P <0.001) between the ratios of seroprotection rates against H1N1 after the first dose and the inverse of seroprotection rate in non-adjuvanted arm, with an R^2 of 99.81%.

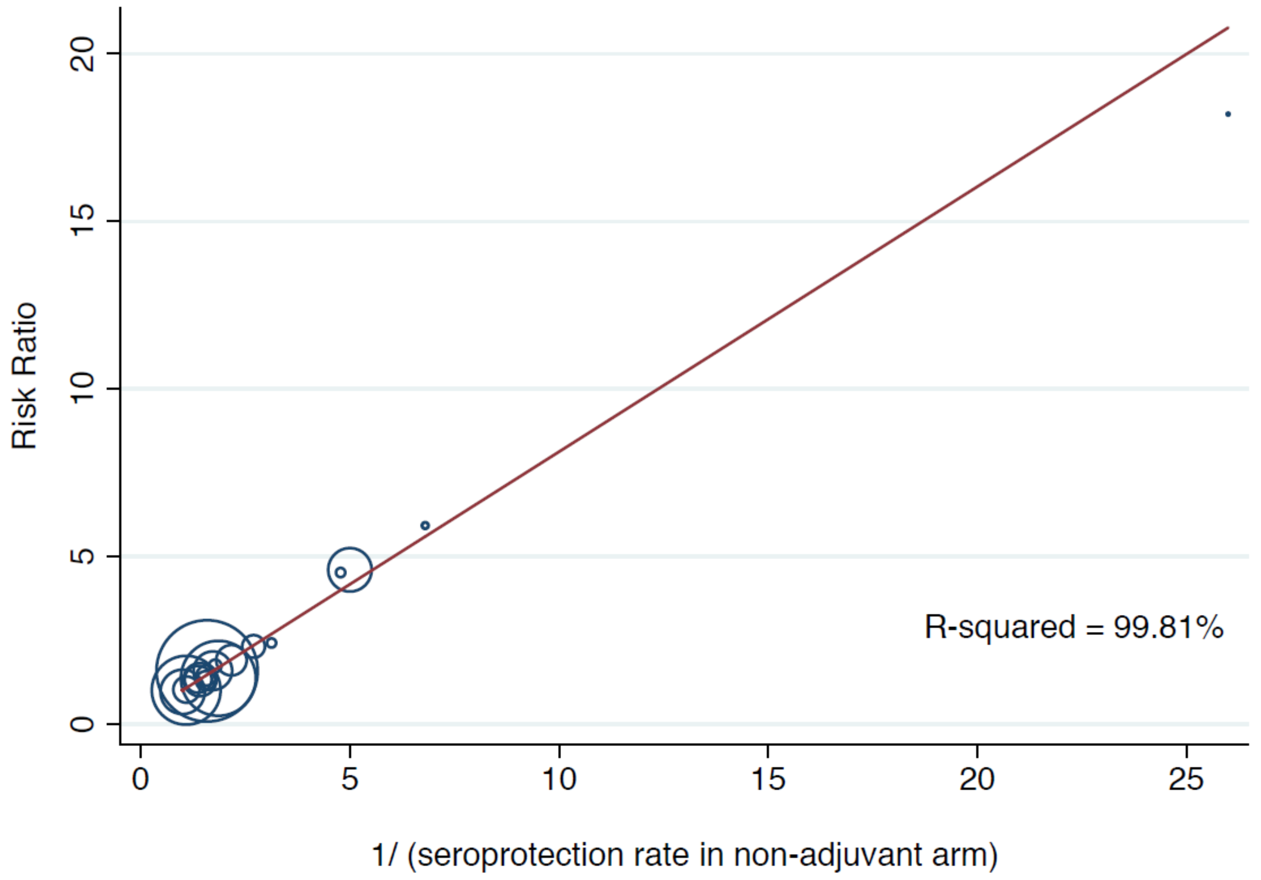

Supplementary Figure 15. Sensitivity analysis by excluding Zedda (2015): forest plot showing the ratios of the seroprotection rate (SPR) against H3N2, defined as a ≥1:40 haemagglutinin-inhibition (HI) titer, 21–28 days after the second dose among children who received adjuvanted versus non-adjuvanted trivalent vaccines (TIV) or quadrivalent vaccines (QIV).

Study or Subgroup	adjuvanted Events	adjuvanted Total	Nonadjuvanted Events	Nonadjuvanted Total	Weight	Seroprotection Rate Ratio M-H, Random, 95% CI	Year
**4.4.1 Moderate response to non-adjuvanted TIV/QIV (SPR 25% to 75%)**							
Della Cioppa 2011 (6-11 months)	36	36	30	42	32.6%	1.39 [1.14, 1.69]	2011
Vesikari 2011 (6-35 months)	1075	1103	448	995	33.7%	2.16 [2.02, 2.32]	2011
Vesikari 2018 (6-60 months)	669	922	619	866	33.7%	1.02 [0.96, 1.08]	2018
**Subtotal (95% CI)**		**2061**		**1903**	**100.0%**	**1.45 [0.81, 2.59]**	
Total events	1780		1097				
Heterogeneity: Tau² = 0.26; Chi² = 283.26, df = 2 (P < 0.00001); I² = 99%							
Test for overall effect: Z = 1.26 (P = 0.21)							
**4.4.2 Good response non-adjuvanted TIV/QIV (SPR >75%)**							
Vesikari 2009 (6-35 months, PIDJ)	104	104	117	118	12.5%	1.01 [0.98, 1.03]	2009
Vesikari 2011 (36-71 months)	817	834	680	777	12.4%	1.12 [1.09, 1.15]	2011
Solares 2014 (6-35 months)	97	97	93	102	10.7%	1.10 [1.03, 1.17]	2014
Solares 2014 (36-59 months)	23	23	20	20	9.1%	1.00 [0.92, 1.09]	2014
Nolan 2014 (6-71 months, MF59 adjuvanted)	266	266	385	389	12.8%	1.01 [1.00, 1.02]	2014
Cruz-Valdez 2018 (6-71 months)	114	114	108	112	11.9%	1.04 [1.00, 1.08]	2018
Diallo 2018 (6-11 months)	30	30	25	26	8.4%	1.04 [0.94, 1.15]	2018
Diallo 2018 (36-71 months)	38	38	33	33	11.2%	1.00 [0.95, 1.06]	2018
Diallo 2018 (12-35 months)	31	31	34	34	10.9%	1.00 [0.94, 1.06]	2018
**Subtotal (95% CI)**		**1537**		**1611**	**100.0%**	**1.03 [0.98, 1.09]**	
Total events	1520		1495				
Heterogeneity: Tau² = 0.00; Chi² = 142.12, df = 8 (P < 0.00001); I² = 94%							
Test for overall effect: Z = 1.34 (P = 0.18)							

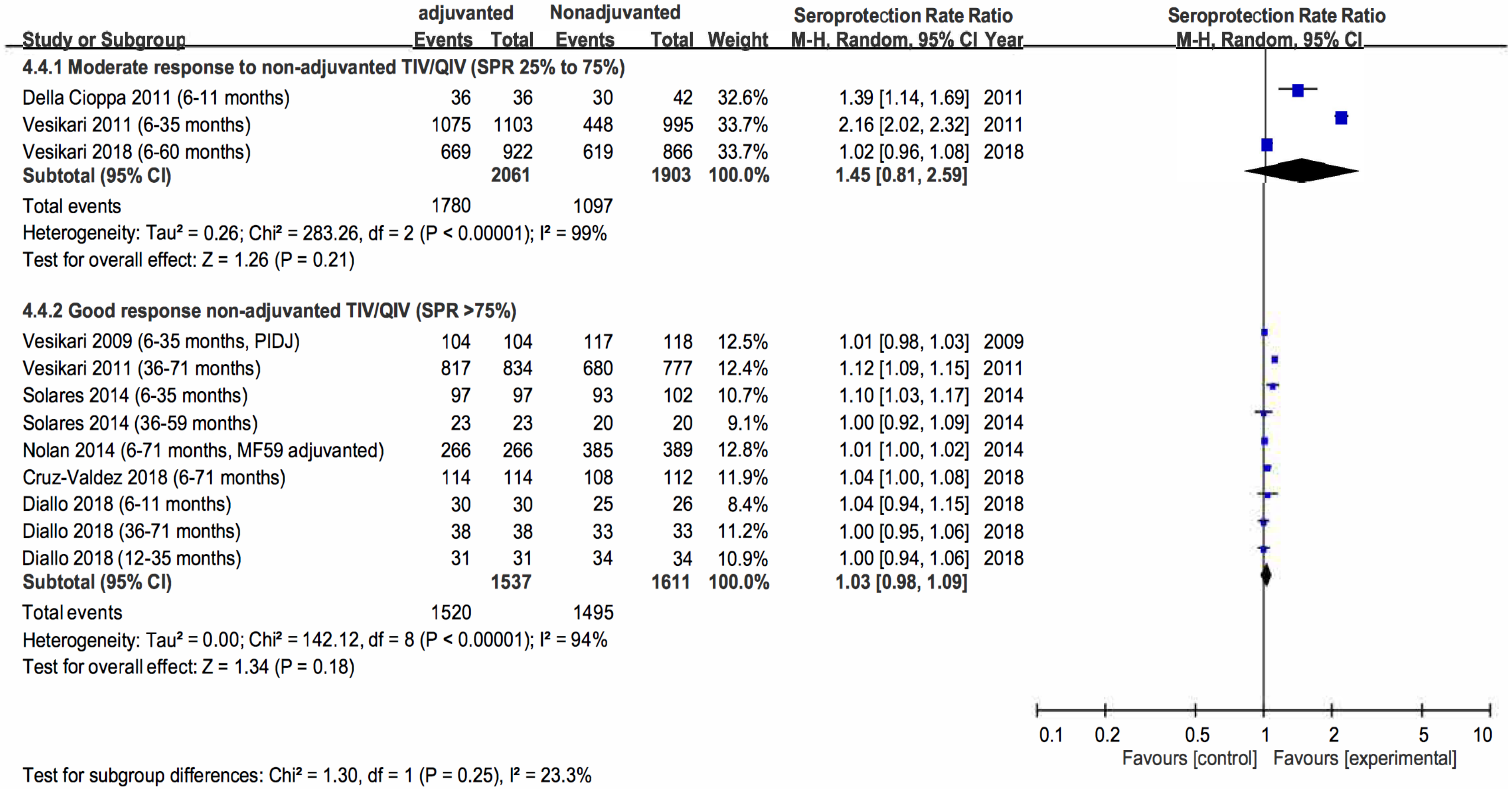

Test for subgroup differences: Chi² = 1.30, df = 1 (P = 0.25), I² = 23.3%

Supplementary Figure 16. Sensitivity analysis by excluding Zedda (2015): meta-regression showing a linear relationship (slope = 0.91, P <0.001) between the ratios of seroprotection rates against H3N2 after the second dose and the inverse of seroprotection rate in non-adjuvanted arm, with an R^2 of 88.54%.

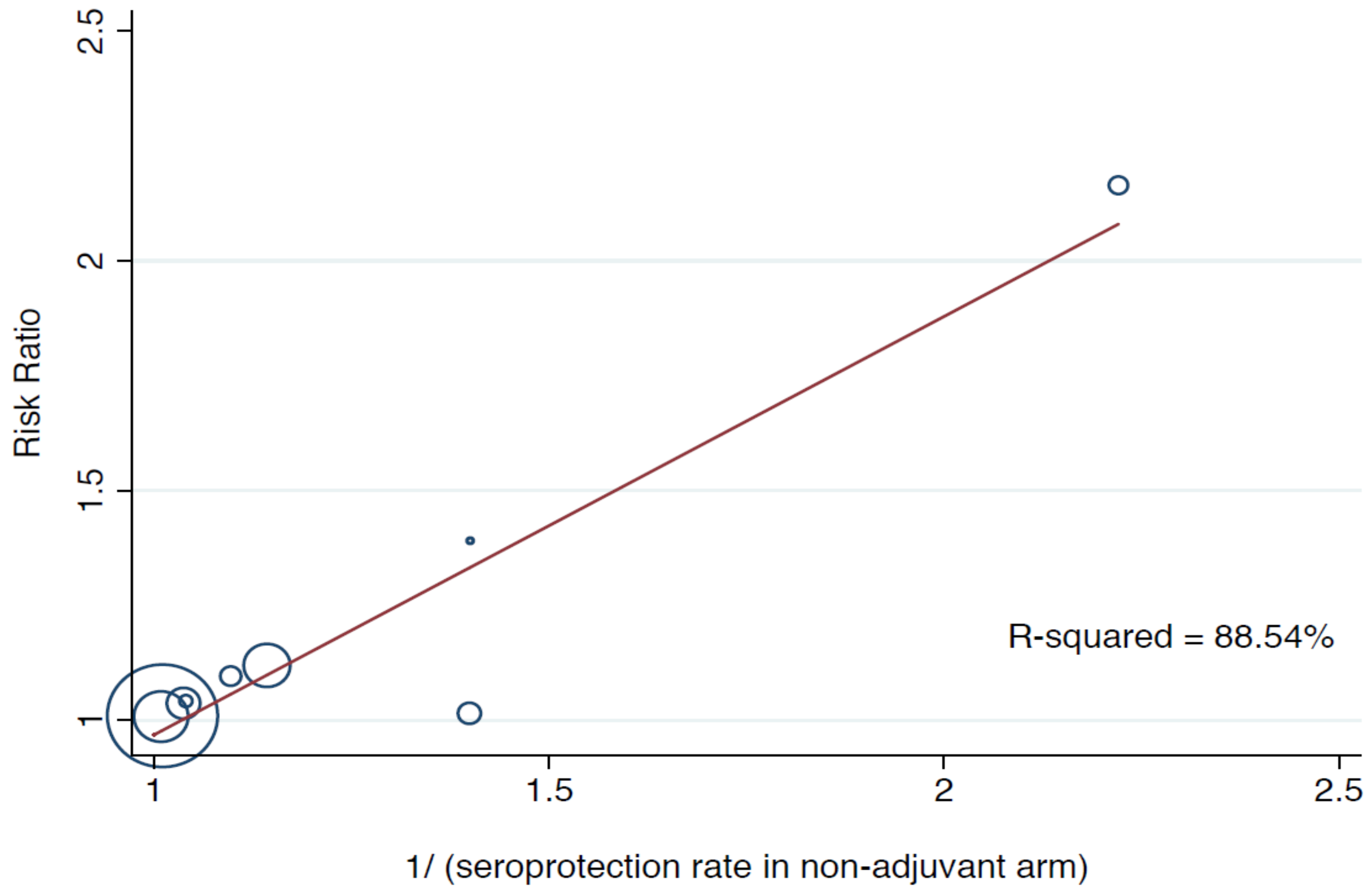

Supplementary Figure 17. Sensitivity analysis by excluding Zedda (2015): forest plot showing the ratios of the seroprotection rate (SPR) against influenza B, defined as a ≥1:40 haemagglutinin-inhibition (HI) titer, 21–28 days after the second dose among children who received adjuvanted versus non-adjuvanted trivalent vaccines (TIV) or quadrivalent vaccines (QIV).

	adjuvanted		Nonadjuvanted			Seroprotection Rate Ratio	
Study or Subgroup	Events	Total	Events	Total	Weight	M-H, Random, 95% CI	Year
**4.6.1 Poor response to non-adjuvanted TIV/QIV (SPR <25%)**							
Vesikari 2011 (6-35 months)	965	1103	174	995	85.8%	5.00 [4.36, 5.74]	2011
Della Cioppa 2011 (6-11 months)	35	36	5	42	14.2%	8.17 [3.58, 18.63]	2011
**Subtotal (95% CI)**		**1139**		**1037**	**100.0%**	**5.36 [3.84, 7.50]**	
Total events	1000		179				
Heterogeneity: Tau² = 0.03; Chi² = 1.32, df = 1 (P = 0.25); I² = 24%							
Test for overall effect: Z = 9.82 (P < 0.00001)							
**4.6.2 Moderate response to non-adjuvanted TIV/QIV (SPR 25% to 75%)**							
Vesikari 2009 (6-35 months, PIDJ)	103	104	39	118	17.1%	3.00 [2.32, 3.88]	2009
Vesikari 2011 (36-71 months)	817	834	466	777	23.5%	1.63 [1.54, 1.73]	2011
Solares 2014 (6-35 months)	87	97	57	102	19.9%	1.60 [1.33, 1.93]	2014
Cruz-Valdez 2018 (6-71 months)	95	114	36	112	16.2%	2.59 [1.96, 3.43]	2018
Vesikari 2018 (6-60 months)	720	922	505	866	23.3%	1.34 [1.25, 1.43]	2018
**Subtotal (95% CI)**		**2071**		**1975**	**100.0%**	**1.86 [1.52, 2.27]**	
Total events	1822		1103				
Heterogeneity: Tau² = 0.04; Chi² = 60.69, df = 4 (P < 0.00001); I² = 93%							
Test for overall effect: Z = 6.08 (P < 0.00001)							
**4.6.3 Good response to non-adjuvanted TIV/QIV (SPR >75%)**							
Solares 2014 (36-59 months)	18	23	16	20	5.2%	0.98 [0.72, 1.33]	2014
Nolan 2014 (6-71 months, MF59 adjuvanted)	261	266	332	389	32.6%	1.15 [1.10, 1.20]	2014
Diallo 2018 (36-71 months)	38	38	32	33	26.0%	1.03 [0.95, 1.12]	2018
Diallo 2018 (6-11 months)	30	30	21	26	10.6%	1.24 [1.02, 1.50]	2018
Diallo 2018 (12-35 months)	31	31	33	34	25.6%	1.03 [0.95, 1.12]	2018
**Subtotal (95% CI)**		**388**		**502**	**100.0%**	**1.09 [1.01, 1.17]**	
Total events	378		434				
Heterogeneity: Tau² = 0.00; Chi² = 11.56, df = 4 (P = 0.02); I² = 65%							
Test for overall effect: Z = 2.13 (P = 0.03)							

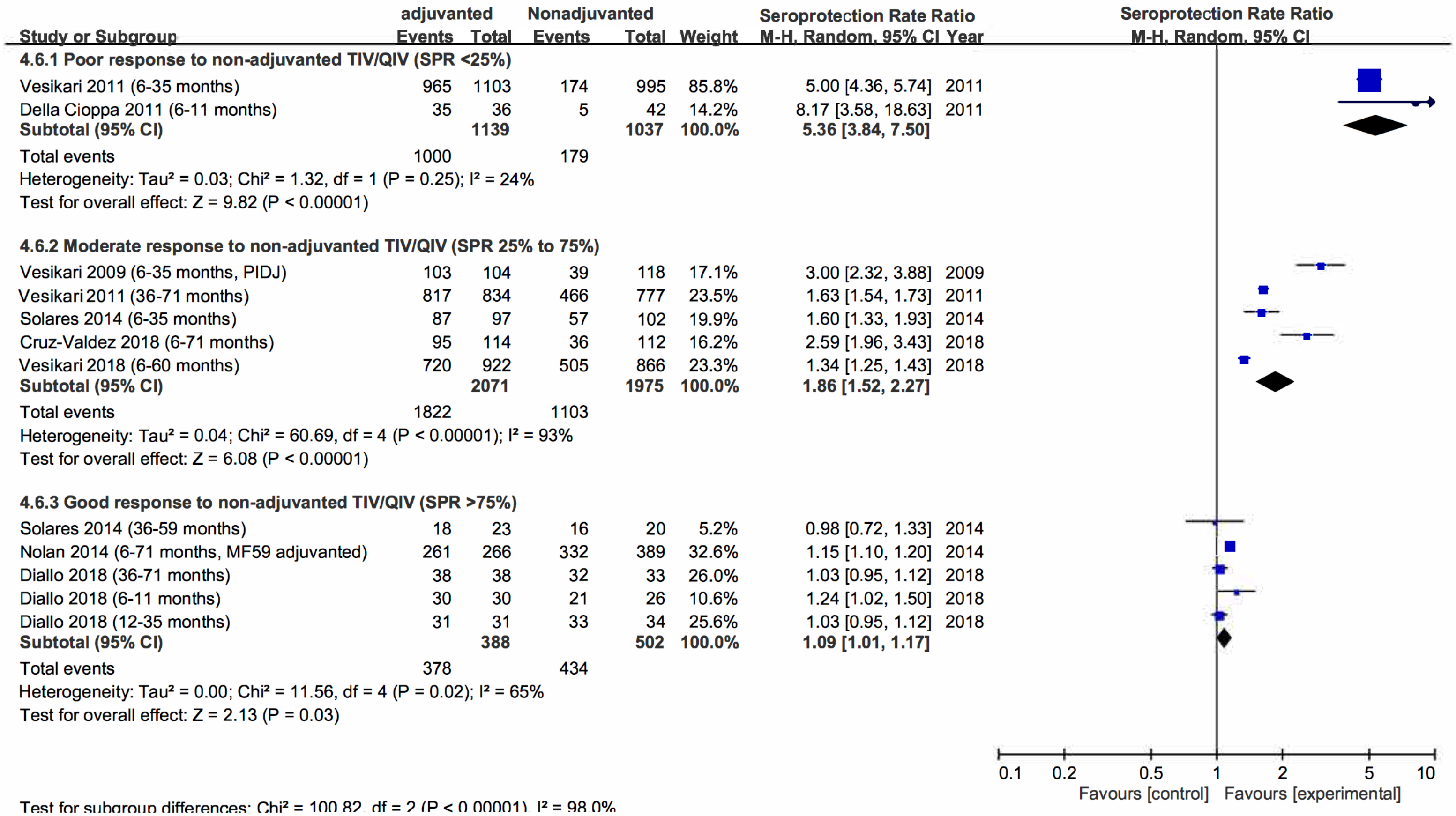

Test for subgroup differences: Chi² = 100.82, df = 2 (P < 0.00001), I² = 98.0%

Supplementary Figure 18. Sensitivity analysis by excluding Zedda (2015): meta-regression showing a linear relationship (slope = 0.88, P <0.001) between the ratios of seroprotection rates against influenza B after the second dose and the inverse of seroprotection rate in non-adjuvanted arm, with an R^2 of 99.63%.

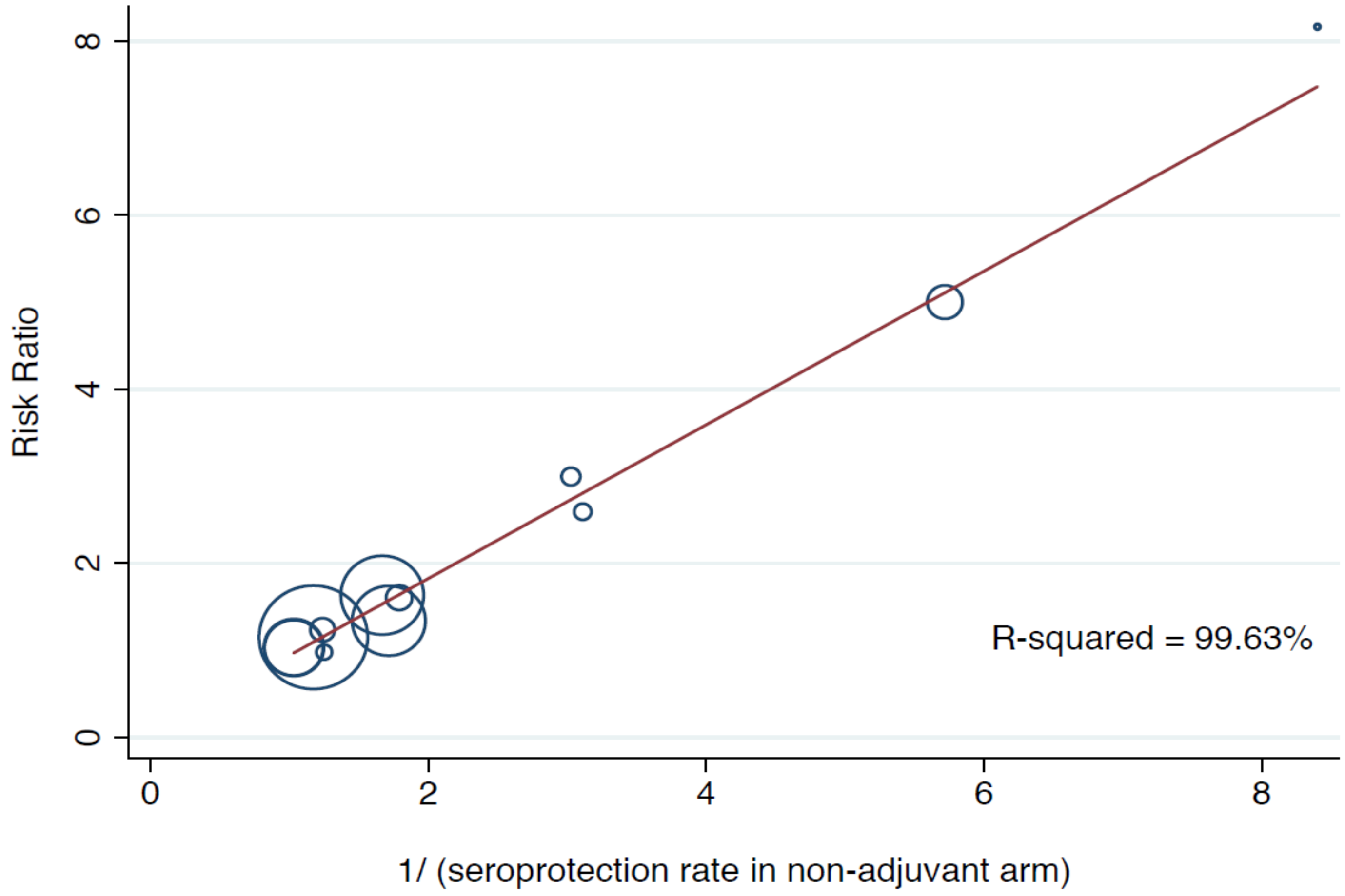

Supplementary Figure 19. Sensitivity analysis by excluding the 12 trials which did not compare the same vaccine with or without adjuvants (using adjuvanted and non-adjuvanted vaccines from different manufacturers, or using antigen-sparing design with reduced antigen dose in adjuvanted vaccines): forest plot showing the risk ratios of RT-PCR-confirmed influenza among children who received adjuvanted versus non-adjuvanted trivalent vaccines (TIV) or quadrivalent vaccines (QIV).

	Experimental		Control			Risk Ratio
Study or Subgroup	Events	Total	Events	Total	Weight	M-H, Random, 95% CI
**1.4.1 All-naive group**						
Vesikari 2011 (36-71 months)	4	834	25	777	38.8%	0.15 [0.05, 0.43]
Vesikari 2011 (6-35 months)	9	1103	25	995	61.2%	0.32 [0.15, 0.69]
**Subtotal (95% CI)**		**1937**		**1772**	**100.0%**	**0.24 [0.11, 0.51]**
Total events	13		50			

Heterogeneity: Tau² = 0.09; Chi² = 1.41, df = 1 (P = 0.23); I² = 29%
Test for overall effect: Z = 3.73 (P = 0.0002)

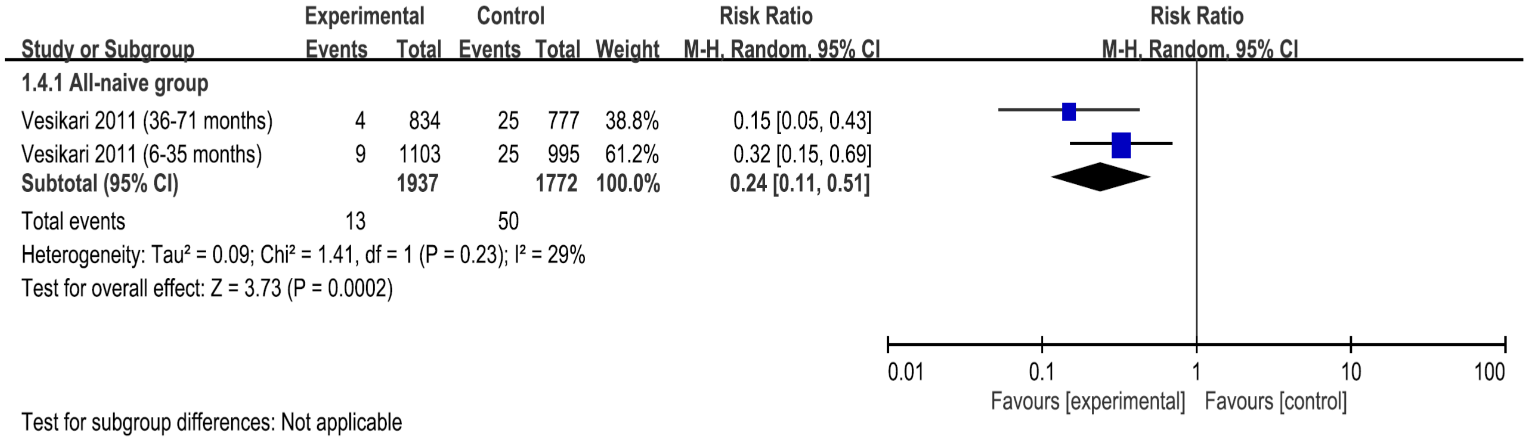

Test for subgroup differences: Not applicable

Supplementary Figure 20. Sensitivity analysis by excluding the 12 trials which did not compare the same vaccine with or without adjuvants (using adjuvanted and non-adjuvanted vaccines from different manufacturers, or using antigen-sparing design with reduced antigen dose in adjuvanted vaccines): forest plot showing the ratios of the seroprotection rate (SPR) against H1N1, defined as a ≥1:40 haemagglutinin-inhibition (HI) titer, 21–28 days after the first dose among children who received adjuvanted versus non-adjuvanted trivalent vaccines (TIV) or quadrivalent vaccines (QIV).

Study or Subgroup	adjuvanted Events	adjuvanted Total	Nonadjuvanted Events	Nonadjuvanted Total	Weight	Seroprotection Rate Ratio M-H, Random, 95% CI
**4.8.1 Poor reponse to non-adjuvanted TIV/QIV (SPR <25%)**						
Vesikari 2011 (6-35 months)	1015	1103	199	995	100.0%	4.60 [4.06, 5.22]
**Subtotal (95% CI)**		**1103**		**995**	**100.0%**	**4.60 [4.06, 5.22]**
Total events	1015		199			
Heterogeneity: Not applicable						
Test for overall effect: Z = 23.84 (P < 0.00001)						
**4.8.2 Moderate response to non-adjuvanted TIV/QIV (SPR 25% to 75%)**						
Block 2012 (6-35 months)	109	127	46	124	17.8%	2.31 [1.82, 2.94]
Nassim 2012 (3-8 years, 15 μg)	144	156	90	155	26.2%	1.59 [1.38, 1.83]
Nassim 2012 (3-8 years, 7.5 μg)	137	156	72	156	22.7%	1.90 [1.59, 2.28]
Vesikari 2011 (36-71 months)	834	834	490	777	33.3%	1.59 [1.50, 1.67]
**Subtotal (95% CI)**		**1273**		**1212**	**100.0%**	**1.77 [1.53, 2.04]**
Total events	1224		698			
Heterogeneity: Tau² = 0.02; Chi² = 12.97, df = 3 (P = 0.005); I² = 77%						
Test for overall effect: Z = 7.74 (P < 0.00001)						
**4.8.3 Good response to non-adjuvanted TIV/QIV (SPR >75%)**						
Vesikari 2015 (6-71 months, ATIV primed)	16	17	11	12	22.1%	1.03 [0.83, 1.26]
Vesikari 2015 (6-71 months, TIV primed)	23	23	14	14	77.9%	1.00 [0.90, 1.12]
**Subtotal (95% CI)**		**40**		**26**	**100.0%**	**1.01 [0.91, 1.11]**
Total events	39		25			
Heterogeneity: Tau² = 0.00; Chi² = 0.06, df = 1 (P = 0.81); I² = 0%						
Test for overall effect: Z = 0.12 (P = 0.91)						

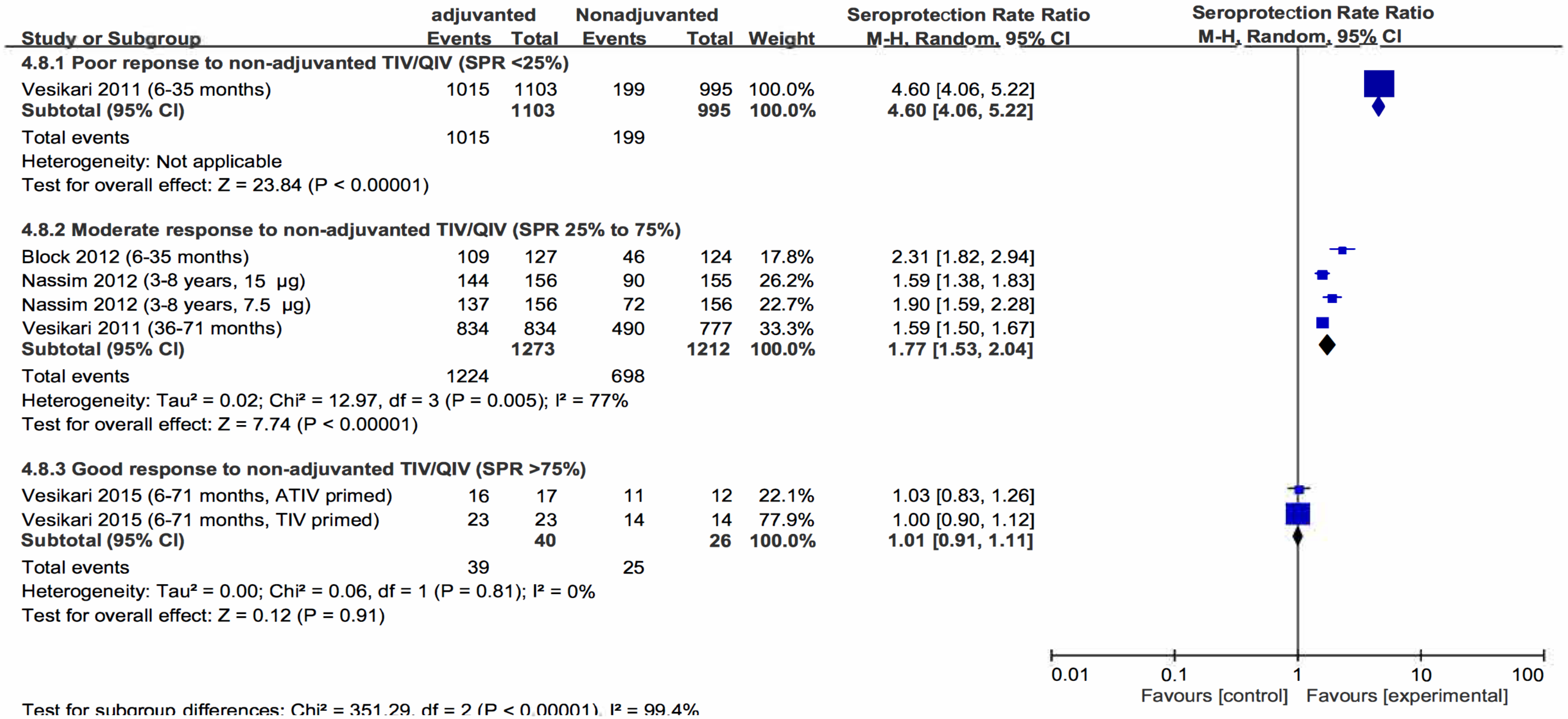

Test for subgroup differences: Chi² = 351.29, df = 2 (P < 0.00001), I² = 99.4%

Supplementary Figure 21. Sensitivity analysis by excluding the 12 trials which did not compare the same vaccine with or without adjuvants (using adjuvanted and non-adjuvanted vaccines from different manufacturers, or using antigen-sparing design with reduced antigen dose in adjuvanted vaccines): meta-regression showing a linear relationship (slope = 0.90, P <0.001) between the ratios of the seroprotection rate against H1N1 after the first dose and the inverse of seroprotection rate in non-adjuvanted arm, with an R^2 of 99.56%.

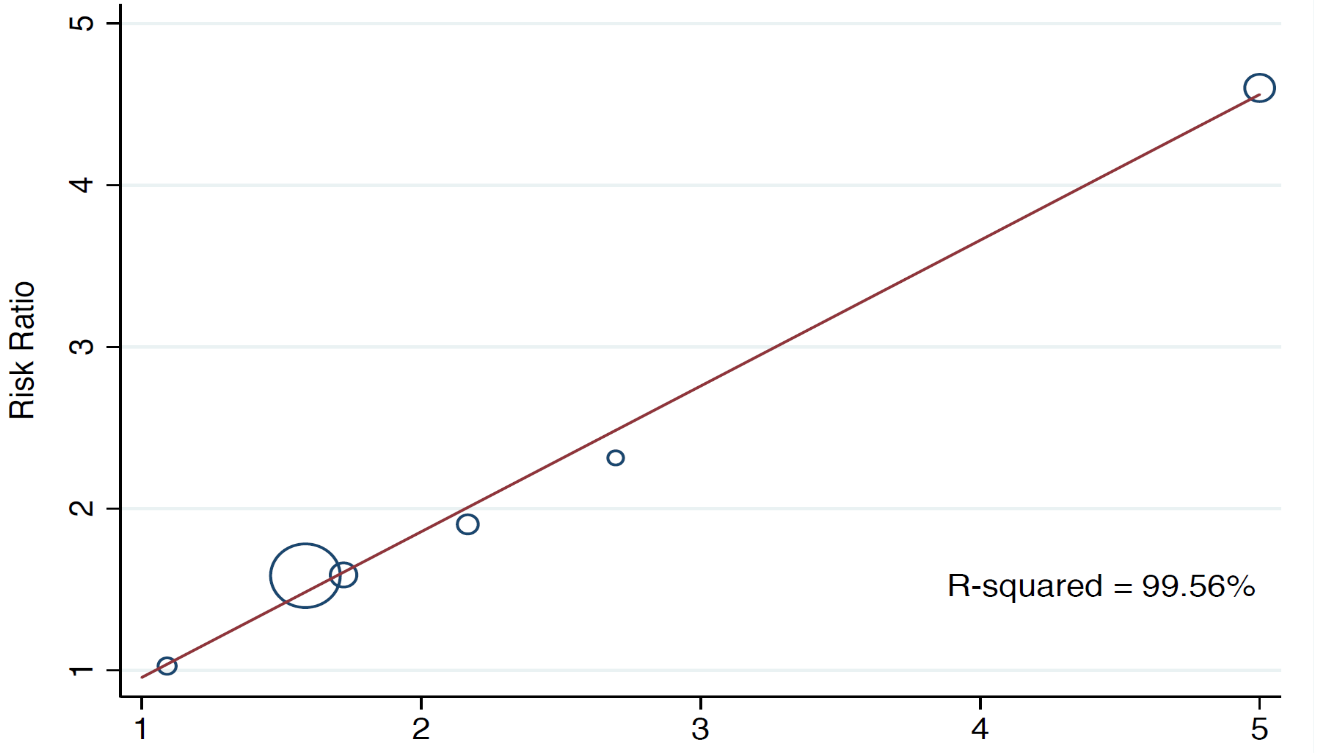

Supplementary Figure 22. Sensitivity analysis by excluding the 12 trials which did not compare the same vaccine with or without adjuvants (using adjuvanted and non-adjuvanted vaccines from different manufacturers, or using antigen-sparing design with reduced antigen dose in adjuvanted vaccines): forest plot showing the ratios of the seroprotection rate (SPR) against H3N2, defined as a ≥1:40 haemagglutinin-inhibition (HI) titer, 21–28 days after the first dose among children who received adjuvanted versus non-adjuvanted trivalent vaccines (TIV) or quadrivalent vaccines (QIV).

Study or Subgroup	adjuvanted Events	adjuvanted Total	Nonadjuvanted Events	Nonadjuvanted Total	Weight	Seroprotection Rate Ratio M-H, Random, 95% CI
**4.7.1 Poor response to non-adjuvanted TIV/QIV (SPR <25%)**						
Vesikari 2011 (6-35 months)	1048	1103	119	995	100.0%	7.94 [6.71, 9.41]
**Subtotal (95% CI)**		**1103**		**995**	**100.0%**	**7.94 [6.71, 9.41]**
Total events	1048		119			
Heterogeneity: Not applicable						
Test for overall effect: Z = 24.02 (P < 0.00001)						
**4.7.2 Moderate response to non-adjuvanted TIV/QIV (SPR 25% to 75%)**						
Vesikari 2011 (36-71 months)	809	834	466	777	100.0%	1.62 [1.53, 1.72]
**Subtotal (95% CI)**		**834**		**777**	**100.0%**	**1.62 [1.53, 1.72]**
Total events	809		466			
Heterogeneity: Not applicable						
Test for overall effect: Z = 16.06 (P < 0.00001)						
**4.7.3 Good response to non-adjuvanted TIV/QIV (SPR >75%)**						
Vesikari 2015 (6-71 months, ATIV primed)	17	17	12	12	40.6%	1.00 [0.87, 1.14]
Vesikari 2015 (6-71 months, TIV primed)	23	23	14	14	59.4%	1.00 [0.90, 1.12]
**Subtotal (95% CI)**		**40**		**26**	**100.0%**	**1.00 [0.92, 1.09]**
Total events	40		26			
Heterogeneity: Tau² = 0.00; Chi² = 0.00, df = 1 (P = 1.00); I² = 0%						
Test for overall effect: Z = 0.00 (P = 1.00)						

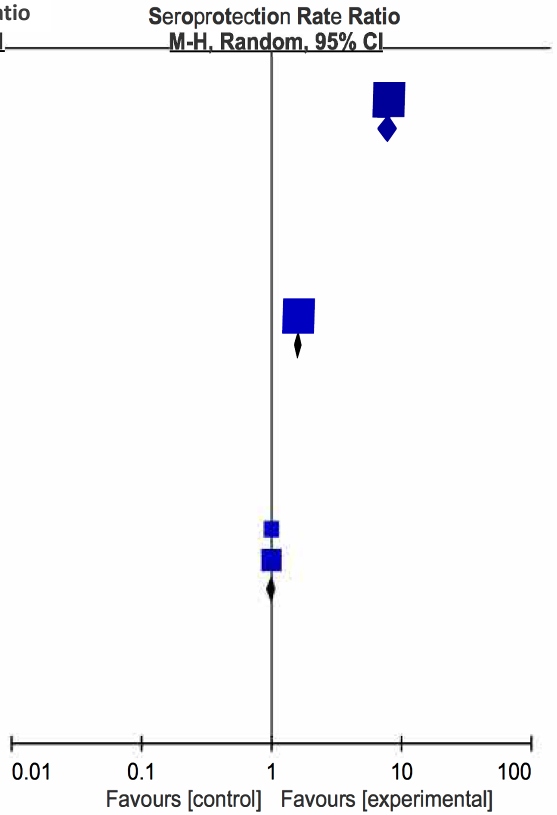

Test for subgroup differences: Chi² = 461.47, df = 2 (P < 0.00001), I² = 99.6%

Supplementary Figure 23. Sensitivity analysis by excluding the 12 trials which did not compare the same vaccine with or without adjuvants (using adjuvanted and non-adjuvanted vaccines from different manufacturers, or using antigen-sparing design with reduced antigen dose in adjuvanted vaccines): meta-regression showing a linear relationship (slope = 0.95, P <0.001) between the ratios of the seroprotection rate against H3N2 after the first dose and the inverse of seroprotection rate in non-adjuvanted arm, with an R^2 of 100%.

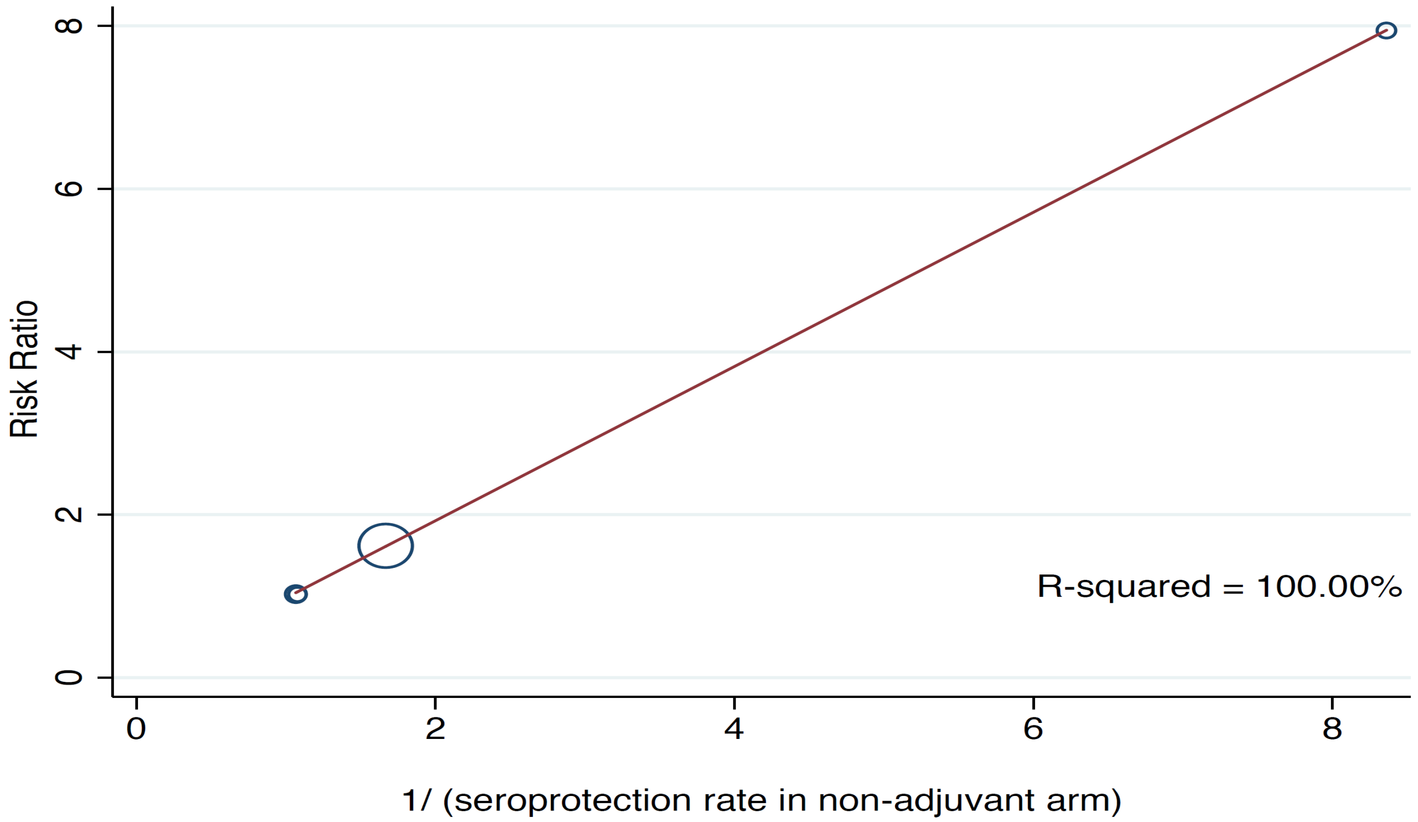

Supplementary Figure 24. Sensitivity analysis by excluding the 12 trials which did not compare the same vaccine with or without adjuvants (using adjuvanted and non-adjuvanted vaccines from different manufacturers, or using antigen-sparing design with reduced antigen dose in adjuvanted vaccines): forest plot showing the ratios of the seroprotection rate (SPR) against influenza B, defined as a ≥1:40 haemagglutinin-inhibition (HI) titer, 21–28 days after the second dose among children who received adjuvanted versus non-adjuvanted trivalent vaccines (TIV) or quadrivalent vaccines (QIV).

Study or Subgroup	adjuvanted Events	adjuvanted Total	Nonadjuvanted Events	Nonadjuvanted Total	Weight	Seroprotection Rate Ratio M-H, Random, 95% CI
**4.9.1 Poor response to non-adjuanted TIV/QIV (SPR <25%)**						
Vesikari 2011 (6-35 months)	965	1103	174	995	100.0%	5.00 [4.36, 5.74]
**Subtotal (95% CI)**		**1103**		**995**	**100.0%**	**5.00 [4.36, 5.74]**
Total events	965		174			
Heterogeneity: Not applicable						
Test for overall effect: Z = 23.07 (P < 0.00001)						
**4.9.2 Moderate response to non-adjuanted TIV/QIV (SPR 25% to 75%)**						
Cruz-Valdez 2018 (6-71 months)	95	114	36	112	30.2%	2.59 [1.96, 3.43]
Vesikari 2011 (36-71 months)	817	834	466	777	41.6%	1.63 [1.54, 1.73]
Zedda 2015 (6-35 months)	25	25	17	30	28.2%	1.74 [1.27, 2.38]
**Subtotal (95% CI)**		**973**		**919**	**100.0%**	**1.91 [1.43, 2.55]**
Total events	937		519			
Heterogeneity: Tau² = 0.05; Chi² = 10.52, df = 2 (P = 0.005); I² = 81%						
Test for overall effect: Z = 4.39 (P < 0.0001)						

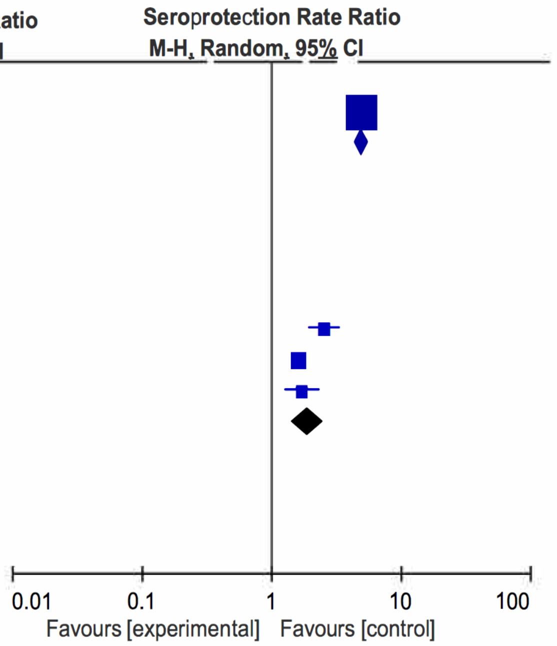

Test for subgroup differences: Chi² = 34.79, df = 1 (P < 0.00001), I² = 97.1%

Supplementary Figure 25. Sensitivity analysis by excluding the 12 trials which did not compare the same vaccine with or without adjuvants (using adjuvanted and non-adjuvanted vaccines from different manufacturers, or using antigen-sparing design with reduced antigen dose in adjuvanted vaccines): meta-regression showing a linear relationship (slope = 0.83, P <0.001) between the ratios of the seroprotection rate against influenza B after the second dose and the inverse of seroprotection rate in non-adjuvanted arm, with an R^2 of 99.84%.

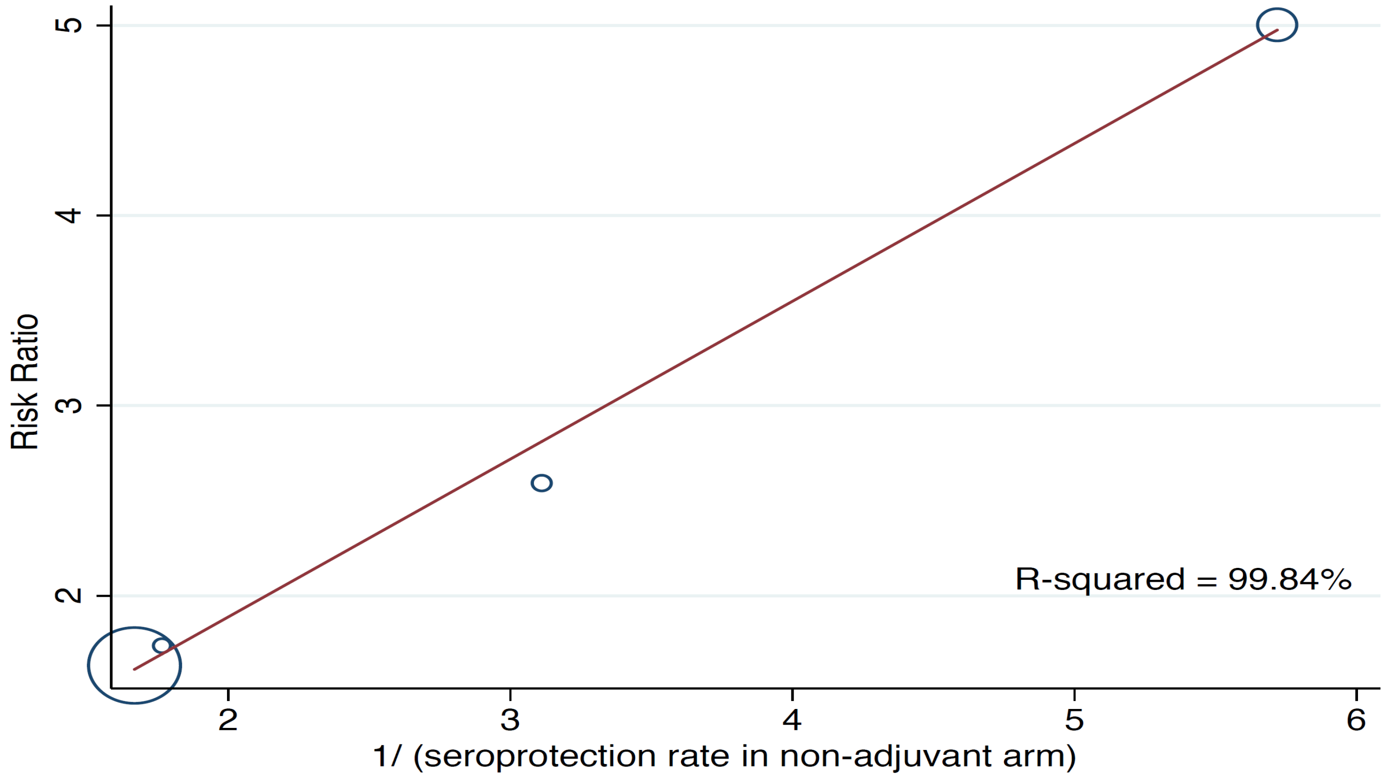

Supplementary Figure 26. Sensitivity analysis by excluding the 12 trials which did not compare the same vaccine with or without adjuvants (using adjuvanted and non-adjuvanted vaccines from different manufacturers, or using antigen-sparing design with reduced antigen dose in adjuvanted vaccines): forest plot showing the risk ratios of serious adverse events during the follow-up time after the vaccinations among children who received adjuvanted versus non-adjuvanted trivalent vaccines (TIV) or quadrivalent vaccines (QIV).

	Experimental		Control			Risk Ratio
Study or Subgroup	Events	Total	Events	Total	Weight	M-H, Random, 95% CI
Block 2012	6	161	16	161	5.5%	0.38 [0.15, 0.93]
Cruz-Valdez 2018	0	140	0	137		Not estimable
Nassim 2012	6	334	6	334	3.6%	1.00 [0.33, 3.07]
Vesikari 2011	122	2012	169	1846	90.5%	0.66 [0.53, 0.83]
Vesikari 2015	1	33	0	24	0.5%	2.21 [0.09, 51.93]
**Total (95% CI)**		**2680**		**2502**	**100.0%**	**0.66 [0.53, 0.81]**
Total events	135		191			

Heterogeneity: Tau² = 0.00; Chi² = 2.56, df = 3 (P = 0.46); I² = 0%
Test for overall effect: Z = 3.89 (P = 0.0001)

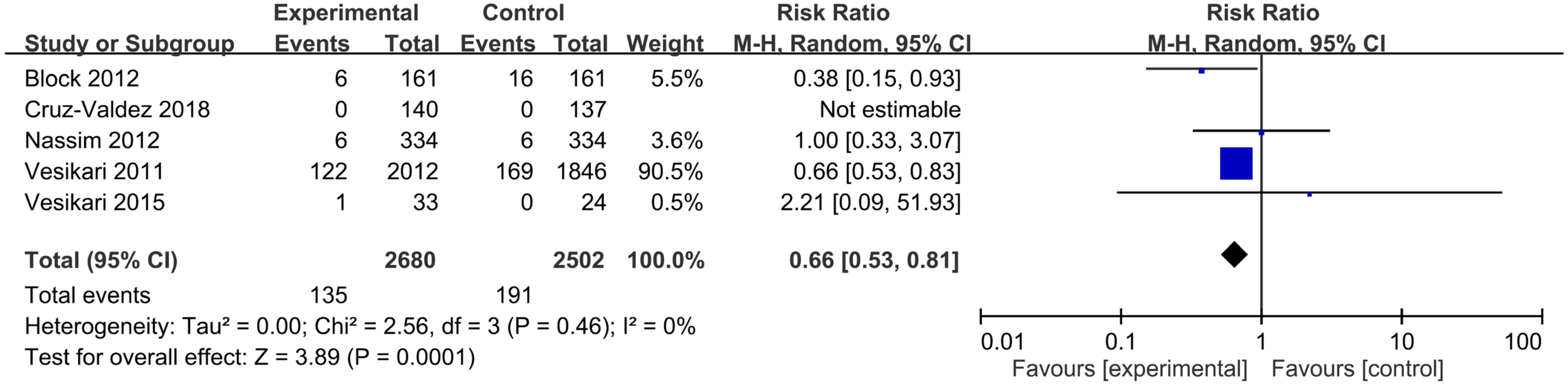

Supplementary Figure 27. Sensitivity analysis by excluding the 12 trials which did not compare the same vaccine with or without adjuvants (using adjuvanted and non-adjuvanted vaccines from different manufacturers, or using antigen-sparing design with reduced antigen dose in adjuvanted vaccines): forest plot showing the risk ratios of neurological events during the follow-up time after the vaccinations among children who received adjuvanted versus non- adjuvanted trivalent vaccines (TIV) or quadrivalent vaccines (QIV).

	Experimental		Control			Risk Ratio
Study or Subgroup	Events	Total	Events	Total	Weight	M-H, Random, 95% CI
Cruz-Valdez 2018	0	140	0	137		Not estimable
Vesikari 2011	1	2012	0	1846	100.0%	2.75 [0.11, 67.53]
Vesikari 2015	0	33	0	24		Not estimable
Zedda 2015	0	43	0	39		Not estimable
**Total (95% CI)**		**2228**		**2046**	**100.0%**	**2.75 [0.11, 67.53]**
Total events	1		0			

Heterogeneity: Not applicable
Test for overall effect: Z = 0.62 (P = 0.54)

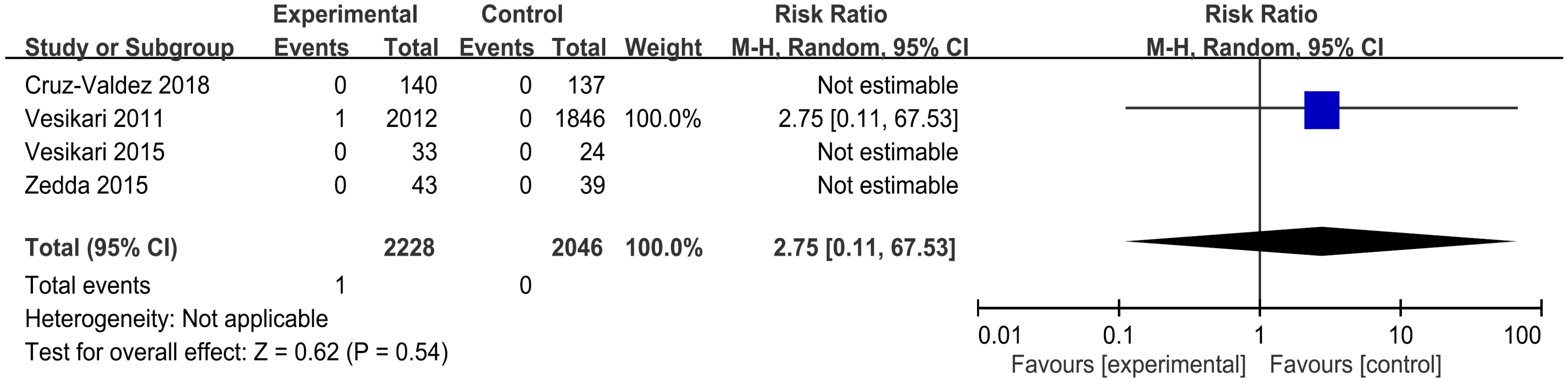

Supplementary Figure 28. Sensitivity analysis by excluding 2 data points (both reported in Diallo 2018) with an inverse of seroprotection rate in non-adjuvanted arm larger than 5: Meta-regression still showed a linear relationship (slope = 0.90, P <0.001) between the ratios of seroprotection rates against H1N1 after the first dose and the inverse of seroprotection rate in non-adjuvanted arm, with an R^2 of 98.67%.

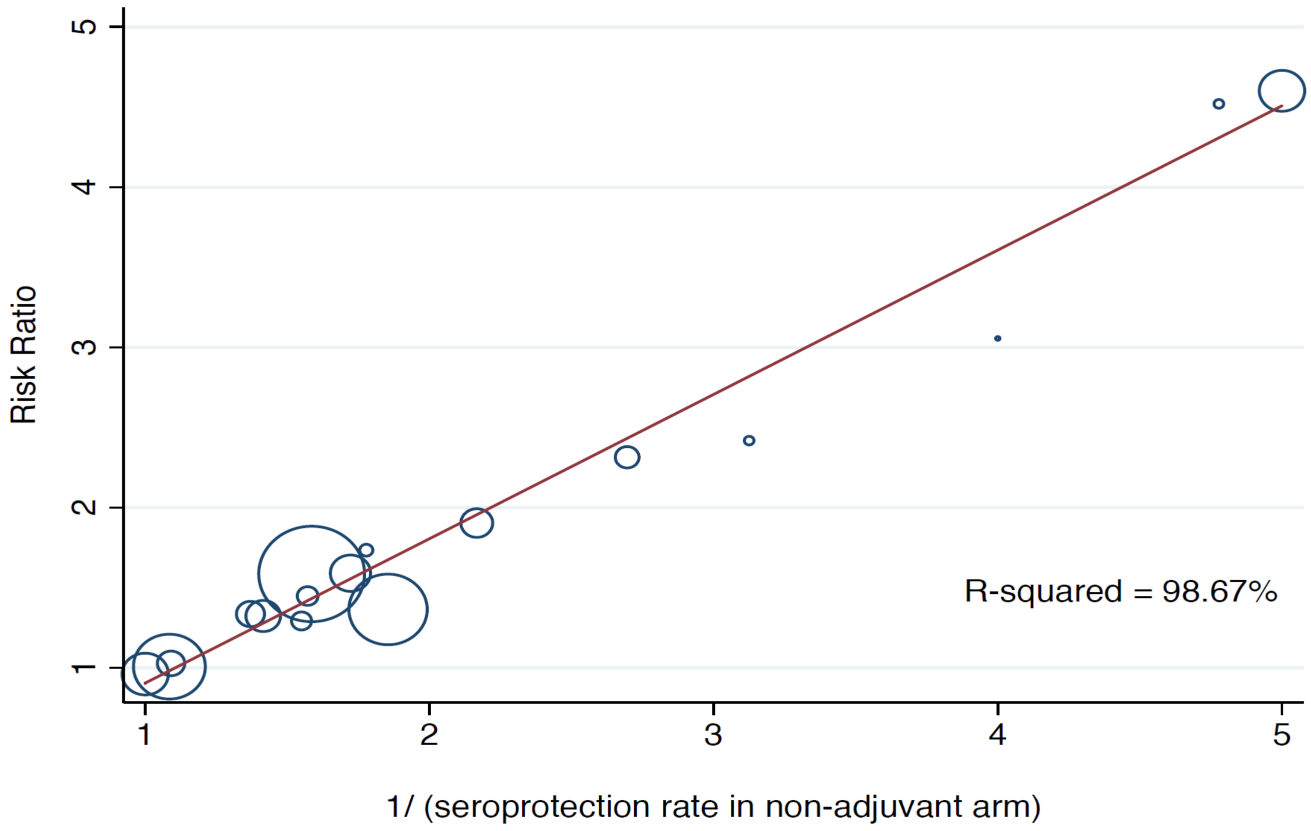

Supplementary Figure 29. Sensitivity analysis by excluding 2 data points (Vesikari 2011 and Della Cioppa 2011) with an inverse of seroprotection rate in non-adjuvanted arm larger than 5: Meta-regression results became imprecise (slope = 0.47, P=0.075) between the ratios of seroprotection rates against H3N2 after the first dose and the inverse of seroprotection rate in non-adjuvanted arm, with an R^2 of 42.98%, after the sample size decreased to only seven.

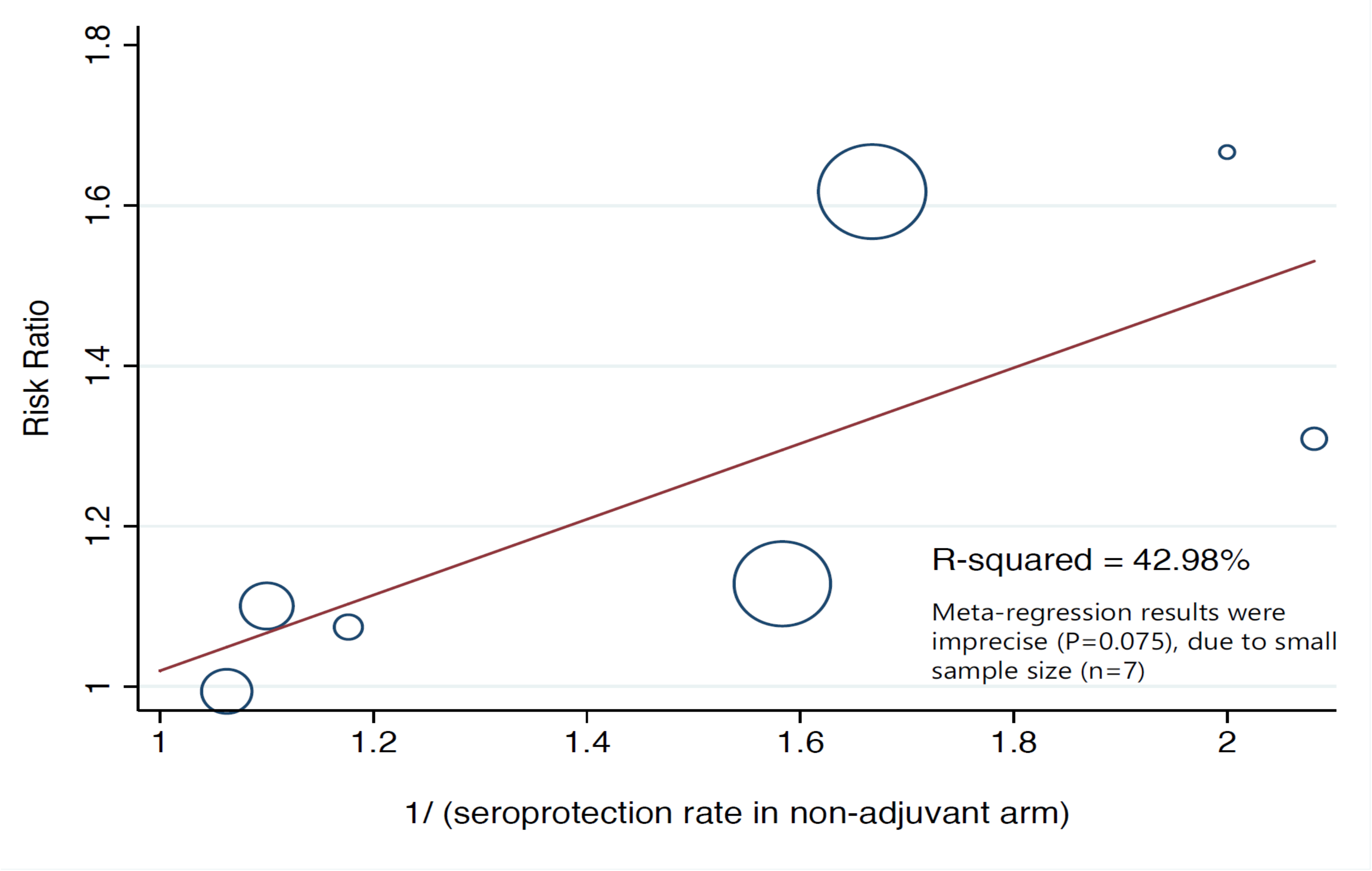

Supplementary Figure 30. Sensitivity analysis by excluding 2 data points (Vesikari 2011 and Della Cioppa 2011), with an inverse of seroprotection rate in non-adjuvanted arm larger than 5: Meta-regression still showed a linear relationship (slope = 0.86, P <0.001) between the ratios of seroprotection rates against influenza B after the first dose and the inverse of seroprotection rate in non-adjuvanted arm, with an R^2 of 96.21%.

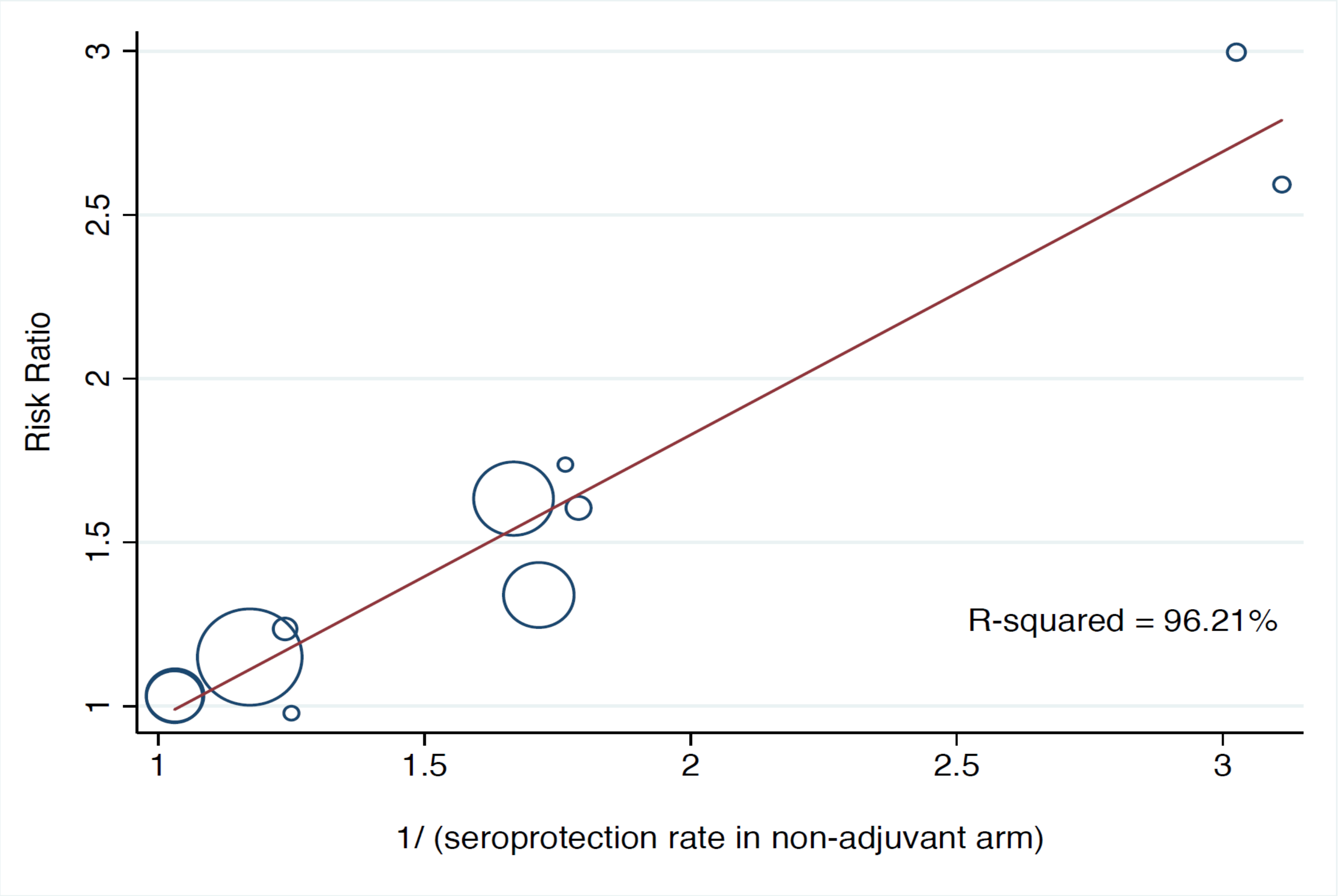

Supplementary References

1. Vesikari T, Pellegrini M, Karvonen A, et al. Enhanced immunogenicity of seasonal influenza vaccines in young children using MF59 adjuvant. Pediatr Infect Dis J. 2009;28(7):563-571.
2. Vesikari T, Groth N, Karvonen A, Borkowski A, Pellegrini M. MF59-adjuvanted influenza vaccine (FLUAD) in children: safety and immunogenicity following a second year seasonal vaccination. Vaccine. 2009;27(45):6291-6295.
3. Arguedas A, Soley C, Abdelnour A, et al. Assessment of the safety, tolerability and kinetics of the immune response to A/H1N1v vaccine formulations with and without adjuvant in healthy pediatric subjects from 3 through 17 years of age. Hum Vaccin. 2011;7(1):58-66.
4. Della Cioppa G, Vesikari T, Sokal E, Lindert K, Nicolay U. Trivalent and quadrivalent MF59-adjuvanted influenza vaccine in young children: a dose- and schedule-finding study. Vaccine. 2011;29(47):8696-8704.
5. Vesikari T, Knuf M, Wutzler P, et al. Oil-in-water emulsion adjuvant with influenza vaccine in young children. N Eng J Med. 2011;365(15):1406-1416.
6. Block SL, Ruiz-Palacios GM, Guerrero ML, Beygo J, Sales V, Holmes SJ. Dose-range study of MF59-adjuvanted versus nonadjuvanted monovalent A/H1N1 pandemic influenza vaccine in six- to less than thirty-six-month-old children. Pediatr Infect Dis J. 2012;31(7):e92-98.
7. Langley JM, Reich D, Aggarwal N, et al. Randomized, multicenter trial of a single dose of AS03-adjuvanted or unadjuvanted H1N1 2009 pandemic influenza vaccine in children 6 months to <9 years of age: safety and immunogenicity. Pediatr Infect Dis J. 2012;31(8):848-858.
8. Nassim C, Christensen S, Henry D, Holmes S, Hohenboken M, Kanesa-Thasan N. Identification of antigen and adjuvant doses resulting in optimal immunogenicity and antibody persistence up to 1 year after immunization with a pandemic A/H1N1 influenza vaccine in children 3 to < 9 years of age. Pediatr Infect Dis J. 2012;31(4):e59-65.
9. Nolan T, Bravo L, Ceballos A, et al. Enhanced and persistent antibody response against homologous and heterologous strains elicited by a MF59-adjuvanted influenza vaccine in infants and young children. Vaccine. 2014;32(46):6146-6156.
10. Nolan T, Roy-Ghanta S, Montellano M, et al. Relative efficacy of AS03-adjuvanted pandemic influenza A(H1N1) vaccine in children: results of a controlled, randomized efficacy trial. J Infect Dis. 2014;210(4):545-557.
11. Knuf M, Leroux-Roels G, Rumke H, et al. Immunogenicity and safety of cell-derived MF59-adjuvanted A/H1N1 influenza vaccine for children. Hum Vaccin Immunother. 2015;11(2):358-376.
12. Solares AR, Aragon CG, Pivaral RU, et al. Safety and immunogenicity profiles of an adjuvanted seasonal influenza vaccine in Guatemalan children. J Infect Dev Ctries. 2014;8(9):1160-1168.
13. Vesikari T, Forsten A, Arora A, Tsai T, Clemens R. Influenza vaccination in children primed with MF59-adjuvanted or non-adjuvanted seasonal influenza vaccine. Hum Vaccin Immunother. 2015;11(8):2102-2112.

14. Zedda L, Forleo-Neto E, Vertruyen A, et al. Dissecting the immune response to MF59-adjuvanted and nonadjuvanted seasonal influenza vaccines in children less than three years of age. Pediatr Infect Dis J. 2015;34(1):73-78.

15. Vesikari T, Kirstein J, Devota Go G, et al. Efficacy, immunogenicity, and safety evaluation of an MF59-adjuvanted quadrivalent influenza virus vaccine compared with non-adjuvanted influenza vaccine in children: a multicentre, randomised controlled, observer-blinded, phase 3 trial. Lancet Respir Med. 2018;6(5):345-356.

16. Cruz-Valdez A, Valdez-Zapata G, Patel SS, et al. MF59-adjuvanted influenza vaccine (FLUAD) elicits higher immune responses than a non-adjuvanted influenza vaccine (Fluzone): A randomized, multicenter, Phase III pediatric trial in Mexico. Hum Vaccin Immunother. 2018;14(2):386-395.

17. Diallo A. *et al.* Immunogenicity and safety of MF59-adjuvanted and full-dose unadjuvanted trivalent inactivated influenza vaccines among vaccine-naïve children in a randomized clinical trial in rural Senegal. *Vaccine*. **36,** 6424-6432 (2018).

18. Waddington CS, Walker WT, Oeser C, et al. Safety and immunogenicity of AS03B adjuvanted split virion versus non-adjuvanted whole virion H1N1 influenza vaccine in UK children aged 6 months-12 years: open label, randomised, parallel group, multicentre study. BMJ. 2010;340:c2649.
