# Supplementary material for: Oil-in-water emulsion adjuvants for pediatric influenza vaccines: a systematic review and meta-analysis
Source: Nat Commun. 2020 Jan 16;11:315. doi: 10.1038/s41467-019-14230-x (PMC6965081; doi:10.1038/s41467-019-14230-x)
Supplement: Supplementary file 3 — Reporting Summary [file 41467_2019_14230_MOESM3_ESM.pdf]

nature research

Corresponding author(s): Chi-Tai Fang

Reporting Summary

Nature Research wishes to improve the reproducibility of the work that we publish. This form provides structure for consistency and transparency in reporting. For further information on Nature Research policies, see Authors & Referees and the Editorial Policy Checklist.

Statistical parameters

When statistical analyses are reported, confirm that the following items are present in the relevant location (e.g. figure legend, table legend, main text, or Methods section).

n/a	Confirmed	
☐	☒	The exact sample size (n) for each experimental group/condition, given as a discrete number and unit of measurement
☐	☒	An indication of whether measurements were taken from distinct samples or whether the same sample was measured repeatedly
☐	☒	The statistical test(s) used AND whether they are one- or two-sided<br>*Only common tests should be described solely by name; describe more complex techniques in the Methods section.*
☐	☒	A description of all covariates tested
☐	☒	A description of any assumptions or corrections, such as tests of normality and adjustment for multiple comparisons
☐	☒	A full description of the statistics including central tendency (e.g. means) or other basic estimates (e.g. regression coefficient) AND variation (e.g. standard deviation) or associated estimates of uncertainty (e.g. confidence intervals)
☐	☒	For null hypothesis testing, the test statistic (e.g. F, t, r) with confidence intervals, effect sizes, degrees of freedom and P value noted<br>*Give P values as exact values whenever suitable.*
☒	☐	For Bayesian analysis, information on the choice of priors and Markov chain Monte Carlo settings
☒	☐	For hierarchical and complex designs, identification of the appropriate level for tests and full reporting of outcomes
☐	☒	Estimates of effect sizes (e.g. Cohen's d, Pearson's r), indicating how they were calculated
☐	☒	Clearly defined error bars<br>*State explicitly what error bars represent (e.g. SD, SE, CI)*

Our web collection on statistics for biologists may be useful.

Software and code

Policy information about availability of computer code

Data collection	Review Manager (RevMan) version 5.3 (Copenhagen: The Nordic Cochrane Centre, The Cochrane Collaboration, 2014)
Data analysis	Review Manager (RevMan) version 5.3 (Copenhagen: The Nordic Cochrane Centre, The Cochrane Collaboration, 2014)

For manuscripts utilizing custom algorithms or software that are central to the research but not yet described in published literature, software must be made available to editors/reviewers upon request. We strongly encourage code deposition in a community repository (e.g. GitHub). See the Nature Research guidelines for submitting code & software for further information.

Data

Policy information about availability of data

All manuscripts must include a data availability statement. This statement should provide the following information, where applicable:

- Accession codes, unique identifiers, or web links for publicly available datasets
- A list of figures that have associated raw data
- A description of any restrictions on data availability

All the data analysed in this study are included in the published article.

Field-specific reporting

Please select the best fit for your research. If you are not sure, read the appropriate sections before making your selection.

- [x] Life sciences
- [ ] Behavioural & social sciences
- [ ] Ecological, evolutionary & environmental sciences

For a reference copy of the document with all sections, see nature.com/authors/policies/ReportingSummary-flat.pdf

Life sciences study design

All studies must disclose on these points even when the disclosure is negative.

Sample size	This is a meta-analyses of all eligible studies identified by searching PubMed, EMBASE, Cochrane, CINAHL, Web of Science, Scopus, and Google Scholar, along with reports completed in the clinical trials registry database (ClinicalTrials.gov) before August 31, 2018.
Data exclusions	We excluded studies that did not compare oil-in-water adjuvanted influenza vaccines versus the non-adjuvanted counterparts.
Replication	This is a meta-analysis. The authors have repeated the statistical analyses to verify the accuracy of results.
Randomization	This is a meta-analysis of randomised clinical trials.
Blinding	With the exception of Zedda (2015), all included randomised trials reported the use of blinding.

Reporting for specific materials, systems and methods

Materials & experimental systems

n/a	Involved in the study
x	Unique biological materials
x	Antibodies
x	Eukaryotic cell lines
x	Palaeontology
x	Animals and other organisms
	x Human research participants

Methods

n/a	Involved in the study
x	ChIP-seq
x	Flow cytometry
x	MRI-based neuroimaging

Human research participants

Policy information about studies involving human research participants

Population characteristics	Clinical trials were eligible if they compared the efficacy or immunogenicity of inactivated influenza vaccines with an oil-in-water adjuvant versus that of inactivated vaccines without an adjuvant. The trials must have been conducted in healthy children younger than 9 years.
Recruitment	This is a meta-analysis of randomised clinical trials. The study protocol was registered with the PROSPERO registry (CRD42018093436). We searched PubMed for clinical trials published prior to August 31, 2018, using the following keywords: adjuvant AND influenza vaccine AND (infant OR child), filtered by clinical trial, full text and English language. We searched ClinicalTrials.gov for clinical trials registered and completed before August 31, 2018, using the following keywords: adjuvant AND influenza vaccine AND human, filtered by age younger than 9 years and intervention studies.
